# Benefit sharing in genomic and biobanking research in Uganda: Perceptions of researchers and research ethics committee members

**DOI:** 10.3389/fgene.2022.1037401

**Published:** 2022-11-17

**Authors:** Erisa Sabakaki Mwaka, Godfrey Bagenda, Deborah Ekusai Sebatta, Sylvia Nabukenya, Ian Munabi

**Affiliations:** Department of Anatomy, School of Biomedical Sciences, College of Health Sciences, Makerere University, Kampala, Uganda

**Keywords:** benefit sharing, genomics, biobanking, research, research ethic committees, researchers

## Abstract

**Background:** Genomic and biobanking research has increased in Africa over the past few years. This has raised pertinent ethical, legal, and societal concerns for stakeholders such as sample or data ownership, commercialization, and benefit sharing. There is limited awareness of the concept of benefit sharing by stakeholders in sub-Saharan Africa.

**Objective:** This study aimed to explore the perceptions of researchers and research ethics committee members on benefit sharing in international collaborative genomic and biobanking research.

**Methods:** Qualitative in-depth interviews were conducted with 15 researchers and 19 research ethics committee members. A thematic approach was used to interpret the results.

**Results:** Six themes emerged from the data and these included perceptions on the benefits of genomic and biobanking research; discussion of benefit sharing with participants during the informed consent process; legal implications of benefit sharing and the role of material transfer agreements; equity and fairness in sharing the benefits of genomic research; perceived barriers to fair benefit sharing; and recommendations for fostering fair and equitable benefit sharing in genomic and biobanking research. Most respondents clearly understood the various forms of benefits of genomic and biobanking research and opined that such benefits should be fairly and equitably shared with low and middle-income country researchers and their institutions, and research communities. The perceived barriers to the fair benefit sharing unfavorable include power disparities, weak research regulatory frameworks, and lack of scientific integrity.

**Conclusion:** Overall, respondents believed that the distribution of the advantages of genomic and biobanking research in North-South collaborative research was not equitable nor fair, and that the playing field was not leveled. Therefore, we advocate the following for fair and equitable benefit sharing: Building the capacities and empowering research scientists in developing nations; strengthening regulatory frameworks and extending the purview of the research ethics committee in the development and implementation of material transfer agreements; and meaningfully involving local research communities in benefit sharing negotiations.

## 1 Introduction

The African continent remains grossly underrepresented in genomic research with less than 2% representation of the human genomes that have so far been analyzed (Sirugo et al., 2019), despite hosting human population with high levels of genetic variation and diversity. These populations are spread over the large area of the African continent with little or no inter-mixing with other non-African populations, which increases the chances of finding novel variants likely to contribute to specific diseases (Tishkoff et al., 2009; Gurdasani et al., 2015; Editorial, 2020). Some of the population-wide genomic research projects in Africa are generating huge amounts of genomic data on the African population for example; the Human Heredity and Health in Africa (H3Africa) initiative (H3Africa, 2014), the African genome variation project (Gurdasani et al., 2015; African genome variation project, 2022), and the Neuropsychiatric Genetics of African Populations-Psychosis (Stevenson et al., 2019). H3Africa is a consortium that was initiated in 2010 to empower Africa researchers in genomic sciences, establish and nurture effective collaborative partnerships among African researchers based on the African continent and generate valuable data that could be used to improve global health (H3Africa, 2014). These research projects have created biobanks containing large quantities of human biological materials and data. As a result of these large biobanks, genomic and biobanking research (GBR) is fast becoming one of the major ethical challenges for many of the international collaborative research studies in sub-Saharan Africa due to the potential ethical issues around equity and fairness.

The need for translation of genomic knowledge into products and policies, has raised several pertinent and legitimate concerns for stakeholders that include: data and sample ownership, sharing of biological samples and or data with commercial entities, intellectual property arrangements, and benefit sharing (Chadwick et al., 2021; Marshall et al., 2022). Whereas some researchers and research regulators feel that commercialization of research outputs is inevitable for the benefit of humankind (Vaz et al., 2018), many feel it is unethical to profit from a donor’s data and samples (Critchley et al., 2021). This notion probably stems from the historical unidirectional export of biological samples and data from the developing world to the developed world, often with no benefit to institutions, scientists, communities and health priorities of the developing countries generating the samples and or data (Wonkam et al., 2011). This situation is further exacerbated by the legal cases involving commercialization of human body parts. For example, the John Moore Case (in United States), where a dispute in sharing benefits was the subject of a legal case in which the supreme court in the State of California ruled that people do not have rights to share in the profits earned from research performed on their biological samples (Ivey, 1990; Leeds, 1991). Such incidents could pose a significant risk to public trust in GBR (Critchley et al., 2013; Caulfield and Ogbogu, 2015; Critchley et al., 2015; Nicol et al., 2016). Stakeholders in source countries expect equitable and fair sharing with benefits trickling down to the community (Vaz et al., 2018).

This manuscript explores the issues surrounding benefit sharing in GBR, a topic that has not been adequately explored in sub-Saharan Africa. Discussions on benefit sharing in genetics research came to prominence during the Human Genome Project when the Human Genome Organization (HUGO) Ethics Committee raised concerns about the possibility of poor nations missing out on the benefits of the project (HUGO Ethics Committee, 2000). In sub-Saharan Africa, the importance of having additional debate on benefit sharing recently gained prominence with the news of the Wellcome Trust Sanger Institute scandal in which it is alleged that a proposal to develop a commercial gene chip was in the making, without proper legal agreements with partner institutions and consent of African donors, whose DNA and data were used to develop the chip (Stokstad, 2019). This alleged scandal provoked prolonged discussion within the H3Africa Consortium on the meanings, implications and impact of sharing biological samples and data with commercial entities, benefit sharing, and appropriate consent in GBR research.

Several definitions of benefit sharing have been suggested, including by Schoeder (Schroeder, 2007) who defined benefit sharing as, “the action of giving a portion of advantages/profits derived from the use of human genetic resources to the resource providers to achieve justice in exchange, with a particular emphasis on the clear provision of benefits to those who may lack reasonable access to resulting healthcare products and services without providing unethical inducements.” There is limited awareness of the concept of benefit sharing by stakeholders in sub-Saharan Africa, and most countries in Africa lack appropriate laws and regulation to guide benefit sharing (de Vries et al., 2017; Vaz et al., 2018; Munung and de Vries, 2020). This study set out to explore the perceptions of researchers and research ethics committee members on benefit sharing in international collaborative GBR. By exploring perceptions of researchers and research regulators on benefit sharing, we hope to contribute towards developing a framework to guide processes for fair sharing of research benefits in Uganda. Stakeholder opinions are important in the development of best practices guidelines for GBR (Husedzinovic et al., 2015).

## 2 Materials and methods

This was a qualitative exploratory study where data was collected using in-depth interviews. This study is part of a bigger on-going study that is exploring the perceptions and experiences of various stakeholders on informed consent processes for genomic research in Uganda (Mwaka et al., 2021). For this paper, we present findings on researchers and research ethics committee (REC) members’ perceptions on benefit sharing in international collaborative GBR.

### 2.1 Study setting

The study was conducted at Makerere University College of Health Sciences (MakCHS) and 10 RECs that had experience in conducting ethical review of genetic and genomic research. Makerere College of Health Sciences is one of the nine constituent colleges at Makerere University in Uganda. As the largest and most research-intensive university in Uganda, Makerere University has tremendously impacted medical education and research capacity development in Uganda and the rest of Africa. There are 29 RECs in Uganda accredited by Uganda National Council for Science and Technology (UNCST) (UNCST, 2022), the national regulatory body for all research in Uganda. Of these, only 10 had experience with reviewing genetic/genomic research and were selected for inclusion in this study. At least one member was purposively selected from each REC, including three community representatives.

### 2.2 Participants

Participants were either researchers or REC members actively involved in the conduct and ethical review of genetic/genomic research respectively. The researchers were principal investigators of protocols involving host genetics/genomics that were approved by UNCST for the period 2012–2017. Only one researcher was a member of a REC. Uganda National Council for Science and Technology provides regulatory oversight of all research activities in the country; and per local regulations, all protocols approved by accredited RECs are submitted to UNCST for approval and registration. We searched archived research protocols approved by UNCST for the period 2012–2017. Only investigators based at MakCHS, and affiliate research institutes were eligible. A list of 23 investigators was generated and all were invited to participate in the study but only 15 consented and participated in the study, of which three were H3Africa principal investigators. The number of researchers conducting genetics and genomic research at MakCHS is not known, however it is important to note that there are several masters and PhD level scientists that are training in genetic science and bioinformatics, mainly sponsored by the H3Africa initiative. The REC members were purposively selected based on their expertise in reviewing genetic/genomic research protocols.

### 2.3 Data collection

Thirty-four in-depth interviews were conducted between February to June 2019 by a team of five researchers that included one bioethicist with training in medicine (ESM), a social scientist with experience in qualitative research methods (DES), a medical educator with experience in qualitative data analysis (IM) and two research assistant who are also graduate students of bioethics (GB and SN). A team of four researchers (ESM, DES, GB, and SN), conducted all interviews to ensure consistency. Prior to starting of the study, the research team was trained on the protocol to ensure that they internalized and understood the study well. Data were collected using an in-depth interview guide that was developed by ESM and DES and consisted of open-ended questions that explored researchers’ perceptions on benefit sharing in GBR (see Supplementary Materials). The interview guides were piloted and revised prior to the full data collection process. All interviews were conducted in English, audio recorded alongside detailed note taking, and later transcribed verbatim. Data for the REC members was conducted until theoretical saturation. Transcripts were cleaned before the analytical process. Field notes were also taken during the interviews. On average, interviews lasted between 45–60 min. Debriefing meetings were held by the research team at the end of each interview to ensure completeness and to also review preliminary perspectives that had arisen.

### 2.4 Data management and analysis

Data analysis was conducted continuously throughout the study using a thematic approach (Braun and Clarke, 2006; Fereday and Muir-Cochrane, 2006). The first step of the analysis involved reading of all transcripts to familiarize, mark and memo the data. Two of the authors (DES and ESM) then developed a codebook and coding framework. Themes were then generated both deductively, based on our prior analytic framework derived from the literature on benefit sharing in GBR, as represented in the interview guide; and inductively, by considering the new themes that emerged from the text. Then ESM, DES and SN examined the themes for patterns until consensus was achieved on the final themes after several iterative discussions. The emergent themes were then compared with the available literature to ensure that the final themes represent a true reflection of respondents’ pespectives on benefit sharing in GBR. This contributed to the transferability of the data and allowed for extrapolation of study findings to other similar settings (Singh and Moodley, 2021). Codes and themes were organised using NVivo 12 software (QSR International Pty Ltd., 2014). The authors identified quotes in the respondents’ transcripts that were congruent with the overarching themes. Some transcripts (several respondents objected) were returned to interviewees for verification to ensure that the collected data was a true reflection of their statements on benefit sharing and the ethical issues surrounding GBR. The authors had a well-established relationship with all interviewees because they are all involved in the conduct and/or regulation of research at Makerere University and affiliate institutions.

Regarding research reflexivity, we were aware that when interviewing research stakeholders we needed to try and remain neutral, setting aside our own views and to listen from the respondents’ perspective. It was however difficult for us to be totally objective and not relate to our experiences because of our interest and active participation in benefit sharing discussions at the local and international levels.

## 3 Ethics approval

Ethics approval was obtained from the Makerere University School of Biomedical Sciences Higher Degrees and Research Ethics Committee (SBSHD-REC 517) followed by clearance by Uganda National Council for Science and Technology (SS 4490). Written informed consent was obtained from all participants prior to the interview. Data was kept securely, and all recordings and transcripts were de-identified, assigned special codes and stored on a password-protected computer. No participant identifying information was published.

## 4 Results

### 4.1 Demographic characteristics

There were 34 interviewees consisting of 15 researchers and 19 REC members. Most researchers were male (12/15), and all were involved in international collaborative GBR. Five of the researchers were clinical researchers, six were clinical epidemiologists and three were basic genetic scientists (Table 1). Only one interviewee had formal training in clinical genetics. The average research experience of the interviewees was 12 years (SD 1.2, range: 3–22 years).

**TABLE 1:** Demographic information.

Researchers		REC members	
Gender			
Male	12	Male	12
Female	3	Female	7
Highest level of education		Highest level of education	
Masters	6	Masters	6
PhD	9	PhD	9
		Bachelors	2
		Certificate/Diploma	4
Role in research		Role on REC	
Clinical Researcher	5	Chair	5
Basic Scientist	3	Vice chair	4
Clinical Epidemiologist	6	Member	7
Clinical Geneticist	1	Community representative	3
Research experience (years)		Duration as REC Member	
0–5	2	0–5	8
6–10	3	6–10	8
11–15	8	11–15	1
>15	2	>15	2

Research ethics committee members comprised of 12 males and seven females, these included five REC chairs, four vice-chairs, seven regular members and four community representatives. The REC members had an average REC experience of 10 years (3–17 years). Demographic data are summarized in Table 1.

### 4.2 Thematic areas emerging from the data

The following themes emerged from the data: 1) perceptions on the benefits of GBR; 2) discussion of benefit sharing with participants during the informed consent process; 3) legal implications of benefit sharing and the role of material transfer agreements (MTA); 4) equity and fairness in sharing the benefits of GBR; 5) perceive barriers to fair benefit sharing; 6) the way forward: fostering fair and equitable benefit sharing in GBR. The dataset to this work is accessible online (Mwaka et al., 2022).

### 4.3 Theme 1: Perceptions on the benefits of GBR

There are several forms of benefits that accrue from GBR. Some respondents noted that GBR helps elucidate the genetic causes of diseases, as such; it contributes to the development of diagnostic equipment and therapeutic interventions, optimization of drug dosaging, and is key in disease prevention. One respondent pointed out that GBR aims to benefit the wider community as illustrated below:

…. well, if the findings are really suggesting something then this is something that can guide future interventions that can benefit the community at large if it is significant. So eventually it does benefit because we get to understand how these diseases happen (Researcher 14, Female).

The other benefits of GBR included feedback of research results; the creation of jobs and employment opportunities for host communities; joint publications and acknowledgement in scholarly works; infrastructural capacity building; opportunities for research funding; and human resource capacity building as stated in this quote:

…. they (foreign collaborators) should be encouraged to come and develop capacity here such that instead of us sending the samples to them, they come and do the research here and, in the process, build further capacity here to do this research either in terms of getting facilities or in terms of training people to do that sort of research (Researcher 8, Male).

Regarding monetary benefits, respondents mentioned the sharing of biological samples and associated data with commercial entities. Most respondents (18/34) emphasized that these benefits should be equitably shared with local institutions, researchers, individual research participants and research communities.

Like the CAPRISA (Centre for the AIDS Programme of Research in South Africa) people did for that lady whom they [researchers] got an antibody from. Should a Ugandan buy a patentable product from a biological sample? To me the benefits should go back to that person, that person where the sample came from should also be included. The policy should be revised accordingly if it does not clearly stipulate those issues (Researcher 07, Male).

Another respondent opined:

And whichever research we want to do, we want it to benefit the population from which [the data] has been collected from (Researcher 14, Female).

One researcher and two REC members were however uncertain about how the benefits of GBR should be shared with research participants and communities. They surmised that ethical and legal frameworks in Uganda do not offer adequate protection to the local research community, thus contributing to the inequity and exploitation currently observed in GBR. One respondent raised fears of potential disputes that could arise between sample owners and patent holders.

But on that point, supposing I get a patent and I am paid, that issue of ownership becomes another problem. Who owns it (the patent)? Because if I agree you are the donor, you are the owner of the sample but sometimes the patent comes from analysis, putting together this and comparing with [that]. I think this one is a drug target, or we can create a vaccine then I patent it. Now you, see? (Researcher 09, Male).

### 4.4 Theme 2: Discussion of benefit sharing with participants during the informed consent process

Valid informed consent demands that research participants voluntarily provide samples for research purposes after receiving adequate relevant information about the study in a language that is commensurate with their level of literacy. Some respondents pointed out that information on benefit sharing should be clearly included in the informed consent documents. They indicated that this should be considered even if there may not be any anticipated tangible benefits. They noted that this was not only an ethical imperative but also one way of building confidence and trust among research communities.

You know, you need to revise those aspects and those should come in a separate section [and should state] that, “Should anything of value come out of your sample then you will be rewarded.” I think that revision is necessary although by and large the way science is, 90% of the time there is nothing which can come out (Researcher 07, Male).

### 4.5 Theme 3: Legal implications of benefit sharing and the role of MTAs

All respondents believed that MTAs are key in the exchange of samples in GBR. They indicated that MTAs should contain details of the terms and conditions that govern sharing samples. They noted that issues concerning benefit sharing, ownership of samples and their derivatives, commercialization, intellectual property rights and authorship should all be included in MTAs. One researcher pointed out that at times local researchers and institutions just sign MTAs provided by foreign collaborating institutions without even scrutinizing them.

They do not know what they are signing and what they are giving away, but I think a lot of effort should go into that material transfer agreement and that material transfer agreements should be enforced because that’s where the other things you are talking about commercialization, intellectual property is guided and of course it transfers the patient’s consent because all of those must be taken care of because when transferring the material (Researcher 09, Male).

Some respondents (six researchers and six REC members) felt that researchers and research institutions from low-resource settings are not empowered enough to bargain favorably during MTA negotiations. They also felt that Uganda lacks appropriate enabling ethical and legal frameworks to protect the interests of local scientists and research institutions. They contended that the national ethics guidelines are vague and do not prescribe how exactly research benefits should be shared.

One researchers asserted that it is imperative that MTAs are reviewed by the RECs to ensure that the interests of local stakeholders are adequately protected.

Yes, again if samples are shipped and the investigator has no control, then really how will the community benefit from where the samples were collected? Then also the authorship, like getting the credit for having done the research here is also important to motivate the researcher. Yes, I think it should be regulated maybe through an MTA yes and maybe while we are seeking ethical approval such issues should be clearly laid out for the researcher …. for (their) protection …. and the samples and the data collected (Researcher 14, Female).

Another issue that researchers have to be cognizant of are the differences in the regulations governing GBR across national boundaries. One respondent indicated that knowledge of the provisions of the ethical and legal frameworks that govern GBR in the collaborators country is important for the protection of the researchers, research participants and during benefit sharing negotiations, as illustrated below:

People can make discoveries from this research and they can make money out of it, so we need to know whether the rights of these people who have participated in the research with regards to getting profits is catered for. If it’s not, then it should be explained legally. Also, the laws that govern these researches may differ because I think these researches are done across multiple countries. So legally maybe what applies here may not apply in another country where these samples are usually going to be analysed …. if you collect DNA samples here according to the laws of this country, and then you take them to the USA, do the laws still apply? Has the person (participant) consented to something that protects them legally from both sides, here and the other side? (Researcher 02, Male).

The same researcher suggested that, for better leverage, it is very important for local researchers to have an idea about the laws of the collaborators’ countries when negotiating contractual agreements.

We talked about the laws of the country; you will find that the laws that govern that sample on the other side are totally different, and they do not consider you. You find that the people on that side consider this as a donation, so the laws that govern these countries dictate a lot. So, you need to know these people before you send them samples (Researcher 02, Male).

### 4.6 Theme 4: Equity and fairness in sharing the benefits of genomic research

Four research ethics committee members expressed a desire for collaborative partnerships in GBR that is characterized by mutual respect and equity. Leaning on past experiences, they were concerned that just like in other studies for which new products were developed, products from GBR studies could also end up being inaccessible to persons from communities/countries that contributed to the insights that informed the development of such products, thereby impacting equity issues in benefit sharing.

When we first started doing the interventional studies in HIV/AIDS a lot of research was taking place in the developing world but when it came to benefits most of the benefits were not reachable to the individuals in the developing countries. So, I can almost imagine such a scenario in this kind of world, where a lot of genetic research is taking place in developing countries but when the remedies, and cures from such research come out they are almost not accessible to the people in developing countries. So those are some of the risks and challenges that are brought about by genetics research (REC member 03, Male).

Two of the four community representatives who participated in this study noted that most research communities are poor and vulnerable, as such, they are usually not considered during benefit sharing negotiations and often miss out on the benefits of GBR. This was attributed to the inequalities and power imbalances between collaborating partners, where institutions from the developing world lack the requisite bargaining power to protect their interests.

I think the biggest challenge is the feeling of lack of control on the part of the investigators where they think that they have no control in what should be done or much say on how the samples should be handled after someone has paid for the research. But once we empower the investigators to know that they are equal partners in this investigation and therefore their rights should be defended very well while negotiating (REC Member 18 Male).

One REC member felt that benefit sharing was an important issue that had been ignored for so long and that this had led to exploitation of researchers, institutions, and communities in the developing world. He suggested that, moving forward, issues concerning benefit sharing should be taken more seriously by local RECs and other stakeholders.

### 4.7 Theme 5: Perceived barriers to fair benefit sharing

Respondents highlighted several perceived barriers and challenges to fair benefit sharing in international collaborative research. Most prominent was the questionable research practices by some foreign collaborators as stated by a REC member:

But here again it depends on the integrity of the researcher because if the researcher has integrity, chances are that they are going to be collaborating with people who have integrity. But as a REC or as a regulator, we should never take this for granted. We should be able to look at the material transfer agreement and look at the clauses that burr third parties from misusing these samples (REC member 17, Male).

Another challenge was the lack of effective communication between collaborating partners. Respondents emphasized the importance of keeping communication channels open throughout the entire research process. One respondent gave an example of a situation where all communication channels were cut off upon the receipt of the biological samples.

So that’s why it goes back to the integrity. Because we have heard of stories where some researchers after getting the samples, you can no longer get their contacts again. If your only contact was by email or phone, if you call, the phone is off forever…. I do not know if in our country there is a law at that moment which protects …. material transferred from Uganda by this researcher (REC 13, Female).

They further added that researchers from developing countries are often denied access to shared samples and data, as such, they miss out on future research opportunities. Respondents also indicated that disrespect and violation of the provisions of MTAs and other collaborative agreements were rife. As indicated by four respondents, these questionable research practices cultivate distrust and are unhealthy for collaborative research.

….. massive tissue was exported, and in the beginning, they were being exported for one thing, but subsequently they have been analyzed and research papers have been published. ….. the first form of annoyance is that there is no Ugandan attached to it as if they did not contribute to this at all. …. the third is there is often no direct benefit to the society in Uganda, to the institution or even to the people who collected this information and right now there is no protection (Researcher 07, Female).

Some respondents felt that the oversight function of UNCST during MTA implementation was limited. They also observed that the role of the RECs in the development and execution of MTAs was similarly limited. One researcher indicated that it is the responsibility of the local provider institution and UNCST to track the use of exported samples and ensure that the terms and conditions of the MTA are respected.

I think, the responsibility should rely on both [provider institution and UNCST]. There should be a level of inter- institutional follow up but also the [national] regulatory body should play a role too (Researcher 11, Male).

Some respondents surmised that these challenges are partly fueled by the lack of effective mechanisms of tracking and monitoring the use of exported biological samples by local research regulators.

### 4.8 Theme 6: The way forward: Fostering fair and equitable benefit sharing in GBR

Overall, several respondents felt that the ground was not leveled and there was neither equity nor fairness in sharing of GBR benefits in collaborative research. They proposed three recommendations for fostering equity and fairness in collaborative GBR between developing and developed countries.

First, some respondents suggested that investigators from the developing world should be empowered with the knowledge and bargaining skills to confidently negotiate MTAs and other collaborative agreements to protect their interests.

I think the MTAs would be the first place to go because that’s where you negotiate the sample transfer, who is receiving it, for what purpose, is there going to be a back shipment in case it’s required? So, the MTAs if we can strengthen that and empower the investigators to confidently negotiate the MTAs to their advantage, I think that will be a starting point (REC Member 18, Male).

Another researcher suggested that institutions should have committees mandated with the responsibility of examining MTAs as stated below:

We could have a small group of people yes to actually look at MTAs. Yes, it will expedite things, you know you can look at an MTA and say why are you taking urine? (Researcher 03, Female).

Second, respondents (three) recommended the strengthening of RECs and extending their mandate to oversee the negotiation and execution of MTAs. They asserted that there is a need for better capacitation of RECs if they are to be successful in ensuring that the provisions of the MTAs are respected by all parties.

It is not only that, but some MTAs we have looked at as a REC have new parties involved that were not part of the original protocol. Then when you ask for a contract or an agreement between the researcher and the other party, they are not there. That is why I said that the clearance should be at the national council (Uganda National Council for Science and Technology) but the implementation and monitoring (should) come back (to the REC) because for us we have the protocol, we know who is involved in the study right from the beginning (REC member 09, Male).

Third, encouraging community engagement and meaningful involvement of communities in MTA negotiations, particularly regarding sharing of the benefits of research. Both researchers and REC members concurred that communities stand to benefit more from GBR than individual participants. All community representatives (four) who participated in this study asserted that in line with the principles of reciprocity and solidarity, communities should actively be involved in benefit sharing negotiations.

I personally think that communities should directly benefit from the benefits of genomic research. Study communities should be part of the patents in genomic research, and I think negotiations should involve them right from the beginning up to the end. We know that genomic research is beneficial, we know that there will be a lot of intellectual properties and we believe that because the genes belong to these people, they should be respected in the sharing of these (benefits) (REC Member 02, Male).

## 5 Discussion

We set out to explore the perceptions of researchers and research ethics committee members on benefit sharing in international collaborative GBR. Our findings show that respondents expressed consistent views on what constitutes benefit sharing in GBR. They opined that the benefits of GBR should be fairly and equitably shared with local researchers, institutions, individual participants, and research communities. They indicated that research communities should actively be involved in benefit sharing negotiations; and that research participants should be informed about benefit sharing arrangements during the informed consent process. Material transfer agreements were considered central in benefit sharing negotiations. However, respondents highlighted several perceived barriers to fair sharing of GBR benefits with host researchers and communities and these include, unfavorable power disparities, limited bargaining power, weak research regulatory systems, and lack of scientific integrity. Recommendations for fostering fair and equitable benefit sharing in GBR were proposed.

Researchers and REC members expressed consistent views on what constitutes benefit sharing in GBR. They also concurred that communities stand to benefit more from GBR than individual participants. Their views on the forms of benefits in GBR are consistent with Munung and de Vries (Munung and de Vries, 2020) and the Framework for Best Practice for Genomics Research and biobanking in Africa (Yakubu et al., 2018; H3Africa Ethics Working Group, 2017). It is important to note that research benefits may not necessarily be tangible. The benefits of GBR are poorly defined, indirect, or realizable over the long term (James et al., 2014). Whereas sharing of intangible benefits such as improvement in health service provision and capacity building may be obvious, the sharing of monetary profits is not so straightforward. Two respondents in this study were uncertain about how the benefits of GBR should be shared with research participants and research communities. Challenges with practical implementation of benefit sharing in GBR have similarly been raised by other authors (Moodley and Beyer, 2019; Munung and de Vries, 2020; Bedeker et al., 2022); the main concern being the intricacies involved in identifying which population or community will share in the financial benefits. This reiterates the importance of genuinely involving communities in discussions on the appropriate forms of benefits (Ndebele and Musesengwa, 2008; Munung and de Vries, 2020). This is a view that was expressed by both researchers and REC members in this study.

### 5.1 Equity and fairness in sharing the benefits of GBR

Overall, most of the views expressed by both researchers and REC members in this study were consistent. However, there were areas that each stakeholder emphasised. Researchers had several trust issues and were afraid of losing control of exported biological samples/data. They believed that partners from developed countries are entitled to most benefits of GBR, especially the monetary ones, because they fund most research in the developing world. On the other hand, REC members expressed more interest in building collaborative partnerships and fair sharing of research benefits with local stakeholders, especially local communities. Emanuel, Wendler (Emanuel Ezekiel et al., 2004) benchmarks of ethical research recommend the building of collaborative partnerships where there is mutual respect, minimization of disparities, and fair and equitable sharing of the benefits of the research (Emanuel Ezekiel et al., 2004). However, this has not been the case in many international collaborative studies (Wynberg and Chennells, 2009; Sathar et al., 2014; Saxena and Gomes, 2016; Freudenthal, 2019). To illustrate this ethical concern better, let us consider the highly publicized dispute between a South African University and the Wellcome Sanger Institute over the alleged commercialization of transferred human genetic material and data originally collected and consented for research purposes only (Blakeley, 2019; Njilo, 2019; Moodley and Kleinsmidt, 2020; Singh and Moodley, 2021). The genetic material were transferred from South Africa to an institution in the United States and later to Wellcome Sanger institute for sequencing as part of a MTA. However, the genetic material were further transferred to a third party for the manufacture of a gene chip without the knowledge of South African University nor with the consent of the sample donors. The South African University further stated that there was no data sharing agreement with the third party and demanded for the repatriation of the genetic material from the Wellcome Trust Sanger institute (Moodley and Kleinsmidt, 2020; Singh and Moodley, 2021). This issue raised a lot of concern and incited debate within the H3Africa Consortium that culminated in developing of several guidance documents (Chadwick et al., 2021; H3Africa, 2017a; H3Africa, 2017b; H3Africa, 2018; H3AfricaConsortium, 2018).

One REC member pointed out benefit sharing benefit sharing was an issue that had been either ignored or neglected by research regulators and contended that this had led to the exploitation of vulnerable communities and researchers/institutions. This finding is consistent with Munung and de Vries (Munung and de Vries, 2020) who reported little awareness of benefit sharing among researchers. Therefore there is a need for open discussion and awaress-building on benefit sharing in international health research. Benefit sharing should also be seriously considered by local RECs and other stakeholders.

### 5.2 Perceived barriers to fair benefit sharing

This study has highlighted a number of perceived barriers towards benefit sharing in international health research. Both researchers and REC members concurred that there are regulatory weaknesses that are negatively impacting GBR. They pointed out that the Ugandan ethics guidelines are vague and do not provide adequate guidance on several aspects of GBR, particularly benefit sharing. Concerns about the appropriateness of ethical and legal frameworks for GBR have also been well documented (Chanda-Kapata et al., 2015; de Vries et al., 2017; Thaldar et al., 2020; Mahomed, 2021; Mahomed and Staunton, 2021). For instance, there is no specific law regulating human genetics and genomic research in Uganda. However, it is important to note that there are national guidelines for accessing genetic resources and benefit sharing (ICT Policy Africa, 2007) that were developed in accordance with the National Environment (Access to Genetic Resources and Benefit Sharing) Regulations, 2005 (Uganda Government, 2005). But, these regulations were established for plant and animal resources. Section 4 (Tishkoff et al., 2009) (d) of this regulation explicitly states that the regulation does not apply to human genetic resources. We thus recommend that, in the absence of specific local guidance on benefit sharing in GBR, researchers and research institutions should refer to the various H3Africa guidelines for guidance (H3Africa Ethics Working Group, 2017; H3Africa, 2017a; H3Africa, 2018; H3Africa, 2020a; H3Africa, 2020b; H3Africa, 2020c).

Respondents, especially REC members, felt that the ground was not levelled and there was neither equity nor fairness in the sharing of GBR benefits in international collaborative research. This was attributed to inequalities and power imbalances between developing and developed country partners, lack of effective communication, and lack of scientific integrity and questionable research practices. All these challenges are well documented in the literature (Corbin et al., 2013; Katisi et al., 2016; Parker and Kingori, 2016; Walsh et al., 2016) and could result in loss of confidence and trust in collaborative research (Moodley and Singh, 2016; Kretser et al., 2019; Coutellec, 2020; Singh et al., 2022). Trustworthiness is key to the success of biomedical research (Kretser et al., 2019; Coutellec, 2020). Some researchers reported that they had at one stage encountered scientific misconduct in international collaborative research, and may be, that is why they did not trust that the provisions of the MTAs would be respected once samples are transferred across national boundaries. Distrust in GBR has been associated with inconsistency in defining ownership and custodianship of biological materials and data (Singh et al., 2022), and poor community engagement (Tindana et al., 2015; Tindana et al., 2017a). We think these sentiments and suspicions might persist with the increasingly mandatory requirement to deposit genomic datasets in public data repositories. Scientists have fears of loss of control over the future use of biological samples and data (Kaye et al., 2015), reduced or no access to translational benefits (Critchley et al., 2017) and reduced willingness of people to participate in research and donate samples. Researchers in developing countries have expressed desire for equitable research partnerships where there is mutual respect and shared decision making (Rosenbloom et al., 2017; Yakubu et al., 2020). We believe this is achievable if researchers, research institutions and funders/sponsors adhere to the principles and responsibilities as recommended by the Singapore (Singapore Statement, 2010) and Montreal (WCRIF, 2013) statements on research integrity respectively. Moving forward, ways should be found to address these challenges and optimise research collaborations into equitable partnerships.

### 5.3 Legal implications and the role of MTAs in benefit sharing

All respondents appreciated the importance of MTAs in GBR and believed that they are key in the transfer of biological samples and associated data. A MTA is a legally enforceable contract that serves the purpose of facilitating the exchange of biological samples and associated data between institutions, as well as safeguarding the interests of sample donors, researchers, and institutions. A MTA also provides guidance on specific ethical and legal principles that must be adhered to when transferring samples (Bubela et al., 2015; Dhai et al., 2019). A MTA should be comprehensive, fair to all parties and should promote equity but, this might not always be the case. The Uganda national ethics guidelines have a detailed section on MTAs and the transfer of biological materials (UNCST, 2014), and UNCST is in the process of developing a standard MTA template. However some respondents felt these guidelines are not robust enough. Several authors have complained about discrepancy in governance processes for GBR, inadequate approaches to benefit sharing (Singh and Moodley, 2021) and the inadequacy of MTAs in sub-Saharan Africa (Sathar et al., 2014; Moodley and Singh, 2016). For example, a 2020 paper published by Thaldar, Botes (Thaldar et al., 2020) reported several weaknesses in the South African (SA) standard MTA that are not consistent with existing laws. The authors contended that the SA MTA does not provide sufficient protection for local institutions and research participants, and is vague on benefit sharing. The SA MTA states that benefit sharing should be “discussed and negotiated’ before any material are transferred, meaning that reaching an agreement on benefit sharing may not be a mandatory requirement (Thaldar et al., 2020). Further, there are reports from several African countries of sample storage and export without informed consent, ethical approval and MTAs (Langat, 2005; Sathar et al., 2014; Ochieng et al., 2022). Such regulatory weaknesses may be exploited by researchers and should therefore be addressed.

### 5.4 Recommendations for fostering fair and equitable benefit sharing in GBR

Respondents proposed three recommendations for fostering fair and equitable sharing in GBR:

#### 5.4.1 Empowering LMIC researchers and institutions

Our findings suggest that there are genuine fears among stakeholders of GBR, and these have primarily been shaped by past experiences of exploitation of African researchers (Rosenbloom et al., 2017). Additionally, research scientists may lack the knowledge and legal authority to negotiate and enter contractual agreements on behalf of their institutions. Respondents therefore recommended capacity strengthening to equip local researchers with necessary confidence and bargaining skills to efficiently negotiate MTAs and other related collaborative agreements. We recommend that institutions establish dedicated departments with legal expertise to negotiate, draft and execute MTAs and other collaborative agreements (Streitz and Bennett, 2003; Rodriguez, 2005).

#### 5.4.2 Strengthening RECs and extending their mandate in MTA development and implementation

Another recommendation proposed by respondents in this study is the strengthening of RECs and extending their mandate to reviewing, approving, and monitoring the execution of MTAs. The intention of this suggestion is beneficial because it seeks to promote best practices, but we feel that it is untenable. First, such an activity would require a lot of resources, yet most RECs in Uganda are poorly resourced. Second, UNCST and most research/academic institutions have no established mechanisms of tracking and monitoring GBR implementation, and the execution of MTAs outside national borders. Third, sample exports in the country are not well streamlined. We therefore posit that extending the mandate of RECs to monitor GBR across national boundaries, including ensuring equity and fairness, requires careful strategic planning and should be done in a phased manner. This is an issue that needs further research.

Our results also suggest that researchers and REC members feel that the national ethics guidelines are not clear on the operationalisation of benefit sharing. For better guidance, we recommend the Framework for Best Practice for Genomics Research and Biobanking in Africa that was developed under the auspices of the H3Africa initiative (Yakubu et al., 2018; H3Africa Ethics Working Group, 2017). The framework is premised on four core principles which emphasize the need for research to be: sensitve and respectful to African value and culture; benefit African people as well as the global population; ensure genuine and active intellectual participation of African stakeholders in research and research dissemination; and promote relationships characterised by respect for individuals and communities, fairness, equity and reciprocity. Regarding benefit sharing, the framework recognises that benefits of GBR may be tangible or intangible. Among others, the framework proposes negotiation of a benefit sharing agreement with relevant stakeholders, including research communities; clearly articulating the potential benefits associated with the research; and how participants and their communities are likely to share in such benefits. The framework further emphasises the need to ensure that no unrealistic expectations are raised, especially about tangible benefits. We believe that these guidelines are appropriate, context specific and were developed with cognizance of the challenges to global health research in sub-Saharan Africa.

#### 5.4.3 Strengthening community engagement

Two respondents in this study were uncertain about how the benefits of GBR should be shared with research participants and research communities. Practical implementation of benefit sharing in GBR has similarly been raised by other authors (Moodley and Beyer, 2019; Munung and de Vries, 2020; Bedeker et al., 2022); the main concern being the intricacies involved in identifying the population or community that will share in the financial benefits. One way of operationalising benefit sharing is through community engagement where communities that stand to benefit from specific research endeavors should agree on the forms of benefits (Ndebele and Musesengwa, 2008; Munung and de Vries, 2020). This is a view that was expressed by both stakeholders in this study.

Both researchers and REC members suggested involvement of research communities in MTA and benefit sharing discussions; and we believe that this can be achieved through community engagement. We therefore recommend strengthening of community engagement in GBR and encourage researchers to meaningfully involve communities in MTA development and benefit sharing negotiations. Based on the principle of justice, communities should be empowered to negotiate benefit sharing early in the community engagement process (Moodley and Beyer, 2019). Community engagement is key in building equitable research collaborations and its value in GBR is increasingly being appreciated (Moodley and Singh, 2016; Tindana et al., 2017b; Atutornu et al., 2022). Community engagement is vital in seeking the views of the community during the design and implementation of research, and for putting forth the interests of the community (Jao et al., 2015). Community engagement also provides a platform for negotiations and establishing community expectations and preferences (Tindana et al., 2017b; Mwaka et al., 2021). This is important because community perception of benefits may differ from the perspective of researchers and other stakeholders. For example, a study among the Maori of Newzealand, recommended that biobanks should ensure that biological samples obtained from the Maori should only be used for projects that directly benefit the Maori communities (James et al., 2014). According to our respondents, information about benefit sharing should be discussed with individual participants during the informed consent process, including any plans of sharing samples/data with commercial entities. This is important because communities may have varying interpretation of commercialization. Some may understand it as the selling of samples and data for profit akin to exploitation, while at the other extreme it may be viewed as a necessity and important for the development of therapeutic interventions for the public good. Such interpretation may impact on the confidence and trust communities have in GBR. Community engagement is therefore important in building trust in GBR. It could also enhance understanding of complex genetic information through community education and sensitization and provide a two-way communication channel through which researchers gain better understanding of community priorities, traditions, practices, and cultural sensitivities.

### 5.5 Limitations and strengths

The main strength of this study was the participation of both researchers and REC members with experience in the conduct and regulation of GBR respectively. This helped us have a multi-dimensional look at a hitherto ignored topic of discussion in Uganda.

The main weakness of the study was the recruitment of researchers from a single institution (MakCHS). However, we believe their views are representative of the wider scientific community in Uganda because of the impact MakCHS has on academia and the research enterprise in Uganda and the East African region. Additionally, any other institution engaging in genetic research would likely face similar contexts as at MakCHS. Lastly, the study did not include participants/communities that were participating in GBR, as such, their insights were not captured. We believe their insights would have added more depth to findings.

## 6 Conclusion

Researchers and REC members expressed various perspectives on benefit sharing in GBR. Although their views on what constitutes benefit sharing in GBR were largely consistent, respondents highlighted various forms of the benefits of GBR as well as barriers to fair and equitable benefit sharing in GBR in North-South collaborative studies. Participants called for fair and equitable sharing of benefits with local researchers, institutions, individual participants, and research communities, including fairness in MTA negotiations; the active involvement of communities in benefit sharing negotiations and for the disclosure of benefit sharing arrangements to research participants during the informed consent process. In order to ensure fair and equitable benefit sharing in North-South collaborative GBR, we advocate for the following measures: strengthening regulatory systems, expanding the role of REC in MTA development and implementation, and meaningful participation of local research communities in benefit sharing negotiations. Research scientists in developing nations should also have their capacities strengthened and empowered so they have the skills necessary to advocate favourably for their institutions’ interests.

## Data Availability

The datasets presented in this study can be found in online repositories. The names of the repository/repositories and accession number(s) can be found in the article/Supplementary Material. The data is publicly available at https://doi.org/10.6084/m9.figshare.20937571.v1.
