# Supplementary material for: Benefit sharing in genomic and biobanking research in Uganda: Perceptions of researchers and research ethics committee members
Source: Front Genet. 2022 Nov 17;13:1037401. doi: 10.3389/fgene.2022.1037401 (PMC9714451; doi:10.3389/fgene.2022.1037401)
Supplement: Supplementary file 1 [file Table1.DOCX]

| Coding Summary By Code | | | | | | | | | | | | | | | | | |
| --- | --- | --- | --- | --- | --- | --- | --- | --- | --- | --- | --- | --- | --- | --- | --- | --- | --- |
| Researchers’ perceptions on biological sample storage, ownership and benefit sharing | | | | | | | | | | | | | | | | | |
|  | | | | | | | | | | | | | | | | | |
|  | | | **Aggregate** |  | **Classification** |  | **Coverage** |  | **Number Of Coding References** | |  | **Reference Number** |  | **Coded By Initials** |  | **Modified On** |  |
| **Node** | | | | | | | | | | | | | | | | |  |
|  | **Nodes\\Conditions for sample storage** | | | | | | | | | | | | | | | |  |
|  | | **Document** | | | | | | | | | | | | | | |  |
|  | | | **Files\\male\\1DI R01 Male** | | | | | | | | | | | | | |  |
|  |  |  | Yes |  |  |  | 0.0245 |  | 1 | |  | | | | | |  |
|  | | |  |  |  |  |  |  |  | |  | | | | | | |
|  | : First of all, over the years I have learnt the passionate, it’s a ahh I have grown passion around the documentation of each sample but also the conditions of storage matter, I know someone (I: that’s very important) someone who told me that they stored samples but can’t use them because they got spoilt, because it was a storage problem and someone (laughs) breached the ethical violation because he told the patient, we store samples and use them. Isn’t it a violation of some sort? But if the onus is on you, if you tell an individual that we shall store the samples until they are used up. To then: 1 document, 2 archive, 3 store them in the right conditions, to enable the use that you are applying them for. This means therefore that you have to get specialized places that can handle this sort of storage for you (I: like bio banks) yes bio banks I think that’s ideal. Initially most of us do our samples and store them with the labs (I: hmm) but usually I see that as a temporary storage during the collection and first initial documentation where we collect them in boxes thereafter it should go to a place which we can document stage where we electronically correct errors in boxes and there after it should go then to a place where you can document and then retrieve the details of the patients. | | | | | | | | | | | | | | | |  |
|  |  |  |  |  |  |  |  |  |  |  |  |  |  |  |  |  |  |
|  |  | | | | | | | | | | | | | | | |  |
|  | **Nodes\\Data;sample sharing** | | | | | | | | | | | | | | | |  |
|  | | **Document** | | | | | | | | | | | | | | |  |
|  | | | **Files\\Female\\1DI R03 female** | | | | | | | | | | | | | |  |
|  |  |  | No |  | person |  | 0.0382 |  | 3 | |  | | | | | |  |
|  | | |  |  |  |  |  |  |  | |  | | | | | | |
|  | R: Samples, I am really not a supporter of samples (I: sample sharing) yes sample sharing unless if it’s a test that cannot absolutely be done here. But if its a test that can be done here or a machine can be imported and the test done here expertise should be developed here (I: yes). | | | | | | | | | | | | | | | |  |
|  |  | | | | | | | | | | | | | | | |  |
|  | R: I mean we have drowns that can be let off in the US to bomb somebody in Afghanistan (I: yes). Surely, we have the technology to bring somebody here down and analyse the sample (I: and teach people) and teach people how to do it because the cost and the (I: hmm) I mean there are samples we have shipped before and there was something at the airport there was something at a European airport and all the samples died (I: okay). At what cost? To the researcher (I: researcher). | | | | | | | | | | | | | | | |  |
|  |  | | | | | | | | | | | | | | | |  |
| Reports\\Coding Summary By Code Report | | | | | | | | | | Page 1 of 40 | | | | | | | |
|  | | | **Aggregate** |  | **Classification** |  | **Coverage** |  | **Number Of Coding References** | |  | **Reference Number** |  | **Coded By Initials** |  | **Modified On** |  |
|  | | | | | | | | | | | | | | | | | |
|  | R: Ahh that particular example (I: yes) we have actually had participants express so we got that hair, that hair thing we dropped but in terms of we have to understand that (I: they had opportunity I am really not comfortable) yeah hair and nails no. so that’s why we dropped that particular study (I: yes). Hair and nails we did not do ahh but usually the participants who come here, come here because they are sick (I: yes) and they are offered treatment as part of the research what? Study, so I think some of their motivation for participation may be to get treatment (phone rings) and of course to get treatment and follow up (I: yes) that’s why we follow them up. and when we tell them genomic research although we offer them at various points during the consent and during the study period their chances to you know talk about the consent form again and see if they still understand it (I: hmm). I think some of them might be like why I am getting treatment I have received treatment and I am better and this is something really abstract in their minds. I don’t know that they really ahh (I: okay). | | | | | | | | | | | | | | | |  |
|  |  |  |  |  |  |  |  |  |  |  |  |  |  |  |  |  |  |
|  |  | | | | | | | | | | | | | | | |  |
|  | | | **Files\\Female\\IDI R15 female** | | | | | | | | | | | | | |  |
|  |  |  | No |  |  |  | 0.0045 |  | 1 | |  | | | | | |  |
|  | | |  |  |  |  |  |  |  | |  | | | | | | |
|  | R: Ohh, to participants themselves, (I: or like you tell them that we have our collaborators will share with them at a certain point) what are the consequences? | | | | | | | | | | | | | | | |  |
|  |  | | | | | | | | | | | | | | | |  |
|  | | | **Files\\male\\1DI R04 Male** | | | | | | | | | | | | | |  |
|  |  |  | No |  |  |  | 0.0095 |  | 1 | |  | | | | | |  |
|  | | |  |  |  |  |  |  |  | |  | | | | | | |
|  | R: So in the first consent if I didn’t know this is an additional test to be done by Case western, (I: not that case western at one day will have collaboration with UCSF) alright, then I would advocate to get back to the patient, modify or reconsent the patient for the new procedure I want to do on the samples because our agreement was not covering extension to UCSF, okay? (I: Yeah) So I will go back to the patient. | | | | | | | | | | | | | | | |  |
|  |  | | | | | | | | | | | | | | | |  |
|  | | | **Files\\male\\IDI R07 male** | | | | | | | | | | | | | |  |
|  |  |  | No |  |  |  | 0.0397 |  | 1 | |  | | | | | |  |
|  | | |  |  |  |  |  |  |  | |  | | | | | | |
|  | I: Now, the participant or the donor you know retains but now when it comes for example if there are some benefits for example patentable information and innovation, should they have a share?  R: They should and I don’t know what is in our policies. Like the CAPRISA people did for that lady who they got an antibody from, should a Ugandan buy a patentable product from a biological sample? To me the benefits should go back to that person, that person where the sample came from should also be included. The policy should be revised accordingly if it doesn’t clearly stipulate those issues.  I: But we have read ahh you researchers we’ve read some of your consent forms; you put that the participants shouldn’t expect any commercial gain.  R: You know, you need to revise those aspects and those should come in a separate section that, “Should anything of value come out of your sample then you will be rewarded,” I think that revision is necessary although by and large the way science is, 90% of the time there is nothing which can come out. | | | | | | | | | | | | | | | |  |
|  |  |  |  |  |  |  |  |  |  |  |  |  |  |  |  |  |  |
|  |  | | | | | | | | | | | | | | | |  |
|  | | | | | | | | | | | | | | | | | |
|  | | | | | | | | | | | | | | | | | |
| Reports\\Coding Summary By Code Report | | | | | | | | | | Page 2 of 40 | | | | | | | |
|  | | | **Aggregate** |  | **Classification** |  | **Coverage** |  | **Number Of Coding References** | |  | **Reference Number** |  | **Coded By Initials** |  | **Modified On** |  |
|  | **Nodes\\Data;sample sharing\How to discuss consequences of sharing** | | | | | | | | | | | | | | | |  |
|  | | **Document** | | | | | | | | | | | | | | |  |
|  | | | **Files\\Female\\IDI R14 female** | | | | | | | | | | | | | |  |
|  |  |  | Yes |  |  |  | 0.0203 |  | 1 | |  | | | | | |  |
|  | | |  |  |  |  |  |  |  | |  | | | | | | |
|  | R: Ahh one I think is assuring them (I: hmm) that much as we are exporting your samples, you are really safe. Like your identity is going to be kept confidential (I: hmm) and because they have this fear of you know white men doing something and you know maybe developing drugs that will maybe kill them. They have those concepts, so I think it is assuring them of the safety of their samples, the future repercussions of sharing data with foreign investigators (I: hmm). Then maybe you can assure them of the credibility of those international investigators that your sharing samples with (I: hmm okay thank you). | | | | | | | | | | | | | | | |  |
|  |  | | | | | | | | | | | | | | | |  |
|  | | | **Files\\Female\\IDI R15 female** | | | | | | | | | | | | | |  |
|  |  |  | Yes |  |  |  | 0.0149 |  | 1 | |  | | | | | |  |
|  | | |  |  |  |  |  |  |  | |  | | | | | | |
|  | R: I think that should be in the consenting process, I mean if you blow, if you give, if you put a separate consent for shipping, you know when you draw attention to one thing, (I: hmm) you magnify it beyond, it becomes like you know (I: bigger than it should be) yes. So I think it should be in the consenting process, if you give permission put it in there, you know that you should ship put it in the consenting process and inform the participants so that they know that their samples when they are stored, they will be shipped. | | | | | | | | | | | | | | | |  |
|  |  | | | | | | | | | | | | | | | |  |
|  | | | **Files\\male\\1DI R04 Male** | | | | | | | | | | | | | |  |
|  |  |  | Yes |  |  |  | 0.0185 |  | 1 | |  | | | | | |  |
|  | | |  |  |  |  |  |  |  | |  | | | | | | |
|  | R: Exactly, I think the most important thing is the original consent whereby you have to include clauses like in case we plan any future testing other than what is stipulated or listed here in the consent we will get back to you or your immediate relatives and we should be able to know, immediate relatives of those patients in case they passed on. Okay because these are sick people for example, we are taking samples from, and you are not sure about their fate in the next ten years, then they should give us those relatives those they feel are the immediate relatives that they feel they have a right to decide on their samples okay then we get back to those people who can consent on their behalf. As long as they sign on that and the clause is in within the consent form, then we can do that (I: hmm) yeah. | | | | | | | | | | | | | | | |  |
|  |  | | | | | | | | | | | | | | | |  |
|  | | | **Files\\male\\1DI R12 Male** | | | | | | | | | | | | | |  |
|  |  |  | Yes |  | person |  | 0.0115 |  | 1 | |  | | | | | |  |
|  | | |  |  |  |  |  |  |  | |  | | | | | | |
|  | Resp: The best way of explaining the consequences is at the time of informed consent. You explain to them what is going to happen, what the benefits are. You tell them at that point. | | | | | | | | | | | | | | | |  |
|  |  | | | | | | | | | | | | | | | |  |
|  | | | **Files\\male\\IDI R05 male** | | | | | | | | | | | | | |  |
|  |  |  | Yes |  |  |  | 0.0382 |  | 3 | |  | | | | | |  |
|  | | |  |  |  |  |  |  |  | |  | | | | | | |
|  | Respondent 1: Laughs. It is a hard one because really the best way for Erisa would not be the best way for the other one but uh consequences are consequences. | | | | | | | | | | | | | | | |  |
|  |  | | | | | | | | | | | | | | | |  |
| Reports\\Coding Summary By Code Report | | | | | | | | | | Page 3 of 40 | | | | | | | |
|  | | | **Aggregate** |  | **Classification** |  | **Coverage** |  | **Number Of Coding References** | |  | **Reference Number** |  | **Coded By Initials** |  | **Modified On** |  |
|  | | | | | | | | | | | | | | | | | |
|  | Respondent 1: Especially if I am to look at them as negative. We should desist from hiding. If something that is not right may happen then someone better knows it other than hiding it and then later they realize, that uh probably they say you were a liar. So to me I think the best way is telling the truth about what would be the real consequences but then also looking at the good. You know you if the bad dominates the good then usually sciences become bad. Then they are always hard and all that. I am just using that because that Is what most of the people have been on. But if we have the good of the research and capitalize on the good of what it would inform, | | | | | | | | | | | | | | | |  |
|  |  | | | | | | | | | | | | | | | |  |
|  | Respondent 1: Yes to you it would be bad but then look at your children.  Interviewer: Umm.  Respondent 1: Uh look at your uncles and aunties. This is the benefit of it.  Interviewer: Um.  Respondent 1: Then I think that’s the best way we could tell them instead of capitalizing the bad. | | | | | | | | | | | | | | | |  |
|  |  | | | | | | | | | | | | | | | |  |
|  | | | **Files\\male\\IDI R06 male** | | | | | | | | | | | | | |  |
|  |  |  | Yes |  |  |  | 0.0396 |  | 1 | |  | | | | | |  |
|  | | |  |  |  |  |  |  |  | |  | | | | | | |
|  | R: The consequences (I: hmm or the best way to discuss these results) you know I mean for me again as I said its best to if you have a good example of what can go bad (I: hmm). Ahh I think that helps, the remember a few ahh was it 2 years ago ahh people in Mild may (I: hmm) you know somebody had either sold or they had or somebody had had access to patient records (I: hmm) and they had sold those documents to people that wrap (laughs) samosas and chapatis (both laugh) on the street and so and that’s how people found out. There were names on the documents, HIV results (I: hmm) you know there was information that could lead to the participant and I agree that and my question is that whether all those things drive the stigma around HIV maybe I don’t know. But ahh there obviously certain things that medical results are always kept confidential and that goes along with any biomedical research, I think. So, I think by letting staff who are working around the studies understand what the consequences are ahh you could do the same thing in your informed consent. Or even ahh there is the information sheet (I: hmm) but there is also the discussion that you have with the potential participants. In terms of if you are discussing why you have to keep their documentation confidential (I: hmm). I think those are some of the examples, live examples I think would be good to use as an example to drive the point home (I: hmm) as far as the consequences of lack of confidentiality are of breaking confidentiality. | | | | | | | | | | | | | | | |  |
|  |  |  |  |  |  |  |  |  |  |  |  |  |  |  |  |  |  |
|  |  | | | | | | | | | | | | | | | |  |
|  | | | **Files\\male\\IDI R07 male** | | | | | | | | | | | | | |  |
|  |  |  | Yes |  |  |  | 0.0224 |  | 1 | |  | | | | | |  |
|  | | |  |  |  |  |  |  |  | |  | | | | | | |
|  | R: I really don’t have a definite answer regarding that but I would imagine that what we need to do now is to be open with the participants regarding these risks of genetic research. We tell them all the confidentiality whatever we are taking to ensure that none of their information or identity is disclosed. But we need to be frank with them and inform them that this DNA/genetic information in as much as we are coding has capacity to code you, it is like a finger print (I: a finger print) yes. They have to be aware of that and so that they are not shocked in case something happens. | | | | | | | | | | | | | | | |  |
| Reports\\Coding Summary By Code Report | | | | | | | | | | Page 4 of 40 | | | | | | | |
|  | | | **Aggregate** |  | **Classification** |  | **Coverage** |  | **Number Of Coding References** | |  | **Reference Number** |  | **Coded By Initials** |  | **Modified On** |  |
|  | | | **Files\\male\\IDI R08 Male** | | | | | | | | | | | | | |  |
|  |  |  | Yes |  |  |  | 0.0334 |  | 1 | |  | | | | | |  |
|  | | |  |  |  |  |  |  |  | |  | | | | | | |
|  | : Of course, now we have consequences of the you know the sample and data sharing. How best can we discuss the consequences of this to participants? You know like we are taking off your samples, we are going to share them, we are going to export them. Some of them might think that you know we are going to sell them (R: yeah) because they believe there is nothing for free. How best can we you know, you know like the consequences, that what can happen you know when we share their information and data. How best can we discuss this with these participants?  R: Ahh when your giving them information, you need to of course you provide them with the information of what will happen to their samples but I know if you have done something on those samples and you come up with something significant finding which you may think is helpful to somebody there should be a way of communicating to them although we make them anonymous (laughs) in the first place. | | | | | | | | | | | | | | | |  |
|  |  |  |  |  |  |  |  |  |  |  |  |  |  |  |  |  |  |
|  |  | | | | | | | | | | | | | | | |  |
|  | **Nodes\\Export and ownership of samples** | | | | | | | | | | | | | | | |  |
|  | | **Document** | | | | | | | | | | | | | | |  |
|  | | | **Files\\Female\\1DI R03 female** | | | | | | | | | | | | | |  |
|  |  |  | Yes |  | person |  | 0.0034 |  | 1 | |  | | | | | |  |
|  | | |  |  |  |  |  |  |  | |  | | | | | | |
|  | : Okay, do you think they can share the samples but with them retaining the samples here? Share access to the samples  R: The local researchers (I: yes, the foreign) | | | | | | | | | | | | | | | |  |
|  |  | | | | | | | | | | | | | | | |  |
|  | | | **Files\\Female\\IDI R14 female** | | | | | | | | | | | | | |  |
|  |  |  | Yes |  |  |  | 0.0158 |  | 1 | |  | | | | | |  |
|  | | |  |  |  |  |  |  |  | |  | | | | | | |
|  | R: Ahh I think it is good (I: hmm) one because we may not have the capacity to do certain investigations and the capacity may be overseas. And also, collaboration is good, like ahh it brings new ahh (laughs) I don’t know how to say it (I: hmm) like what comes with collaboration, besides the capacity if we don’t have it here. May be with the collaboration, the research can broaden in case something comes (I: different questions come up) exactly. In case different questions come up | | | | | | | | | | | | | | | |  |
|  |  | | | | | | | | | | | | | | | |  |
|  | | | **Files\\Female\\IDI R15 female** | | | | | | | | | | | | | |  |
|  |  |  | Yes |  |  |  | 0.0584 |  | 2 | |  | | | | | |  |
|  | | |  |  |  |  |  |  |  | |  | | | | | | |
|  | R: I think it’s something that has its pros and cons, a lot of times, the samples go out there and they do more ahh you can’t govern what they do. They do more than what you agreed to do, but if they are responsible, institutions with integrity, it can be useful. We have to accept that our capacity is limited and even where we have capacity the costs can be really be high (I: hmm). So, it might be more cost effective to just be ship and analyse them and we can’t deny that many at times that this is collaborative effort so the roles can be dispersed but it’s up to the institute, the sponsor, the PI. They have to ensure that those samples are not misused, from my experience, we always send one sample leave one sample behind. You can’t say that I have no samples for this study, they all are in America, you always keep one behind and budget those costs. It’s a tricky issue because like you said knowledge is power and when they have your samples they will go and do the genotype. They will go and do everything they ever want to do. The day they decide to clone human beings they can clone all of Africa because they have the samples in a bio bank in the US (both laugh). So, it’s quite sensitive but I guess it depends on the integrity of the institute where you are (I: sending them) it’s tricky. | | | | | | | | | | | | | | | |  |
|  |  |  |  |  |  |  |  |  |  |  |  |  |  |  |  |  |  |
|  |  | | | | | | | | | | | | | | | |  |
| Reports\\Coding Summary By Code Report | | | | | | | | | | Page 5 of 40 | | | | | | | |
|  | | | **Aggregate** |  | **Classification** |  | **Coverage** |  | **Number Of Coding References** | |  | **Reference Number** |  | **Coded By Initials** |  | **Modified On** |  |
|  | | | | | | | | | | | | | | | | | |
|  | : So, let’s talk about limited capacity, so of course there are situations where some people ship all their samples and the are left with nothing (R: that’s stupidity) laughs I know. So how, (R: I don’t know who would accept to do something like that) and then they get new students who want to use their samples and stuff like that. (R: that is stupidity) And then they are asking people in the US to send back the samples  R: That’s stupidity, I think we need to be more wise in the way we conduct our research and even in handling our collaborators, the data is from here, the data is owned by the people here. This is a collaborative effort but they need to understand that the data, the samples are from the African population, you can’t as in that is wrong. Its just wrong | | | | | | | | | | | | | | | |  |
|  |  | | | | | | | | | | | | | | | |  |
|  | | | **Files\\male\\IDI R02 Male** | | | | | | | | | | | | | |  |
|  |  |  | Yes |  |  |  | 0.0085 |  | 1 | |  | | | | | |  |
|  | | |  |  |  |  |  |  |  | |  | | | | | | |
|  | | | | | | | | | | | |  |  |  |  |  |  |
|  | Resp: The people need to know each other before that happens. You need to know who is this person that you are working with. That’s the only way out otherwise you have no control once the samples leave you. | | | | | | | | | | | | | | | |  |
|  |  | | | | | | | | | | | | | | | |  |
|  | | | **Files\\male\\IDI R05 male** | | | | | | | | | | | | | |  |
|  |  |  | Yes |  |  |  | 0.0805 |  | 2 | |  | | | | | |  |
|  | | |  |  |  |  |  |  |  | |  | | | | | | |
|  | | | | | | | | | | | |  |  |  |  |  |  |
|  | Respondent 1: Laughs  Respondent 2: Laughs  Respondent 1: I look at it in two ways.  Interviewer: Umm. | | | | | | | | | | | | | | | |  |
|  | | | | | | | | | | | | | | | | | |
|  | | | | | | | | | | | | | | | | | |
| Reports\\Coding Summary By Code Report | | | | | | | | | | Page 6 of 40 | | | | | | | |
|  | | | **Aggregate** |  | **Classification** |  | **Coverage** |  | **Number Of Coding References** | |  | **Reference Number** |  | **Coded By Initials** |  | **Modified On** |  |
|  | | | | | | | | | | | | | | | | | |
|  | Respondent 1: One it is not good. It is bad.  Interviewer: Why?  Respondent 1: Why is it bad is that it limits capacity building here back at home. But then sometimes on the other side it’s good because it’s unavoidable. Probably there are some uh things that we could not do here, some tests that can only be done outside. Actually refusing to allow the export is hindering other findings.  Interviewer: Umm.  Respondent 1: So that is how it would be good. But then on the other hand my debate would be that ok in any case you intend to do something, can we build that capacity, that for which you are exporting it for can’t you bring it back home so that it can be done here (Laughs).  Interviewer: Umm.  Respondent 1: And in that way probably the African setting is also brought up.  Respondent 2: There is a joke around that our forefathers allowed people to be taken in whole.  Respondent 1: Laughs  Respondent 2: Laughs  Interviewer: So.  Respondent 2: And then now for us we are  Interviewer: Just taking as samples.  Respondent 2: So what is the difference between them and now?  Respondent 1: Laughs  Respondent 2: Laughs  Interviewer: That’s a nice one.  Respondent 2: Yes (Laughs).  Interviewer: Ok. So how can the export of these samples be regulated? In your opinion how should they be regulated? The export of samples.  Respondent 1: It is an expensive thing but uh I think it requires us to also be focused to it. Because the only way it can be regulated is if we have all that it takes here.  Interviewer: Umm.  Respondent 1: But it is a very expensive venture.  Interviewer: Umm.  Respondent 1: Uh an example is sequencing. For example how many firms of sequencing do we have around? You have to go to the UVRI. We have a sequencer here.  Interviewer: Umm.  Respondent 1: But when we discuss the costs you find that someone probably who has shipped is investing less than someone who has done it here.  Interviewer: Umm.  Respondent 1: Uh the expensive venture is that I think we need to be deliberate on this. We who are already in the field.  Interviewer: Umm.  Respondent 1: That we try our level best to bring things home so that in that way whoever wants to export, you tell them, but we can do that here.  Interviewer: Umm. | | | | | | | | | | | | | | | |  |
|  |  |  |  |  |  |  |  |  |  |  |  |  |  |  |  |  |  |
|  |  |  |  |  |  |  |  |  |  |  |  |  |  |  |  |  |  |
|  |  |  |  |  |  |  |  |  |  |  |  |  |  |  |  |  |  |
|  |  |  |  |  |  |  |  |  |  |  |  |  |  |  |  |  |  |
|  |  |  |  |  |  |  |  |  |  |  |  |  |  |  |  |  |  |
|  |  | | | | | | | | | | | | | | | |  |
|  | | | **Files\\male\\IDI R07 male** | | | | | | | | | | | | | |  |
|  |  |  | Yes |  |  |  | 0.0442 |  | 1 | |  | | | | | |  |
|  | | |  |  |  |  |  |  |  | |  | | | | | | |
|  | R: My thoughts are, I’m pro export but am against it (I: give reasons for it). Since our science is still hmm, we are not yet fully established in all the realm of Science; be it bio-medical, be it… hmm for clinical, we are relatively ok but consider basic sciences, we are not yet where we would like to be and so at that level it is not impossible to have all the cutting age technology we would want. So that forces an investigator to ship a sample to a western setting where he can go and learn but I only support that if there is a plan for the technology to be transferred at home. So, where I am against shipping samples of course it’s a bad habit if some investigators all they want is to ship samples and then the local researchers are not even rewarded, they are not even authors, they are not even acknowledged. That has been the practice, I think that should stop (I: hmm) in that case. And where the science is established for instance in the clinics or even in simple molecular technologies, there should be no shipping. However costly it may be, let those foreign investigators ensure that more assays are done locally and the cost is reduced. | | | | | | | | | | | | | | | |  |
|  |  |  |  |  |  |  |  |  |  |  |  |  |  |  |  |  |  |
|  |  | | | | | | | | | | | | | | | |  |
|  | | | | | | | | | | | | | | | | | |
| Reports\\Coding Summary By Code Report | | | | | | | | | | Page 7 of 40 | | | | | | | |
|  | | | | | | | | | | | | | | | | | |
|  | | | **Aggregate** |  | **Classification** |  | **Coverage** |  | **Number Of Coding References** | |  | **Reference Number** |  | **Coded By Initials** |  | **Modified On** |  |
|  | | | **Files\\male\\IDI R08 Male** | | | | | | | | | | | | | |  |
|  |  |  | Yes |  |  |  | 0.0440 |  | 1 | |  | | | | | |  |
|  | | |  |  |  |  |  |  |  | |  | | | | | | |
|  | | | | | | | | | | | | 1 |  |  |  |  |  |
|  | I: So, what do you think about sharing samples with foreign collaborators? How best should these samples be shared because I believe you have exported some of these (R: yes). What do you think about this? Just in your opinion  R: Yeah sharing can be done to do different research but I will think its best for us to develop capacity here such that most of the samples are used within because collaborators can come and work here. Capacity is lacking here, they should be encouraged to come and develop capacity here such that instead of us sending the samples to them. They can come and do the research here and, in the process, build further capacity here to do this research either in terms of getting facilities or in terms of training people to do that sort of research. But that said, there may be some procedures which are so specialized that may not be able to be done here. So, in such instances you may be required to share the samples with them. In those exceptional circumstances I think it is okay to share but I don’t think we should generally leave them to take everything even for simple things which we can do. But of course, it may need now deliberate effort to build capacity here so this should be something which happens over time slowly. | | | | | | | | | | | | | | | |  |
|  |  |  |  |  |  |  |  |  |  |  |  |  |  |  |  |  |  |
|  |  | | | | | | | | | | | | | | | |  |
|  | | | **Files\\male\\IDI R09 Male** | | | | | | | | | | | | | |  |
|  |  |  | Yes |  |  |  | 0.0196 |  | 1 | |  | | | | | |  |
|  | | |  |  |  |  |  |  |  | |  | | | | | | |
|  | | | | | | | | | | | | 1 |  |  |  |  |  |
|  | R: Like I have told you from a scientific point of view. Scientists live in a network we collaborate, we enrich each other. So, in one way or another that is unavoidable, it hard to happen. Only that it has to be regulated it is the US regulated, us be regulated. It also has to be minimised by you building capacity and then probably you can do as much and minimise what you want to share. Because if I had sequenced, if I had a good bio informatic capacity like we are having now maybe I don’t need to ship the DNA. I need to ship you the sequence you want (I: hmm) in that case I do more regulation rather than giving you a sample to do what ever you want. So, it is inevitable but it needs to minimise and be regulated, yes. | | | | | | | | | | | | | | | |  |
|  |  | | | | | | | | | | | | | | | |  |
|  | | | **Files\\male\\IDI R10 male** | | | | | | | | | | | | | |  |
|  |  |  | Yes |  |  |  | 0.0031 |  | 1 | |  | | | | | |  |
|  | | |  |  |  |  |  |  |  | |  | | | | | | |
|  | | | | | | | | | | | |  |  |  |  |  |  |
|  | Respondent: Depending on the circumstances. Number one clearly in the past this has been uh exploited and used wrongly. But depending on the circumstances. | | | | | | | | | | | | | | | |  |
|  |  | | | | | | | | | | | | | | | |  |
|  | | | **Files\\male\\IDI R11 male** | | | | | | | | | | | | | |  |
|  |  |  | Yes |  | person |  | 0.0138 |  | 1 | |  | | | | | |  |
|  | | |  |  |  |  |  |  |  | |  | | | | | | |
|  | | | | | | | | | | | |  |  |  |  |  |  |
|  | Interviewer: yeah, of course when you read the national guidelines, the participant is the owner, but the research institutions are the custodians and they are given the rights to use the samples as they so wish as long as the participant consented. ok, what do you think about the export and sharing of samples with international collaborators (of course, this is a question because there has been a lot of abuse of this ample sharing) | | | | | | | | | | | | | | | |  |
|  |  | | | | | | | | | | | | | | | |  |
|  | | | | | | | | | | | | | | | | | |
| Reports\\Coding Summary By Code Report | | | | | | | | | | Page 8 of 40 | | | | | | | |
|  | | | | | | | | | | | | | | | | | |
|  | | | **Aggregate** |  | **Classification** |  | **Coverage** |  | **Number Of Coding References** | |  | **Reference Number** |  | **Coded By Initials** |  | **Modified On** |  |
|  | **Nodes\\Export and ownership of samples\opinion on export** | | | | | | | | | | | | | | | |  |
|  | | **Document** | | | | | | | | | | | | | | |  |
|  | | | **Files\\Female\\IDI R14 female** | | | | | | | | | | | | | |  |
|  |  |  | Yes |  |  |  | 0.0158 |  | 1 | |  | | | | | |  |
|  | | |  |  |  |  |  |  |  | |  | | | | | | |
|  | | | | | | | | | | | | 1 |  |  |  |  |  |
|  | R: Ahh I think it is good (I: hmm) one because we may not have the capacity to do certain investigations and the capacity may be overseas. And also, collaboration is good, like ahh it brings new ahh (laughs) I don’t know how to say it (I: hmm) like what comes with collaboration, besides the capacity if we don’t have it here. May be with the collaboration, the research can broaden in case something comes (I: different questions come up) exactly. In case different questions come up | | | | | | | | | | | | | | | |  |
|  |  | | | | | | | | | | | | | | | |  |
|  | | | **Files\\Female\\IDI R15 female** | | | | | | | | | | | | | |  |
|  |  |  | Yes |  |  |  | 0.0584 |  | 2 | |  | | | | | |  |
|  | | |  |  |  |  |  |  |  | |  | | | | | | |
|  | | | | | | | | | | | | 1 |  |  |  |  |  |
|  | R: I think it’s something that has its pros and cons, a lot of times, the samples go out there and they do more ahh you can’t govern what they do. They do more than what you agreed to do, but if they are responsible, institutions with integrity, it can be useful. We have to accept that our capacity is limited and even where we have capacity the costs can be really be high (I: hmm). So, it might be more cost effective to just be ship and analyse them and we can’t deny that many at times that this is collaborative effort so the roles can be dispersed but it’s up to the institute, the sponsor, the PI. They have to ensure that those samples are not misused, from my experience, we always send one sample leave one sample behind. You can’t say that I have no samples for this study, they all are in America, you always keep one behind and budget those costs. It’s a tricky issue because like you said knowledge is power and when they have your samples they will go and do the genotype. They will go and do everything they ever want to do. The day they decide to clone human beings they can clone all of Africa because they have the samples in a bio bank in the US (both laugh). So, it’s quite sensitive but I guess it depends on the integrity of the institute where you are (I: sending them) it’s tricky. | | | | | | | | | | | | | | | |  |
|  |  |  |  |  |  |  |  |  |  |  |  |  |  |  |  |  |  |
|  |  | | | | | | | | | | | | | | | |  |
|  | | | | | | | | | | | | 2 |  |  |  |  |  |
|  | : So, let’s talk about limited capacity, so of course there are situations where some people ship all their samples and the are left with nothing (R: that’s stupidity) laughs I know. So how, (R: I don’t know who would accept to do something like that) and then they get new students who want to use their samples and stuff like that. (R: that is stupidity) And then they are asking people in the US to send back the samples  R: That’s stupidity, I think we need to be more wise in the way we conduct our research and even in handling our collaborators, the data is from here, the data is owned by the people here. This is a collaborative effort but they need to understand that the data, the samples are from the African population, you can’t as in that is wrong. Its just wrong | | | | | | | | | | | | | | | |  |
|  | | | | | | | | | | | | | | | | | |
| Reports\\Coding Summary By Code Report | | | | | | | | | | Page 9 of 40 | | | | | | | |
|  | | | | | | | | | | | | | | | | | |
|  | | | **Aggregate** |  | **Classification** |  | **Coverage** |  | **Number Of Coding References** | |  | **Reference Number** |  | **Coded By Initials** |  | **Modified On** |  |
|  | | | **Files\\male\\IDI R05 male** | | | | | | | | | | | | | |  |
|  |  |  | Yes |  |  |  | 0.0769 |  | 1 | |  | | | | | |  |
|  | | |  |  |  |  |  |  |  | |  | | | | | | |
|  | | | | | | | | | | | | 1 |  |  |  |  |  |
|  | Respondent 1: One it is not good. It is bad.  Interviewer: Why?  Respondent 1: Why is it bad is that it limits capacity building here back at home. But then sometimes on the other side it’s good because it’s unavoidable. Probably there are some uh things that we could not do here, some tests that can only be done outside. Actually refusing to allow the export is hindering other findings.  Interviewer: Umm.  Respondent 1: So that is how it would be good. But then on the other hand my debate would be that ok in any case you intend to do something, can we build that capacity, that for which you are exporting it for can’t you bring it back home so that it can be done here (Laughs).  Interviewer: Umm.  Respondent 1: And in that way probably the African setting is also brought up.  Respondent 2: There is a joke around that our forefathers allowed people to be taken in whole.  Respondent 1: Laughs  Respondent 2: Laughs  Interviewer: So.  Respondent 2: And then now for us we are  Interviewer: Just taking as samples.  Respondent 2: So what is the difference between them and now?  Respondent 1: Laughs  Respondent 2: Laughs  Interviewer: That’s a nice one.  Respondent 2: Yes (Laughs).  Interviewer: Ok. So how can the export of these samples be regulated? In your opinion how should they be regulated? The export of samples.  Respondent 1: It is an expensive thing but uh I think it requires us to also be focused to it. Because the only way it can be regulated is if we have all that it takes here.  Interviewer: Umm.  Respondent 1: But it is a very expensive venture.  Interviewer: Umm.  Respondent 1: Uh an example is sequencing. For example how many firms of sequencing do we have around? You have to go to the UVRI. We have a sequencer here.  Interviewer: Umm.  Respondent 1: But when we discuss the costs you find that someone probably who has shipped is investing less than someone who has done it here.  Interviewer: Umm.  Respondent 1: Uh the expensive venture is that I think we need to be deliberate on this. We who are already in the field.  Interviewer: Umm.  Respondent 1: That we try our level best to bring things home so that in that way whoever wants to export, you tell them, but we can do that here.  Interviewer: Umm. | | | | | | | | | | | | | | | |  |
|  |  |  |  |  |  |  |  |  |  |  |  |  |  |  |  |  |  |
|  |  |  |  |  |  |  |  |  |  |  |  |  |  |  |  |  |  |
|  |  |  |  |  |  |  |  |  |  |  |  |  |  |  |  |  |  |
|  |  |  |  |  |  |  |  |  |  |  |  |  |  |  |  |  |  |
|  |  |  |  |  |  |  |  |  |  |  |  |  |  |  |  |  |  |
|  |  | | | | | | | | | | | | | | | |  |
|  | | | **Files\\male\\IDI R07 male** | | | | | | | | | | | | | |  |
|  |  |  | Yes |  |  |  | 0.0442 |  | 1 | |  | | | | | |  |
|  | | |  |  |  |  |  |  |  | |  | | | | | | |
|  | | | | | | | | | | | | 1 |  |  |  |  |  |
|  | R: My thoughts are, I’m pro export but am against it (I: give reasons for it). Since our science is still hmm, we are not yet fully established in all the realm of Science; be it bio-medical, be it… hmm for clinical, we are relatively ok but consider basic sciences, we are not yet where we would like to be and so at that level it is not impossible to have all the cutting age technology we would want. So that forces an investigator to ship a sample to a western setting where he can go and learn but I only support that if there is a plan for the technology to be transferred at home. So, where I am against shipping samples of course it’s a bad habit if some investigators all they want is to ship samples and then the local researchers are not even rewarded, they are not even authors, they are not even acknowledged. That has been the practice, I think that should stop (I: hmm) in that case. And where the science is established for instance in the clinics or even in simple molecular technologies, there should be no shipping. However costly it may be, let those foreign investigators ensure that more assays are done locally and the cost is reduced. | | | | | | | | | | | | | | | |  |
|  |  |  |  |  |  |  |  |  |  |  |  |  |  |  |  |  |  |
|  |  | | | | | | | | | | | | | | | |  |
| Reports\\Coding Summary By Code Report | | | | | | | | | | Page 10 of 40 | | | | | | | |
|  | | | | | | | | | | | | | | | | | |
|  | | | **Aggregate** |  | **Classification** |  | **Coverage** |  | **Number Of Coding References** | |  | **Reference Number** |  | **Coded By Initials** |  | **Modified On** |  |
|  | | | **Files\\male\\IDI R08 Male** | | | | | | | | | | | | | |  |
|  |  |  | Yes |  |  |  | 0.0440 |  | 1 | |  | | | | | |  |
|  | | |  |  |  |  |  |  |  | |  | | | | | | |
|  | | | | | | | | | | | | 1 |  |  |  |  |  |
|  | I: So, what do you think about sharing samples with foreign collaborators? How best should these samples be shared because I believe you have exported some of these (R: yes). What do you think about this? Just in your opinion  R: Yeah sharing can be done to do different research but I will think its best for us to develop capacity here such that most of the samples are used within because collaborators can come and work here. Capacity is lacking here, they should be encouraged to come and develop capacity here such that instead of us sending the samples to them. They can come and do the research here and, in the process, build further capacity here to do this research either in terms of getting facilities or in terms of training people to do that sort of research. But that said, there may be some procedures which are so specialized that may not be able to be done here. So, in such instances you may be required to share the samples with them. In those exceptional circumstances I think it is okay to share but I don’t think we should generally leave them to take everything even for simple things which we can do. But of course, it may need now deliberate effort to build capacity here so this should be something which happens over time slowly. | | | | | | | | | | | | | | | |  |
|  |  |  |  |  |  |  |  |  |  |  |  |  |  |  |  |  |  |
|  |  | | | | | | | | | | | | | | | |  |
|  | | | **Files\\male\\IDI R09 Male** | | | | | | | | | | | | | |  |
|  |  |  | Yes |  |  |  | 0.0196 |  | 1 | |  | | | | | |  |
|  | | |  |  |  |  |  |  |  | |  | | | | | | |
|  | | | | | | | | | | | |  |  |  |  |  |  |
|  | R: Like I have told you from a scientific point of view. Scientists live in a network we collaborate, we enrich each other. So, in one way or another that is unavoidable, it hard to happen. Only that it has to be regulated it is the US regulated, us be regulated. It also has to be minimised by you building capacity and then probably you can do as much and minimise what you want to share. Because if I had sequenced, if I had a good bio informatic capacity like we are having now maybe I don’t need to ship the DNA. I need to ship you the sequence you want (I: hmm) in that case I do more regulation rather than giving you a sample to do what ever you want. So, it is inevitable but it needs to minimise and be regulated, yes. | | | | | | | | | | | | | | | |  |
|  |  | | | | | | | | | | | | | | | |  |
|  | | | **Files\\male\\IDI R10 male** | | | | | | | | | | | | | |  |
|  |  |  | Yes |  |  |  | 0.0031 |  | 1 | |  | | | | | |  |
|  | | |  |  |  |  |  |  |  | |  | | | | | | |
|  | | | | | | | | | | | |  |  |  |  |  |  |
|  | Respondent: Depending on the circumstances. Number one clearly in the past this has been uh exploited and used wrongly. But depending on the circumstances. | | | | | | | | | | | | | | | |  |
| Reports\\Coding Summary By Code Report | | | | | | | | | | Page 11 of 40 | | | | | | | |
|  | | | | | | | | | | | | | | | | | |
|  | | | **Aggregate** |  | **Classification** |  | **Coverage** |  | **Number Of Coding References** | |  | **Reference Number** |  | **Coded By Initials** |  | **Modified On** |  |
|  | **Nodes\\Recommendations** | | | | | | | | | | | | | | | |  |
|  | | **Document** | | | | | | | | | | | | | | |  |
|  | | | **Files\\male\\IDI R05 male** | | | | | | | | | | | | | |  |
|  |  |  | Yes |  |  |  | 0.0341 |  | 1 | |  | | | | | |  |
|  | | |  |  |  |  |  |  |  | |  | | | | | | |
|  | | | | | | | | | | | | 1 |  |  |  |  |  |
|  | Respondent 1: So we have a limitation in that case and you people in the IRB can help us to be more stringent (Laughs) and you are like, no these things can be done here. You don’t have to do that.  Interviewer: So the IRB. So it is at several levels. You have the researcher level to build capacity then IRB.  Respondent 1: Ethical Committees.  Interviewer: Yes.  Respondent 2: Even at Government level.  Interviewer: Umm.  Respondent 2: Ultimately we have to develop capacity to do these things.  Interviewer: Umm.  Respondent 2: If there was the capacity to do these things within it would make the work of the IRB simpler.  Respondent 1: Simpler.  Interviewer: Then, tracking and monitoring usage. Because for most researchers will tell you once these samples leave Uganda, we lose control. You I mean you have a bio-repository. Have you put in place you know measures to monitor? You know samples. That are they use for the purposes for which they were collected. Do you have any mechanism? | | | | | | | | | | | | | | | |  |
|  |  |  |  |  |  |  |  |  |  |  |  |  |  |  |  |  |  |
|  |  |  |  |  |  |  |  |  |  |  |  |  |  |  |  |  |  |
|  |  | | | | | | | | | | | | | | | |  |
|  | | | **Files\\male\\IDI R08 Male** | | | | | | | | | | | | | |  |
|  |  |  | Yes |  |  |  | 0.0546 |  | 1 | |  | | | | | |  |
|  | | |  |  |  |  |  |  |  | |  | | | | | | |
|  | | | | | | | | | | | | 1 |  |  |  |  |  |
|  | : So how ahh because like you have already alluded to it. You know like once samples leave here (R: you have no control) we don’t even know what happens to them. So is there, I mean do you have any suggestions of how you know... How can we try you know to track and monitor the usage of these samples? I mean in your own words how would you like it to be done? because we would like to look at the opinions of several ahh the lead researchers so that we can recommend to National council that maybe try this or that. Is there a way we can improve on the monitoring and tracking of this?  R: Laughs, there has to be a way of ensuring that people comply and one of the ways is to ask them to give reports on what they (I: hmm) do. But another definitive way is to see what they have done with it, although it is still possible for somebody to hide (laughs) what they have done with it. So maybe as part of the requirements in future they could require that when samples are shared in such a way resources are provided to enable the regulatory authorities to monitor what happens but working that out again is ahh it involves a lot of things. Or they may put in regulations which require institutions which they have on their side to monitor what the researchers do with those sample then they give reports (I: okay) to the regulatory authorities here (I: okay thanks very much) and the research collaboration. There needs to be some way of monitoring what they do with the samples either through the institutions there or involving the institutions here to monitor. | | | | | | | | | | | | | | | |  |
|  |  |  |  |  |  |  |  |  |  |  |  |  |  |  |  |  |  |
|  |  | | | | | | | | | | | | | | | |  |
|  | | | **Files\\male\\IDI R09 Male** | | | | | | | | | | | | | |  |
|  |  |  | Yes |  |  |  | 0.0485 |  | 3 | |  | | | | | |  |
|  | | |  |  |  |  |  |  |  | |  | | | | | | |
|  | | | | | | | | | | | | 1 |  |  |  |  |  |
|  | I: Of course, now another problem we as regulators of research are having. Once the samples leave this place, are there any mechanisms of tracking the samples?  R: surprisingly yes (I: okay) yes because you see, people can from where ever they come and they mess us up but once they are in their country, they fear ethics and legal issues. They fear to be caught on the wrong side of that ethics so of course if you don’t follow, they have a field day. But if you raised something, they will ask infact many times we have requested to return they will return. If you write and say return may things and account for whatever they will because they know they are ethically obligated. They don’t want to be caught ethically wanting because that is their career. They will be finished. | | | | | | | | | | | | | | | |  |
|  |  |  |  |  |  |  |  |  |  |  |  |  |  |  |  |  |  |
|  |  | | | | | | | | | | | | | | | |  |
| Reports\\Coding Summary By Code Report | | | | | | | | | | Page 12 of 40 | | | | | | | |
|  | | | | | | | | | | | | | | | | | |
|  | | | **Aggregate** |  | **Classification** |  | **Coverage** |  | **Number Of Coding References** | |  | **Reference Number** |  | **Coded By Initials** |  | **Modified On** |  |
|  | | | | | | | | | | | | | | | | | |
|  | | | | | | | | | | | | 2 |  |  |  |  |  |
|  | : So that means our, in our situation it is laxity (R: laxity). Is it laxity or absence of laws and policy?  R: That I think the MTA gives you to do that. Already that is already given in the MTA but are you enforcing it? Do you really even think it is important? Now you realise that if the MTA is not well done you are losing capacity building. Your participants have consented in vain so you but you are there the guardian of this. You should put firm resources to this yeah. | | | | | | | | | | | | | | | |  |
|  |  | | | | | | | | | | | | | | | |  |
|  | | | | | | | | | | | | 3 |  |  |  |  |  |
|  | R: I think we have covered quite a lot but I think what goes on is that this ethics. This is a new area where we need to build capacity for our selves both in ethics and it also goes in capacity building and the capability to handle these things locally and maybe redo regulations and keeping ahh keeping people. Ahh now the bio repository we are ahh still struggling keeping guidelines for the country. And then community engagement, the community needs to be engaged in whatever we do. So those are for me the take home things I can see. | | | | | | | | | | | | | | | |  |
|  |  | | | | | | | | | | | | | | | |  |
|  | | | **Files\\male\\IDI R10 male** | | | | | | | | | | | | | |  |
|  |  |  | Yes |  |  |  | 0.0234 |  | 2 | |  | | | | | |  |
|  | | |  |  |  |  |  |  |  | |  | | | | | | |
|  | | | | | | | | | | | | 1 |  |  |  |  |  |
|  | Respondent: Local capacity in biomedical research is uh no one’s mandate. It is the mandate local governments. The local people need to do this. | | | | | | | | | | | | | | | |  |
|  |  | | | | | | | | | | | | | | | |  |
|  | | | | | | | | | | | | 2 |  |  |  |  |  |
|  | Respondent: On the conditions I think this is very important. The sharing of samples. Research being able to share isolates and other things across the world is very important. I mean I would give you an example uh of cholera which has different strains or flu for instance. Different strains. If flu broke out in Thailand and Thai people said we are not sharing the isolates then Sanofi Pasteur in whatever France will not be able to develop a vaccine because they do not have this particular variant or strain of uh the flu virus. So the sharing of samples is important depending on the circumstances. The conditions, policies you know the reigning environment underwhich everything is done I think is really what defines uh and these are things by the way you need to loo at in depth. So we’ve looked at a big picture of genomics research. But there are things that for instance the shipping of samples. Some one needs to develop clear samples like the Nagoya  Interviewer: The Nagoya Declaration. | | | | | | | | | | | | | | | |  |
|  |  |  |  |  |  |  |  |  |  |  |  |  |  |  |  |  |  |
| Reports\\Coding Summary By Code Report | | | | | | | | | | Page 13 of 40 | | | | | | | |
|  | | | | | | | | | | | | | | | | | |
|  | | | **Aggregate** |  | **Classification** |  | **Coverage** |  | **Number Of Coding References** | |  | **Reference Number** |  | **Coded By Initials** |  | **Modified On** |  |
|  | **Nodes\\Recommendations\Regulatory** | | | | | | | | | | | | | | | |  |
|  | | **Document** | | | | | | | | | | | | | | |  |
|  | | | **Files\\male\\IDI R05 male** | | | | | | | | | | | | | |  |
|  |  |  | Yes |  |  |  | 0.0341 |  | 1 | |  | | | | | |  |
|  | | |  |  |  |  |  |  |  | |  | | | | | | |
|  | | | | | | | | | | | | 1 |  |  |  |  |  |
|  | Respondent 1: So we have a limitation in that case and you people in the IRB can help us to be more stringent (Laughs) and you are like, no these things can be done here. You don’t have to do that.  Interviewer: So the IRB. So it is at several levels. You have the researcher level to build capacity then IRB.  Respondent 1: Ethical Committees.  Interviewer: Yes.  Respondent 2: Even at Government level.  Interviewer: Umm.  Respondent 2: Ultimately we have to develop capacity to do these things.  Interviewer: Umm.  Respondent 2: If there was the capacity to do these things within it would make the work of the IRB simpler.  Respondent 1: Simpler.  Interviewer: Then, tracking and monitoring usage. Because for most researchers will tell you once these samples leave Uganda, we lose control. You I mean you have a bio-repository. Have you put in place you know measures to monitor? You know samples. That are they use for the purposes for which they were collected. Do you have any mechanism? | | | | | | | | | | | | | | | |  |
|  |  |  |  |  |  |  |  |  |  |  |  |  |  |  |  |  |  |
|  |  |  |  |  |  |  |  |  |  |  |  |  |  |  |  |  |  |
|  |  | | | | | | | | | | | | | | | |  |
|  | | | **Files\\male\\IDI R09 Male** | | | | | | | | | | | | | |  |
|  |  |  | Yes |  |  |  | 0.0144 |  | 1 | |  | | | | | |  |
|  | | |  |  |  |  |  |  |  | |  | | | | | | |
|  | | | | | | | | | | | | 1 |  |  |  |  |  |
|  | R: I think we have covered quite a lot but I think what goes on is that this ethics. This is a new area where we need to build capacity for our selves both in ethics and it also goes in capacity building and the capability to handle these things locally and maybe redo regulations and keeping ahh keeping people. Ahh now the bio repository we are ahh still struggling keeping guidelines for the country. And then community engagement, the community needs to be engaged in whatever we do. So those are for me the take home things I can see. | | | | | | | | | | | | | | | |  |
| Reports\\Coding Summary By Code Report | | | | | | | | | | Page 14 of 40 | | | | | | | |
|  | | | | | | | | | | | | | | | | | |
|  | | | **Aggregate** |  | **Classification** |  | **Coverage** |  | **Number Of Coding References** | |  | **Reference Number** |  | **Coded By Initials** |  | **Modified On** |  |
|  | **Nodes\\Recommendations\Track samples** | | | | | | | | | | | | | | | |  |
|  | | **Document** | | | | | | | | | | | | | | |  |
|  | | | **Files\\male\\IDI R08 Male** | | | | | | | | | | | | | |  |
|  |  |  | Yes |  |  |  | 0.0546 |  | 1 | |  | | | | | |  |
|  | | |  |  |  |  |  |  |  | |  | | | | | | |
|  | | | | | | | | | | | | 1 |  |  |  |  |  |
|  | : So how ahh because like you have already alluded to it. You know like once samples leave here (R: you have no control) we don’t even know what happens to them. So is there, I mean do you have any suggestions of how you know... How can we try you know to track and monitor the usage of these samples? I mean in your own words how would you like it to be done? because we would like to look at the opinions of several ahh the lead researchers so that we can recommend to National council that maybe try this or that. Is there a way we can improve on the monitoring and tracking of this?  R: Laughs, there has to be a way of ensuring that people comply and one of the ways is to ask them to give reports on what they (I: hmm) do. But another definitive way is to see what they have done with it, although it is still possible for somebody to hide (laughs) what they have done with it. So maybe as part of the requirements in future they could require that when samples are shared in such a way resources are provided to enable the regulatory authorities to monitor what happens but working that out again is ahh it involves a lot of things. Or they may put in regulations which require institutions which they have on their side to monitor what the researchers do with those sample then they give reports (I: okay) to the regulatory authorities here (I: okay thanks very much) and the research collaboration. There needs to be some way of monitoring what they do with the samples either through the institutions there or involving the institutions here to monitor. | | | | | | | | | | | | | | | |  |
|  |  |  |  |  |  |  |  |  |  |  |  |  |  |  |  |  |  |
|  |  | | | | | | | | | | | | | | | |  |
|  | | | **Files\\male\\IDI R09 Male** | | | | | | | | | | | | | |  |
|  |  |  | Yes |  |  |  | 0.0340 |  | 2 | |  | | | | | |  |
|  | | |  |  |  |  |  |  |  | |  | | | | | | |
|  | | | | | | | | | | | | 1 |  |  |  |  |  |
|  | I: Of course, now another problem we as regulators of research are having. Once the samples leave this place, are there any mechanisms of tracking the samples?  R: surprisingly yes (I: okay) yes because you see, people can from where ever they come and they mess us up but once they are in their country, they fear ethics and legal issues. They fear to be caught on the wrong side of that ethics so of course if you don’t follow, they have a field day. But if you raised something, they will ask infact many times we have requested to return they will return. If you write and say return may things and account for whatever they will because they know they are ethically obligated. They don’t want to be caught ethically wanting because that is their career. They will be finished. | | | | | | | | | | | | | | | |  |
|  |  |  |  |  |  |  |  |  |  |  |  |  |  |  |  |  |  |
|  |  | | | | | | | | | | | | | | | |  |
|  | | | | | | | | | | | | 2 |  |  |  |  |  |
|  | : So that means our, in our situation it is laxity (R: laxity). Is it laxity or absence of laws and policy?  R: That I think the MTA gives you to do that. Already that is already given in the MTA but are you enforcing it? Do you really even think it is important? Now you realise that if the MTA is not well done you are losing capacity building. Your participants have consented in vain so you but you are there the guardian of this. You should put firm resources to this yeah. | | | | | | | | | | | | | | | |  |
| Reports\\Coding Summary By Code Report | | | | | | | | | | Page 15 of 40 | | | | | | | |
|  | | | | | | | | | | | | | | | | | |
|  | | | **Aggregate** |  | **Classification** |  | **Coverage** |  | **Number Of Coding References** | |  | **Reference Number** |  | **Coded By Initials** |  | **Modified On** |  |
|  | **Nodes\\Regulation of export of samples** | | | | | | | | | | | | | | | |  |
|  | | **Document** | | | | | | | | | | | | | | |  |
|  | | | **Files\\Female\\1DI R03 female** | | | | | | | | | | | | | |  |
|  |  |  | Yes |  | person |  | 0.0345 |  | 5 | |  | | | | | |  |
|  | | |  |  |  |  |  |  |  | |  | | | | | | |
|  | R: Ahh I don’t really, ahh I am not 100% ahh I have read through an MTA (I: yes) but I don’t deal with them on a regular (I: regular basis). Our regulatory affairs coordinator does that (I: yeah would have a better opinion) but I think these are things that should also revolve as we are revolving. Okay? So, if we had an MTA in the year 2000 (I: hmm) we cannot still have an MTA for the same things in 2019. We should be able as researchers, as collaborators, as IRBs to look at MTAs and say is this moving with the times? (I: okay) Is this absolutely necessary (I: hmm)? If it is not absolutely necessary, we can tell the funder that look we have labs, these are labs that are cap certified (I: yes) or these are bring them. | | | | | | | | | | | | | | | |  |
|  |  | | | | | | | | | | | | | | | |  |
|  | | | | | | | | | | | | 2 |  |  |  |  |  |
|  | R: MTAs? (I: ehh) through the IRBs and through the UNCST do that (I: okay) but we could even have ahh it become too cumbersome because some of those things, the IRBs are really busy (I: yes). We could have a small group of people yes to actually look at MTAs (I: MTAs). Yes, it will expedite things (I: hmm), you know you can look at an MTA and say why are you taking urine? (I: yes). | | | | | | | | | | | | | | | |  |
|  |  | | | | | | | | | | | | | | | |  |
|  | | | | | | | | | | | |  |  |  |  |  |  |
|  | R: MTAs? (I: ehh) through the IRBs and through the UNCST do that (I: okay) but we could even have ahh it become too cumbersome because some of those things, the IRBs are really busy (I: yes). We could have a small group of people yes to actually look at MTAs (I: MTAs). Yes, it will expedite things (I: hmm), you know you can look at an MTA and say why are you taking urine? (I: yes). | | | | | | | | | | | | | | | |  |
|  |  | | | | | | | | | | | | | | | |  |
|  | | | | | | | | | | | | 4 |  |  |  |  |  |
|  | R: Yeah but something that will not extra prolong (I: prolong) the review process of (I: of research) because when you took of NDA your like that’s a 4 months process but if something (I: yes) maybe it could be within an IRB (I: hmm) maybe they have 4 technical people or 3 because then MTAs need to be looked at because there some donors or funders who insist on taking specimens that shouldn’t be taken. If it gets out there that no our country is not going to let materials just go when we can develop capacity to do it here (I: hmm) then it will get to them soon (I: yeah). | | | | | | | | | | | | | | | |  |
|  |  | | | | | | | | | | | | | | | |  |
|  | | | | | | | | | | | | 5 |  |  |  |  |  |
|  | R: Yeah but something that will not extra prolong (I: prolong) the review process of (I: of research) because when you took of NDA your like that’s a 4 months process but if something (I: yes) maybe it could be within an IRB (I: hmm) maybe they have 4 technical people or 3 because then MTAs need to be looked at because there some donors or funders who insist on taking specimens that shouldn’t be taken. If it gets out there that no our country is not going to let materials just go when we can develop capacity to do it here (I: hmm) then it will get to them soon (I: yeah). | | | | | | | | | | | | | | | |  |
|  |  | | | | | | | | | | | | | | | |  |
|  | | | **Files\\Female\\IDI R14 female** | | | | | | | | | | | | | |  |
|  |  |  | Yes |  |  |  | 0.0558 |  | 2 | |  | | | | | |  |
|  | | |  |  |  |  |  |  |  | |  | | | | | | |
|  | | | | | | | | | | | | 1 |  |  |  |  |  |
|  | R: Ahh I think it is important because ahh one in terms of authorship (coughs, excuse me) ahh these samples have been collected here for example they are collected here In Uganda. So, if there is an MTA, there is that understanding that you know the international institute is not going to take ownership of the samples (I: hmm). Remember we have a home institute here so it’s very important and also protecting the samples so that they don’t take ownership of them while collected here. And whichever research we want to do, we want it to benefit the population from which it has been collected from (I: hmm). So, yes, it Is very good and important. | | | | | | | | | | | | | | | |  |
|  |  | | | | | | | | | | | | | | | |  |
|  | | | | | | | | | | | | | | | | | |
| Reports\\Coding Summary By Code Report | | | | | | | | | | Page 16 of 40 | | | | | | | |
|  | | | | | | | | | | | | | | | | | |
|  | | | **Aggregate** |  | **Classification** |  | **Coverage** |  | **Number Of Coding References** | |  | **Reference Number** |  | **Coded By Initials** |  | **Modified On** |  |
|  | | | | | | | | | | | | | | | | | |
|  | | | | | | | | | | | | 2 |  |  |  |  |  |
|  | R: How it can be regulated? (I: regulation of the export of samples where an investigator cannot do anything more if they develop another question) hmm I think that’s where the MTA comes in. yes that’s where the MTA comes in and it should detail what is going to be done with the samples and future possibilities (I:hmm). If the samples maybe go out to another institute, will they come back? Like who is in charge of those samples? I think it should all be detailed in the MTA (I:hmm). Yes, again if samples are shipped and the investigator has no control, then really how will the community benefit from where the samples were collected? Then also the authorship, like getting the credit for having done the research here is also important to motivate the researcher. Yes, I think it should be regulated maybe through an MTA yes and maybe while we are seeking ethical approval such issues should be clearly laid out for the researcher (I: hmm) for the whole protection, for protection for them and the samples and the data collected. | | | | | | | | | | | | | | | |  |
|  |  |  |  |  |  |  |  |  |  |  |  |  |  |  |  |  |  |
|  |  | | | | | | | | | | | | | | | |  |
|  | | | **Files\\Female\\IDI R15 female** | | | | | | | | | | | | | |  |
|  |  |  | Yes |  |  |  | 0.0525 |  | 3 | |  | | | | | |  |
|  | | |  |  |  |  |  |  |  | |  | | | | | | |
|  | | | | | | | | | | | | 1 |  |  |  |  |  |
|  | R: It’s important because that’s where all these statements are, what you will do with the sample, how long you will keep them, who owns the samples, all that information is there. So, it’s very important (I: hmm), however you will never ensure that it is enforced when it crosses. They won’t do more than they are supposed to and then also they will just use the data and then publish with no knowledge of anyone here. Those are the risks but hopefully you deal with people with integrity (both laugh) and all that should be catered for in the MTA and we have the courts to take note if there’s any breach on them, the MTAs. | | | | | | | | | | | | | | | |  |
|  |  | | | | | | | | | | | | | | | |  |
|  | | | | | | | | | | | | 2 |  |  |  |  |  |
|  | R: I have seen studies that ship samples for liver function tests (I: hmm), that shouldn’t be acceptable. If you want to ship for liver function tests and you validate results, to make sure you are accurate, you have to probably show, if I was sitting in that sit, I will say that show that other labs that you can’t validate with other labs internally and if you really want to validate externally, send 10% of the samples (I: hmm). You know what I mean and then see but I will never ship for any thing that can be done locally and I will need okay giving evidence can’t be done locally, that’s just like writing a sentence that we can’t do genotype. I would ship for things that really our local capacity can’t do and that’s what I will accept. And then putting in place MTA’s, there people who have been known not to ship samples without MTAs because the boss said and lab person doesn’t see the MTA. Because the MTA is seen by the people at the top and they know there is an MTA and just instruct the lab to prior go yeah. So, enforce the MTA’s, don’t ship un necessarily even when you ship there should be some conditions. You know when to destroy samples and evidence and instructions that the samples should be sent back, okay things like that. | | | | | | | | | | | | | | | |  |
|  |  |  |  |  |  |  |  |  |  |  |  |  |  |  |  |  |  |
|  |  | | | | | | | | | | | | | | | |  |
|  |  | | | | | | | | | | | | | | | |  |
|  | | | **Files\\male\\1DI R01 Male** | | | | | | | | | | | | | |  |
|  |  |  | Yes |  |  |  | 0.1092 |  | 5 | |  | | | | | |  |
|  | | |  |  |  |  |  |  |  | |  | | | | | | |
|  | | | | | | | | | | | | 1 |  |  |  |  |  |
|  | R: Mine has been, I think because we had a precedent with the team that I work with, the negotiations, we paid more attention to who or where the samples are going to be archived and used. For instance, there was a team we were working with in New York, we were not sure of their ability to handle our samples well yet they needed to do a certain procedure on them and then we thought well, because they are going to use the samples they should be the final storage point but we were not sure of their ability to store them well. We decided to actually to ship the sample to San Francisco because that’s where we have always shipped, stored and archived with confidence and been even able ship them back here. So the negotiations, I have not really had any unique challenges and also one of the things we (laughs) are running away from, the guys in New York are basic scientists so they tend to, they can easily put their hands in the cooking jar, oh yeah we have some samples over here, so we wanted to have some control over that. So, the team that I work with in San Francisco would say let us reduce their temptation, to do and yeah you need to know your ahh so part of the negotiations unfortunately you might need you to know your teams quite well, there certain tendencies certain groups will have, and you have to know them for the sake of the everyone’s peace and you know maintaining the rules of the game. | | | | | | | | | | | | | | | |  |
|  |  |  |  |  |  |  |  |  |  |  |  |  |  |  |  |  |  |
|  |  | | | | | | | | | | | | | | | |  |
| Reports\\Coding Summary By Code Report | | | | | | | | | | Page 17 of 40 | | | | | | | |
|  | | | | | | | | | | | | | | | | | |
|  | | | **Aggregate** |  | **Classification** |  | **Coverage** |  | **Number Of Coding References** | |  | **Reference Number** |  | **Coded By Initials** |  | **Modified On** |  |
|  | | | | | | | | | | | | | | | | | |
|  | | | | | | | | | | | | 2 |  |  |  |  |  |
|  | : It’s (I: it’s a hard one) it’s a hard one, you don’t want to over regulate because (I: what should we say, responsible regulation). I think it all starts with us knowing of course the purpose of this shipping, it has to be clear to everyone, why? But sometimes as I have talked to you, there reasons that are not so clear, so someone will have to prove beyond reasonable doubt that the shipment is necessary and there’s a truth that I have discovered also over time that this might come in the previous and may not come out directly and informs the decision. Some of these collaborators ship because a sample is an asset, it brings me PHD students, it brings me funding. I can use it to leverage many things, I can use it to leverage NIH funding, I can use it to get more PHD students in my lab to collaborate with a certain lab, I tell you, if you want this, I give you my samples, you give me some students that will work on this or a pharmaceutical company, it’s an asset. It’s a value, now I think we need to start seeing it that way. I don’t think most of our people see it that way, for us we just see it as blood going out. If I told you that when I was doing my checks in the US, they literally took off, I have never been bled that much but this was in a routine hospital check-up and because I was healthy, they could take as much blood as they thought. They took about 15 vacutainers a year and then you are signing papers, part of them were for research, for storage for what for the hospital. So, for our colleagues have realized that actually there’s gold in these samples (I: hmm). We should also realize that there’s gold in these sample that for me is precedence number one some people may just not realize it. So, it should be clearly told to investigators that if it’s going to be shipped out the purpose has to be clear that whatever reason it is a reason that we can’t at this point we are incapacitated to do.  Secondly, also we need to have local storage to destroy or control shipping, we need to have state of the art storage facilities with truck records that can be defensible and used as options. It will leave some money here some people might not like it because they lose the control, they lose the gold but if you can have an NIH ahh I think there some NIH funded biobanks and that stand to be reputable. They then become the night dust for us to say these guys can handle it and this is what you tell the study, it will be cheaper. For us it’s cheaper say every pop up I’m going to save 3,000$ every quarter to ship and I’m keeping samples here, the question is how much will these ones cost (I: hmm) ? Relatively but if we don’t have it one could argue and say that you don’t have constant power, you don’t have archival systems, you can’t retrieve, computerized storage is not there, if you don’t have these forget it, you will have to ship. | | | | | | | | | | | | | | | |  |
|  |  |  |  |  |  |  |  |  |  |  |  |  |  |  |  |  |  |
|  |  |  |  |  |  |  |  |  |  |  |  |  |  |  |  |  |  |
|  |  |  |  |  |  |  |  |  |  |  |  |  |  |  |  |  |  |
|  |  | | | | | | | | | | | | | | | |  |
|  | | | | | | | | | | | | 3 |  |  |  |  |  |
|  | R: Yes, I did, it somehow fits the mold I don’t have any truck records of it, how many studies it has stored and how many samples it has stored but I visited the infrastructure and it’s very convincing, and even the place it has to grow it’s very convincing, so. | | | | | | | | | | | | | | | |  |
|  |  | | | | | | | | | | | | | | | |  |
|  | | | | | | | | | | | | 4 |  |  |  |  |  |
|  | R: So, part of the regulation because you kind of make it hard without giving alternatives (I: hmm) so that flow of making people think twice or thrice (I: hmm) or four times, kind of has many arguments to it. It’s cheaper I have a better alternative, it’s easier to retrieve, if you need something, we can send it to you. In other words, start to make similar arguments (I: hmm) the third one it’s having very reputable labs if you have to catalogue these labs (I: hmm) and what do they do. One of the biggest challenges that I have always had is that we don’t have any like cache way of knowing what local labs can do (I: hmm) so that when you say that I’m going to ship, we will say but I can do that in my own lab but by the time I wrote this MTA to avoid all this trouble I didn’t know that you could and there’s no way I could find out. So may be as we put in our MTA’s and these reasons the National council has a list of updated research labs and what they can do, what their capacities are, what their accreditations are because accreditations also is a big deal because you can be able to do something but you have no accreditations to it and that can help make a case and it would be sewed, save money, save time and we kind of keep our gold. | | | | | | | | | | | | | | | |  |
|  |  |  |  |  |  |  |  |  |  |  |  |  |  |  |  |  |  |
|  |  | | | | | | | | | | | | | | | |  |
|  | | | | | | | | | | | | 5 |  |  |  |  |  |
|  | R: So, part of the regulation because you kind of make it hard without giving alternatives (I: hmm) so that flow of making people think twice or thrice (I: hmm) or four times, kind of has many arguments to it. It’s cheaper I have a better alternative, it’s easier to retrieve, if you need something, we can send it to you. In other words, start to make similar arguments (I: hmm) the third one it’s having very reputable labs if you have to catalogue these labs (I: hmm) and what do they do. One of the biggest challenges that I have always had is that we don’t have any like cache way of knowing what local labs can do (I: hmm) so that when you say that I’m going to ship, we will say but I can do that in my own lab but by the time I wrote this MTA to avoid all this trouble I didn’t know that you could and there’s no way I could find out. So may be as we put in our MTA’s and these reasons the National council has a list of updated research labs and what they can do, what their capacities are, what their accreditations are because accreditations also is a big deal because you can be able to do something but you have no accreditations to it and that can help make a case and it would be sewed, save money, save time and we kind of keep our gold. | | | | | | | | | | | | | | | |  |
|  |  |  |  |  |  |  |  |  |  |  |  |  |  |  |  |  |  |
|  |  | | | | | | | | | | | | | | | |  |
|  | | | **Files\\male\\1DI R04 Male** | | | | | | | | | | | | | |  |
|  |  |  | Yes |  |  |  | 0.0160 |  | 1 | |  | | | | | |  |
|  | | |  |  |  |  |  |  |  | |  | | | | | | |
|  | | | | | | | | | | | | 1 |  |  |  |  |  |
|  | R: Yes, they are, they are more or less the import and export for biological samples (laughs) going in and out to rules, this cross-border trade like but in biological samples (I: yes) and I think they kind of cover this. So, these are very important (I: hmm) because these are dangerous goods, sometimes you are shipping HIV blood, hepatitis blood, and all this. So, these cover these, they are important, very important otherwise we would be having free movement with infectious substances from one country to another, Ebola samples coming in and you don’t know they are Ebola samples and that is very dangerous (I: that’s dangerous). Yeah, so they are very good in controlling these dangerous goods. | | | | | | | | | | | | | | | |  |
|  |  | | | | | | | | | | | | | | | |  |
| Reports\\Coding Summary By Code Report | | | | | | | | | | Page 18 of 40 | | | | | | | |
|  | | | | | | | | | | | | | | | | | |
|  | | | **Aggregate** |  | **Classification** |  | **Coverage** |  | **Number Of Coding References** | |  | **Reference Number** |  | **Coded By Initials** |  | **Modified On** |  |
|  | | | **Files\\male\\1DI R12 Male** | | | | | | | | | | | | | |  |
|  |  |  | Yes |  | person |  | 0.0963 |  | 3 | |  | | | | | |  |
|  | | |  |  |  |  |  |  |  | |  | | | | | | |
|  | | | | | | | | | | | | 1 |  | DES |  |  |  |
|  | Resp: Material Transfer Agreements are very important and we commonly have these. You need to spell out, one; these Material Transfer Agreements are made between those providing the samples, those receiving them and those analysing them. There should be things spelt out to show that these samples will not be misused, they will be used ethically and after a particular purpose has been achieved then there should be a clear destruction process of those samples. Those Material Transfer Agreements have to be approved by the Uganda National Council of Science and Technology and it’s very important. | | | | | | | | | | | | | | | |  |
|  |  | | | | | | | | | | | | | | | |  |
|  | | | | | | | | | | | | 2 |  |  |  |  |  |
|  | Resp: We already have it in the country, once the Material Transfer Agreement is signed by all parties involved, those sending the samples, those receiving and those analysing the samples and those getting to destroy them after they have been used or returned. Sometimes the agreement is that you return them after using them. They are supposed to return whatever is left. They have ever returned a few but the problem was the cost. Who is going to pay for the cost of returning the samples?  Int: Who pays for that?  Resp: We had one where the people who analysed, returned the little that was left at their cost. That was the only one that has ever been returned but for all the others we have had them destroyed there. | | | | | | | | | | | | | | | |  |
|  |  |  |  |  |  |  |  |  |  |  |  |  |  |  |  |  |  |
|  |  | | | | | | | | | | | | | | | |  |
|  | | | | | | | | | | | | 3 |  |  |  |  |  |
|  | Int: How do you make sure they have destroyed them?  Resp: Now that’s the issue.  Int: Because you are not physically there.  Resp: But they are bound by the agreement you signed.  Int: To destroy!  Resp: Yes to destroy. | | | | | | | | | | | | | | | |  |
|  |  | | | | | | | | | | | | | | | |  |
|  | | | **Files\\male\\IDI R02 Male** | | | | | | | | | | | | | |  |
|  |  |  | Yes |  |  |  | 0.0334 |  | 3 | |  | | | | | |  |
|  | | |  |  |  |  |  |  |  | |  | | | | | | |
|  | | | | | | | | | | | | 1 |  | DES |  |  |  |
|  | Resp: They only protect you but really it’s just a piece of paper. Once the person gets that information and they take it to San Francisco then there is nothing you can do. | | | | | | | | | | | | | | | |  |
|  |  | | | | | | | | | | | | | | | |  |
|  | | | | | | | | | | | | 2 |  | DES |  |  |  |
|  | Resp: I mean practically how? We talked about the laws of the country, you will find that the laws that govern that sample the other side are totally different and they do not consider you. You find that the people that side consider this as a donation, so the laws that govern these countries dictate a lot. So you need to know these people before you send them samples. | | | | | | | | | | | | | | | |  |
|  |  | | | | | | | | | | | | | | | |  |
|  | | | | | | | | | | | | 3 |  | DES |  |  |  |
|  | Resp: I really don’t know because you see, when you have a Material Transfer Agreement, it just tells you that am going to transfer these materials to somebody to do this work with. But what if they decide not to do it the way you’ve written it, what will you do? | | | | | | | | | | | | | | | |  |
|  |  | | | | | | | | | | | | | | | |  |
|  | | | **Files\\male\\IDI R05 male** | | | | | | | | | | | | | |  |
|  |  |  | Yes |  |  |  | 0.0178 |  | 2 | |  | | | | | |  |
|  | | |  |  |  |  |  |  |  | |  | | | | | | |
|  | | | | | | | | | | | | 1 |  | DES |  |  |  |
|  | Respondent 1: We have MTAs I would say (Laughs). But like I said when it has gone it is very hard to follow it up. You know you’ve signed and the person has also accepted that this is what we are going to use the samples for but truthfully the fact that we are not going to be there, may be the only way we can trace it is if it is published. | | | | | | | | | | | | | | | |  |
|  |  | | | | | | | | | | | | | | | |  |
| Reports\\Coding Summary By Code Report | | | | | | | | | | Page 19 of 40 | | | | | | | |
|  | | | | | | | | | | | | | | | | | |
|  | | | **Aggregate** |  | **Classification** |  | **Coverage** |  | **Number Of Coding References** | |  | **Reference Number** |  | **Coded By Initials** |  | **Modified On** |  |
|  | | | | | | | | | | | | | | | | | |
|  | | | | | | | | | | | | 2 |  |  |  |  |  |
|  | Respondent 1: Then you are like but now we said it should be in this line. But still it will have been published (Laughs).  Respondent 2: East Africa has it right. There are MTAs | | | | | | | | | | | | | | | |  |
|  |  | | | | | | | | | | | | | | | |  |
|  | | | **Files\\male\\IDI R06 male** | | | | | | | | | | | | | |  |
|  |  |  | Yes |  |  |  | 0.1672 |  | 3 | |  | | | | | |  |
|  | | |  |  |  |  |  |  |  | |  | | | | | | |
|  | | | | | | | | | | | | 1 |  |  |  |  |  |
|  | R: Good question, (I: laughs) I think that’s where an informed consent form needs to be ahh very clear (I: hmm) that you are agreeing to so as a researcher, I am telling this individual this participant the potential participant that you are agreeing. By signing this consent form (I: hmm) you are agreeing to hand over your samples to me as a researcher because ahh even when you look at the material transfer agreements. Material transfer agreements are not between an IRB and an institution (I: hmm), they are between two investigators, collaborators or whatever it is with oversight of the National Council of Science and Technology in Uganda’s case and so you who designs the study and spoke to the patient and obtained the informed consent yeah you own the samples. If I have funded your research, I do not necessarily own the samples but the investigator who you have given responsibility and funding and the resources, they are the ones who own the samples. It needs to be very clear that even though the researcher says that by signing this form you are giving me ahh I would say temporary custody of your samples (I: hmm). It needs to be very clear that you can come to me any time and say now I don’t want the samples stored. During the course of the study, (I: hmm) it also needs to be very clear in the consent that we are going to store the samples for 10 ahh I agree that the UNCST. UNCST kind of has this broad ahh you can store samples for 10 years, 20 years ahh but yeah, those things are not really followed because we have had samples here for ahh (both laugh) but yeah that’s always the question if you give a time line. Are you tying yourself unnecessarily to saying ahh that of course the honours is on you to keep paying for whoever is storing those samples (I: hmm)? I think that becomes the only challenge with storing samples (I: hmm) but samples can potentially be stored you know for samples that were obtained from the Congo that were stored in Europe since the forties so ah yeah, I don’t think there should be a limitation (I: hmm) as such a statutory limitation for how long samples should be stored for that’s always a debate (I: hmm). Its fine to withdraw samples but the question is for how long? Who owns them? Is there a change in ownership if I am the investigator for study sponsored by IDI (I: hmm) if I move on to another place, does IDI now become the custodian of these samples (I: hmm)? I think ahh they are all these questions | | | | | | | | | | | | | | | |  |
|  |  |  |  |  |  |  |  |  |  |  |  |  |  |  |  |  |  |
|  |  |  |  |  |  |  |  |  |  |  |  |  |  |  |  |  |  |
|  |  | | | | | | | | | | | | | | | |  |
|  | | | | | | | | | | | | 2 |  |  |  |  |  |
|  | I: Okay and how important is the MTA? The material transfer agreement in cases where we have to transfer samples.  R: The MTA is important, MTAs are important because one it is also providing documentation that there is some collaborative agreement. I think you also don’t want research collaborations where people come here, they go to places withdraw samples. Fine consent people, let them with draw samples and the next thing they are on the plane and off. I think MTAs also give the context of this is operative ahh and that there is some context to exactly what will be shipped. And then the MTA gives an opportunity for collaborating institution to the one that is hosting the research (I: hmm) to be able to say look we also want to keep some of these samples. We have the capacity to do X, Y and Z. you don’t have to necessarily take all these samples (I: hmm) and then of course at the ahh what do you call it? At the airports as well (I: hmm) points of exit the MTA let me say ahh it gives context tow here you are carrying these samples. Why are you are shipping the samples? Ahh it has to be official (I: hmm) and so I know that a long time ago from the old HIV studies I think we used to come ahh of course there was no technology here. People would come and take samples and carry them in suitcases (I: hmm). So, there were no MTAs, there was nothing governing those relationships between investigators. So yeah, yeah I think MTAs are important. | | | | | | | | | | | | | | | |  |
|  |  |  |  |  |  |  |  |  |  |  |  |  |  |  |  |  |  |
| Reports\\Coding Summary By Code Report | | | | | | | | | | Page 20 of 40 | | | | | | | |
|  | | | | | | | | | | | | | | | | | |
|  | | | **Aggregate** |  | **Classification** |  | **Coverage** |  | **Number Of Coding References** | |  | **Reference Number** |  | **Coded By Initials** |  | **Modified On** |  |
|  | | | | | | | | | | | | | | | | | |
|  | | | | | | | | | | | | 3 |  | DES |  |  |  |
|  | I: Okay, so you will realise most or all samples are being transported out of ahh this country (R: yeah) ahh how do you think? Ahh what do you think should be done to regulate this?  R: I mean in the first place I think you ahh don’t want to use the word mitigate, you develop local capacity to be able to do a lot of the testing and potentially or the if the partners and collaborators say no shipping samples to do the testing. So yeah, I think building capacity locally is one important thing but two I agree that you can never have everything (I: hmm). So, it’s very rare to (laughs) have an MTA where samples are being shipped form the US to Uganda (I: laughs I know). I think it all dwells down to economics, if we were able to do all things here then I think there would be no ahh I mean you could have an MTA to take a few ahh quality control that is fine (I: hmm). That’s why you probably need MTAs (I: hmm) but yeah it just boils down to the economics. There just things we cannot do but at the same time if you I think if you look over the last ahh well one specific example (I: hmm) I think in 2007,8/9 we were shipping samples from IDI to the US to do ahh it’s what they call flusotomic (R: hmm) which is really looking at markers and cells things like that and there was this particular example where we lost like a thousand samples (I: hmm) just because of the relabelling and so on and so forth. They had to be taken out of the freezer, labelled because they had to put the ahh we had to label things and put them in the different lab management system (I: hmm). And so, when that happened obviously by the time the samples got to the US, they were all basically dead but in a few years later IDI got a flusotomy machine and so yeah now we don’t have to (I: ship samples) yeah. So, its ahh I guess the other example from IDI is measuring ahh the ability to measure drug levels. Again, that’s something that a few years ago IDI had to ship the samples to Europe. I mean you can now ahh the technology is here, people have been trained. So, it just goes back to my issue of we still need MTAs now (I: hmm) because of the differences in technology and what they are able to (I: do) yeah. But there ahh I would say that if everything else was available and it will obviously vary from one research study to another. If everything is available then the only reason you want to do that is to be able to do quality control. Otherwise you (I: don’t need it) yeah | | | | | | | | | | | | | | | |  |
|  |  |  |  |  |  |  |  |  |  |  |  |  |  |  |  |  |  |
|  |  |  |  |  |  |  |  |  |  |  |  |  |  |  |  |  |  |
|  |  | | | | | | | | | | | | | | | |  |
|  | | | **Files\\male\\IDI R07 male** | | | | | | | | | | | | | |  |
|  |  |  | Yes |  |  |  | 0.0685 |  | 2 | |  | | | | | |  |
|  | | |  |  |  |  |  |  |  | |  | | | | | | |
|  | | | | | | | | | | | | 1 |  | DES |  |  |  |
|  | I: Okay, now concerning this, you know like you’ve said that at times ahh the local researchers on the back foot. Now, one of the ways they can be protected is through negotiating material transfer agreements, okay. So, in your opinion, are these material transfer agreements any significant because in some cases, yes, they are negotiated but then the collaborators end up doing other things. How can this be enforced, how can they be enforced to ensure that you know, the MTAs?  R: In my experience, the MTAs have been working fairly well, we’ve also got materials from Europe and America but we are strictly confined to doing what we have agreed to do in that agreement and I would imagine the same applies to Ugandan materials. I would suggest If it doesn’t happen, then maybe the challenge is that there is no way we can monitor once we transfer materials outside the country. But for those countries they are a bit tough, even if you publish and they realize that what you have done is beyond the scope that was agreed on in the agreement, the article is retracted. So that is how serious they are, so even for us, the National council or institutions should find ways of follow up or investigation. | | | | | | | | | | | | | | | |  |
|  |  |  |  |  |  |  |  |  |  |  |  |  |  |  |  |  |  |
|  |  | | | | | | | | | | | | | | | |  |
|  | | | | | | | | | | | | 2 |  | DES |  |  |  |
|  | I: So how can this export of these samples be regulated? (R: export of samples) Because it looks like we have a problem, regulation is a problem.  R: Yes, to me I was thinking it is already regulated by National Council, they look at, they approve all the MTAs and I was thinking they are doing fairly well by agreeing transfer of materials where we have no technology and ensuring there is plan of that transfer of technology to local institutions. To me that would be sufficient at a National level but I know it can be abused. But National Council should step up its regulatory policies. | | | | | | | | | | | | | | | |  |
|  |  | | | | | | | | | | | | | | | |  |
|  | | | **Files\\male\\IDI R08 Male** | | | | | | | | | | | | | |  |
|  |  |  | Yes |  |  |  | 0.0905 |  | 3 | |  | | | | | |  |
|  | | |  |  |  |  |  |  |  | |  | | | | | | |
|  | | | | | | | | | | | | 1 |  |  |  |  |  |
|  | R: It’s the way of reinforcing to make sure it actually takes place because when the samples are taken and shown in the consent. First of all, in the consent that must be specified and somebody must agree to abide by it. And secondly before such samples are used, they need further clearance and then there must be a mechanism of trying to check to see what people have done with the samples. Did they follow all the conditions stipulated as a way of checking, follow up which seems not to be there because one these things go, they have gone. You don’t know what happens although they have written on paper. There should be away of follow up and checking to ensure compliance. (I: yeah that’s actually a very big problem) yeah. | | | | | | | | | | | | | | | |  |
|  |  | | | | | | | | | | | | | | | |  |
| Reports\\Coding Summary By Code Report | | | | | | | | | | Page 21 of 40 | | | | | | | |
|  | | | | | | | | | | | | | | | | | |
|  | | | **Aggregate** |  | **Classification** |  | **Coverage** |  | **Number Of Coding References** | |  | **Reference Number** |  | **Coded By Initials** |  | **Modified On** |  |
|  | | | | | | | | | | | | | | | | | |
|  | | | | | | | | | | | | 2 |  | DES |  | 2 |  |
|  | I: Thanks very much ahh now in this regard, in your opinion, what is the significance of ahh how important is an MTA? Because this one makes you or breaks you, whatever is in the MTA governs you know  R: An MTA is very important, it’s very important because it specifies the conditions, the terms and conditions for transfer of these samples. It is very important so | | | | | | | | | | | | | | | |  |
|  |  | | | | | | | | | | | | | | | |  |
|  | | | | | | | | | | | | 3 |  | DES |  |  |  |
|  | I: And how can this export of samples be regulated because a lot of samples have left this country. In your opinion how can we regulate? Of course, you have already said we can build our own capacity both infrastructural wise and you know maybe the human resource. Then anything how else can we you know regulate?  R: I don’t think we should move in quickly like try to do like some countries have done to bring in a regulation of no sample goes out of the country. I think that will be too a very extreme measure (I: hmm radical) it should be a gradual process, (I: hmm) a gradual process to deliberately remove this. I think the regulatory bodies should put in conditions to the system that if somebody wants to do research here (I: yeah) most of the work should be done within here depending on the capacities that they access and also try to make deliberate effort to build the capacity (M: hmm) because ahh people who come from out there might have some other hidden interests they might not want to build the capacity here yet they continue to get the samples. The institutions the government more so, should put in those efforts (I: hmm) to build these capabilities in here such that eventually we will reduce on what, transferring samples. I have not seen instances where we are getting samples from Europe and they are coming here in Africa (I: yeah everything is one way) everything is from here to there why? (I: hmm) So the government should put in efforts such that we reverse this. | | | | | | | | | | | | | | | |  |
|  |  |  |  |  |  |  |  |  |  |  |  |  |  |  |  |  |  |
|  |  | | | | | | | | | | | | | | | |  |
|  | | | **Files\\male\\IDI R09 Male** | | | | | | | | | | | | | |  |
|  |  |  | Yes |  |  |  | 0.0577 |  | 3 | |  | | | | | |  |
|  | | |  |  |  |  |  |  |  | |  | | | | | | |
|  | | | | | | | | | | | | 1 |  |  |  |  |  |
|  | : How can it be regulated of course we have, of course we have the role of the role of the material transfer agreement (R: hmm) how important do you think that document is and how can it be enforced?  R: Important, it is important and enforcement is another thing. On the other of course I am not going to say. Sometimes people sign it like they are signing any other document (laughs). They don’t know what they are signing and what they are giving away but I think a lot of effort should go on that material transfer agreement and that material transfer agreement should be enforced because that’s where the other things you are talking about commercialization, intellectual property is guided and of course it transfers the patient’s consent (I: hmm) because all of those must be taken care of because when transferring the material. The person where the material is going must ensure that consent is enforced and the use of the samples …. I think what we see, there is weakness there. In other wards the follow, the monitoring like the IRBs do and the RECs I don’t know. They try, they are supposed to try and monitor studies (I: hmm). They should put an effort in looking at what were the MTAs? What were they saying? Can they, can we see whether they were followed (I: hmm). Maybe they should randomly do some of them but what I see now that’s not happening. So, the institutions are left on their own even when the guidelines are saying these samples should not be taken because we have local capacity. They are just signing the MTAs; the MTA is killing local capacity. So that is a very important document for us which needs to be taken well, well care of. | | | | | | | | | | | | | | | |  |
|  |  |  |  |  |  |  |  |  |  |  |  |  |  |  |  |  |  |
|  |  | | | | | | | | | | | | | | | |  |
|  | | | | | | | | | | | | 2 |  |  |  |  |  |
|  | : How can it be regulated of course we have, of course we have the role of the role of the material transfer agreement (R: hmm) how important do you think that document is and how can it be enforced?  R: Important, it is important and enforcement is another thing. On the other of course I am not going to say. Sometimes people sign it like they are signing any other document (laughs). They don’t know what they are signing and what they are giving away but I think a lot of effort should go on that material transfer agreement and that material transfer agreement should be enforced because that’s where the other things you are talking about commercialization, intellectual property is guided and of course it transfers the patient’s consent (I: hmm) because all of those must be taken care of because when transferring the material. The person where the material is going must ensure that consent is enforced and the use of the samples …. I think what we see, there is weakness there. In other wards the follow, the monitoring like the IRBs do and the RECs I don’t know. They try, they are supposed to try and monitor studies (I: hmm). They should put an effort in looking at what were the MTAs? What were they saying? Can they, can we see whether they were followed (I: hmm). Maybe they should randomly do some of them but what I see now that’s not happening. So, the institutions are left on their own even when the guidelines are saying these samples should not be taken because we have local capacity. They are just signing the MTAs; the MTA is killing local capacity. So that is a very important document for us which needs to be taken well, well care of. | | | | | | | | | | | | | | | |  |
|  |  |  |  |  |  |  |  |  |  |  |  |  |  |  |  |  |  |
|  |  | | | | | | | | | | | | | | | |  |
|  | | | | | | | | | | | |  |  |  |  |  |  |
|  | : So that means our, in our situation it is laxity (R: laxity). Is it laxity or absence of laws and policy?  R: That I think the MTA gives you to do that. Already that is already given in the MTA but are you enforcing it? Do you really even think it is important? Now you realise that if the MTA is not well done you are losing capacity building. Your participants have consented in vain so you but you are there the guardian of this. You should put firm resources to this yeah. | | | | | | | | | | | | | | | |  |
|  |  | | | | | | | | | | | | | | | |  |
| Reports\\Coding Summary By Code Report | | | | | | | | | | Page 22 of 40 | | | | | | | |
|  | | | | | | | | | | | | | | | | | |
|  | | | **Aggregate** |  | **Classification** |  | **Coverage** |  | **Number Of Coding References** | |  | **Reference Number** |  | **Coded By Initials** |  | **Modified On** |  |
|  | | | **Files\\male\\IDI R10 male** | | | | | | | | | | | | | |  |
|  |  |  | Yes |  |  |  | 0.0749 |  | 3 | |  | | | | | |  |
|  | | |  |  |  |  |  |  |  | |  | | | | | | |
|  | | | | | | | | | | | |  |  |  |  |  |  |
|  | Respondent: On the conditions I think this is very important. The sharing of samples. Research being able to share isolates and other things across the world is very important. I mean I would give you an example uh of cholera which has different strains or flu for instance. Different strains. If flu broke out in Thailand and Thai people said we are not sharing the isolates then Sanofi Pasteur in whatever France will not be able to develop a vaccine because they do not have this particular variant or strain of uh the flu virus. So the sharing of samples is important depending on the circumstances. The conditions, policies you know the reigning environment underwhich everything is done I think is really what defines uh and these are things by the way you need to loo at in depth. So we’ve looked at a big picture of genomics research. But there are things that for instance the shipping of samples. Some one needs to develop clear samples like the Nagoya  Interviewer: The Nagoya Declaration. | | | | | | | | | | | | | | | |  |
|  |  |  |  |  |  |  |  |  |  |  |  |  |  |  |  |  |  |
|  |  | | | | | | | | | | | | | | | |  |
|  | | | | | | | | | | | |  |  |  |  |  |  |
|  | Interviewer: So describe how this export of samples how can be regulated because that is a very big problem.  Respondent: What I’m going to say is that uh I want to come from the developments of H3Africa Consortium because this is the first model of uh North to South Collaboration that we see where Africans have played a big role. Uh one of the things that was very key in establishing the H3Africa Consortium was establishing sub-committees that were running uh different activities and one of the most Important sub-committee was the one to do with uh the DBAC which was uh the Data and Biospecimen Access Committee. And this DBAC Committee defined policies under which circumstances, how you know mechanisms, conditions under which samples would be shared with western partners. For instance one of I think the requirements is that the sample cannot move by itself. It has move with a trainee you know and once the train returns with data yes but the data you know now when it comes to the open access model it ends up in public data bases and th DBAC goes ahead to define how long should it take for the data to be available in public databases in the sense that how long will the investigator take to exhaust what he wanted to do with this data before throwing it out there in the public database. I think that the H3Africa model presents s with a very good model in terms of international sharing of samples andi think that’s the point where we should look. It might have weaknesses but it is a place to startand it can be improved. | | | | | | | | | | | | | | | |  |
|  |  |  |  |  |  |  |  |  |  |  |  |  |  |  |  |  |  |
|  |  | | | | | | | | | | | | | | | |  |
|  | | | | | | | | | | | | 3 |  |  |  |  |  |
|  | Interviewer: How important is the MTA?  Respondent: Oh the Material Transfer Agreement is extremely important. I mean at the end of the day if you are shipping any biological to the U.S. without an MTA it will never pass the borders. The question is if you’re shipping to Uganda withot an MTA what will happen? And these issue even with an MTA, who is overseeing th implementation of the MTA? So I mean this is you know the MTA is an agreement between two parties uh to share materials but the whole issue is that uh I could just be a paper. It is the samething. Recently we were shipping materials to South Africa. TThere is no way you can cross the border without an MTA. These are important things so I think mechanisms for oversight are the most important things but it’s an important arrangement in he sense that it avoids future controversies. Uh it is a legal document that literally defines uh circumstances or conditions under which uh these two institutions of research are sharing samples or multiple institutions are sharing samples. So I think it’s uh very important but the key thing is still oversight. | | | | | | | | | | | | | | | |  |
|  |  |  |  |  |  |  |  |  |  |  |  |  |  |  |  |  |  |
|  |  | | | | | | | | | | | | | | | |  |
|  | | | **Files\\male\\IDI R11 male** | | | | | | | | | | | | | |  |
|  |  |  | Yes |  | person |  | 0.1379 |  | 3 | |  | | | | | |  |
|  | | |  |  |  |  |  |  |  | |  | | | | | | |
|  | | | | | | | | | | | | 1 |  |  |  |  |  |
|  | Interviewer: yeah, of course when you read the national guidelines, the participant is the owner, but the research institutions are the custodians and they are given the rights to use the samples as they so wish as long as the participant consented. ok, what do you think about the export and sharing of samples with international collaborators (of course, this is a question because there has been a lot of abuse of this ample sharing)  Respondent: that is a good one and at the same time a tricky one. but like I said, everything is grounded in certain contracts and agreements on how these samples should be handled  Interviewer: yeah, Material Transfer Agreements  Respondent: collaborations will always happen and all parties have interests, you may find yourself in a situation that if you fail to agree on issues like where the samples shall be analyzed from, you may not have the opportunity to participate in the research, or even an opportunity for capacity building so that may be in future you be able to analyses your own samples. so, in these collaborations, everyone comes with their interests and puts them on the table and I think it is all about getting affair resolution that will make all interested parties happy about the outcome. so sometimes it is necessary because we may not have capacity here | | | | | | | | | | | | | | | |  |
|  |  |  |  |  |  |  |  |  |  |  |  |  |  |  |  |  |  |
|  |  | | | | | | | | | | | | | | | |  |
| Reports\\Coding Summary By Code Report | | | | | | | | | | Page 23 of 40 | | | | | | | |
|  | | | | | | | | | | | | | | | | | |
|  | | | **Aggregate** |  | **Classification** |  | **Coverage** |  | **Number Of Coding References** | |  | **Reference Number** |  | **Coded By Initials** |  | **Modified On** |  |
|  | | | | | | | | | | | | | | | | | |
|  | | | | | | | | | | | | 2 |  |  |  |  |  |
|  | Interviewer: ok, describe how the export of samples can be regulated because it has been heavily abused and in some instances, you find that there are some people who move with samples in brief cases. how should this regulated because as a country and institutions, we need to come up with ways of trying to regulate this because it is too rampant. and of course what is more hurting, you export your samples, when you want to use them for future research, somebody tells you “we pay for these samples, you cannot use them. if you want just come this way and do whatever you want to do. and at times they give instructions to follow like you can do at your own cost.”  Respondent: umm  Interviewer: so in your opinion, how can we regulate the export of these samples?  Respondent: first and fore most like I said, if contracts still mean anything to people, i think that should be the best starting point | | | | | | | | | | | | | | | |  |
|  |  |  |  |  |  |  |  |  |  |  |  |  |  |  |  |  |  |
|  |  | | | | | | | | | | | | | | | |  |
|  | | | | | | | | | | | | 3 |  |  |  |  |  |
|  | Respondent: and the Material Transfer Agreement should stipulate/ able to provide for all the interests for both the provider and he recipient of the samples. and I believe by the time you sign these, both parties have reached an agreement on what to do with the samples, the rights, the restrictions how to access them. and in terms of bodies, of course you need the approval from UNCST regulate the transfer of materials between those two entities. but then, there should be legal frameworks to address any disagreements. like when there is bleach of the contract between the two parties there should be legal frameworks to address that and that is where we find a lot of issues resulting from; denying access of samples from one party and also using samples for un intended/ original research purposes. according to me, the tricky bit is, when samples go you are only relying on the good will of the other person. so does UNCST at regulatory level have to link up with another corresponding body to track or it is at institutional level?  Interviewer: may be let me ask something, whose responsibility is it to track the use of samples and ensuring that whatever is in the MTA is enforced? is it the researcher? is it the institution? is it the national regulatory body? you see there is a grey area there  Respondent: yes  Interviewer: and in some way or the other, bodies and the different stake holders keep counting on the other for certain responsibilities which are not being fulfilled due to lack of clarity on who should do what. in your opinion who should enforce?  Respondent: I think, the responsible should rely on both. there should be a level of inter- institutional follow up but also the regulatory body should play a role too. however, I do not know the intensity of this because there are so many researchers  Interviewer: yeah, they are so many  Respondent: and, on the side where the recipient is, there should an MTA with the regulatory body such that they are registered and can be monitored on how they are handling samples and whether the research being conducted on them is in line with the original objectives | | | | | | | | | | | | | | | |  |
|  |  |  |  |  |  |  |  |  |  |  |  |  |  |  |  |  |  |
|  |  |  |  |  |  |  |  |  |  |  |  |  |  |  |  |  |  |
|  |  | | | | | | | | | | | | | | | |  |
|  | | | **Files\\male\\IDI R13 male** | | | | | | | | | | | | | |  |
|  |  |  | Yes |  |  |  | 0.1070 |  | 3 | |  | | | | | |  |
|  | | |  |  |  |  |  |  |  | |  | | | | | | |
|  | Respondent: I think those are very cardinal and in this country, many people have been benefitting from this kind of thing and we need to take a closer look at it and make it something which is well protected and guarded. and for anything which goes out, a return request should be made once the process is done. and in fact in addition to that, I would propose that local researcher should visit where they are taking the samples and participate in the process of the analysis so that the moment the analysis is finished, he/ she can see how to have the samples back | | | | | | | | | | | | | | | |  |
|  |  | | | | | | | | | | | | | | | |  |
| Reports\\Coding Summary By Code Report | | | | | | | | | | Page 24 of 40 | | | | | | | |
| 2/18/2020 5:17 PM | | | | | | | | | | | | | | | | | |
|  | | | **Aggregate** |  | **Classification** |  | **Coverage** |  | **Number Of Coding References** | |  | **Reference Number** |  | **Coded By Initials** |  | **Modified On** |  |
|  | | | | | | | | | | | | | | | | | |
|  | | | | | | | | | | | | 3 |  |  |  |  |  |
|  | Interviewer: so, now basing on your opinion about this, how should this sample export be regulated? because you now we have a problem with rege just come in and take our samples. what they do with it there, we do not have control over it and even when they have done what they really obtained the consent for, they never return them. and in addition to that, even if they are returned, where are they stored? so for me I think it is something that we need to carefully look at, we need to strengthen it and we need to make it hard to take samples out of this country  ulation? how do you we can go about this because at the end of the day we shall have to make recommendations to UNCST coming from you as researchers  Respondent: I think there must be a rigorous process. first; any research which is done in Uganda, must have local researchers from Uganda who are not going to just be considered as research assistants but co- investigators. secondly, when that has been done, the time when there is need to go and do the analysis, if it possible, then it should be done here in Uganda and if seems impossible but the equipment can be brought in this country, it (equipment) should then be brought and analyzed in this country. if that is not possible, then in the process of the transfer of the materials, the co- investigator should escort the samples and go there, participate in the whole process of the analysis. once the analysis is completed, the person comes back with the samples that were taken so that these samples can be stored which is known. so, we now need to also have a place (kind of a national inventory/ repository for those kinds of samples) | | | | | | | | | | | | | | | |  |
|  |  |  |  |  |  |  |  |  |  |  |  |  |  |  |  |  |  |
|  |  | | | | | | | | | | | | | | | |  |
|  | **Nodes\\sample storage and ownership** | | | | | | | | | | | | | | | |  |
|  | | **Document** | | | | | | | | | | | | | | |  |
|  | | | **Files\\Female\\1DI R03 female** | | | | | | | | | | | | | |  |
|  |  |  | Yes |  | person |  | 0.0152 |  | 1 | |  | | | | | |  |
|  | | |  |  |  |  |  |  |  | |  | | | | | | |
|  | My opinion would have varied in 2004 and now in 2019.  I: Why?  R: 2004 we did not have the capacity to do half the tests that were necessary, (I: yes) we need to export the samples, 2019 we have built capacity to a level that we can do most of them (I: yes) we have literally hundreds of lab technicians or researchers who can actually do these tests. We may not have the machinery and whatever and I think it’s about time that we stop shipping the sample, let the researcher (I: come) actually come here, it’s more expensive to ship the sample than for one researcher to come with their question and tell us this is what I want to do. I want a group of ten lab technicians to help me do this, I think that is way cheaper for science and mankind. | | | | | | | | | | | | | | | |  |
|  |  |  |  |  |  |  |  |  |  |  |  |  |  |  |  |  |  |
|  |  | | | | | | | | | | | | | | | |  |
|  | | | **Files\\Female\\IDI R14 female** | | | | | | | | | | | | | |  |
|  |  |  | Yes |  |  |  | 0.0511 |  | 4 | |  | | | | | |  |
|  | | |  |  |  |  |  |  |  | |  | | | | | | |
|  | | | | | | | | | | | | 1 |  |  |  |  |  |
|  | R: I think it is very good (I: hmm) because one ahh it allows you in case there opportunities of further studying the subject area you are investigating you don’t have to go back to the participants (I: hmm) to consent and again, it’s better to already have a cohort of samples that are stored to study (I: hmm) maybe what you are interested in studying in the future. Ahh I think its cheaper than going back to enrol people afresh than collecting samples, consenting them and all. I think its cheaper if you have stored samples and you already obtained consent in the initial study if they already allowed the use of the samples for further studies. | | | | | | | | | | | | | | | |  |
|  |  | | | | | | | | | | | | | | | |  |
|  | | | | | | | | | | | | 2 |  |  |  |  |  |
|  | R: Ahh in a bio bank, yes in a bio bank under the right conditions they are supposed to be stored in. | | | | | | | | | | | | | | | |  |
|  |  | | | | | | | | | | | | | | | |  |
|  | | | | | | | | | | | | 3 |  |  |  |  |  |
|  | R: I think it’s the institution (I: why?) hmm one because the study is based at the institution (I:hmm) and ahh of course we collect the information from the participants (I:hmm) but it’s the institution that mothers that ahh how should I call it? It’s the institution that owns the study so its responsible for anything regarding the study (I: hmm). It’s not for the investigator because it’s not a personal thing for the investigator (I: hmm). So, I think it’s the institution that owns the study ahhh the information from the study. | | | | | | | | | | | | | | | |  |
|  |  | | | | | | | | | | | | | | | |  |
| Reports\\Coding Summary By Code Report | | | | | | | | | | Page 25 of 40 | | | | | | | |
| 2/18/2020 5:17 PM | | | | | | | | | | | | | | | | | |
|  | | | **Aggregate** |  | **Classification** |  | **Coverage** |  | **Number Of Coding References** | |  | **Reference Number** |  | **Coded By Initials** |  | **Modified On** |  |
|  | | | | | | | | | | | | | | | | | |
|  | | | | | | | | | | | | 4 |  |  |  |  |  |
|  | R: Yes, they may have a say on the information but they have given us consent to use their information unless if they withdraw. Yes, unless if they withdraw if they have given consent the institution kind of takes the ownership (I: alright thank you). | | | | | | | | | | | | | | | |  |
|  |  | | | | | | | | | | | | | | | |  |
|  | | | **Files\\Female\\IDI R15 female** | | | | | | | | | | | | | |  |
|  |  |  | Yes |  |  |  | 0.0872 |  | 5 | |  | | | | | |  |
|  | | |  |  |  |  |  |  |  | |  | | | | | | |
|  | | | | | | | | | | | | 1 |  |  |  |  |  |
|  | R: I do think we need it all the time so need to be positive, I, we need it, (laughs) it’s necessary. (I: it’s necessary) | | | | | | | | | | | | | | | |  |
|  |  | | | | | | | | | | | | | | | |  |
|  | | | | | | | | | | | | 2 |  |  |  |  |  |
|  | R: In a secure trust worthy lab, labs with good temperatures, good freezers you know those requirements to keep the samples viable (I: hmm). Ahh we store them in the institution we work in and that’s here (I: hmm). | | | | | | | | | | | | | | | |  |
|  |  | | | | | | | | | | | | | | | |  |
|  | | | | | | | | | | | | 3 |  |  |  |  |  |
|  | R: I think it’s the donor it’s the person who is ahh what do they call it in legal terms? who is responsible for the study. So, if it’s an IDI funded study, the samples will be owned by IDI. Unless you think it should be the patient, the patient can always say you know what? I want my samples. But for as long as the patient has consented for you to store the samples, I think they belong to that institute. I think if the investigator leaves the institute, if the investigator leaves the institute, still the institute, they still will remain with the institute (I: hmm can’t they leave with their samples?) I don’t think you can leave with samples; I don’t know (both laugh). Although I heard of research groups in the US who go with their data, they go with their everything. So, I guess there this might be a study for an individual, a PI led study where it’s my study, I’m the one leading it, I got the money you know but when it’s an institution, there it is different. Its hard to say samples belong to an individual (I: hmm). | | | | | | | | | | | | | | | |  |
|  |  |  |  |  |  |  |  |  |  |  |  |  |  |  |  |  |  |
|  |  | | | | | | | | | | | | | | | |  |
|  | | | | | | | | | | | | 4 |  |  |  |  |  |
|  | R: Coz many times the sponsor is the institution, the institution is the sponsor of the study. If it’s an institution like NIH, it’s an NIH funded study, but its given the money to an institution, so then I think they would remain at the institute and usually even when we are writing grants we always say that the samples belong to the institution but can be made available (I: hmm) yeah to interested parties (I: hmm okay, alright). | | | | | | | | | | | | | | | |  |
|  |  | | | | | | | | | | | | | | | |  |
|  | | | | | | | | | | | | 5 |  |  |  |  |  |
|  | R: I think it’s something that has its pros and cons, a lot of times, the samples go out there and they do more ahh you can’t govern what they do. They do more than what you agreed to do, but if they are responsible, institutions with integrity, it can be useful. We have to accept that our capacity is limited and even where we have capacity the costs can be really be high (I: hmm). So, it might be more cost effective to just be ship and analyse them and we can’t deny that many at times that this is collaborative effort so the roles can be dispersed but it’s up to the institute, the sponsor, the PI. They have to ensure that those samples are not misused, from my experience, we always send one sample leave one sample behind. You can’t say that I have no samples for this study, they all are in America, you always keep one behind and budget those costs. It’s a tricky issue because like you said knowledge is power and when they have your samples they will go and do the genotype. They will go and do everything they ever want to do. The day they decide to clone human beings they can clone all of Africa because they have the samples in a bio bank in the US (both laugh). So, it’s quite sensitive but I guess it depends on the integrity of the institute where you are (I: sending them) it’s tricky. | | | | | | | | | | | | | | | |  |
|  |  |  |  |  |  |  |  |  |  |  |  |  |  |  |  |  |  |
|  |  | | | | | | | | | | | | | | | |  |
|  | | | **Files\\male\\1DI R01 Male** | | | | | | | | | | | | | |  |
|  |  |  | Yes |  |  |  | 0.1214 |  | 6 | |  | | | | | |  |
|  | | |  |  |  |  |  |  |  | |  | | | | | | |
|  | | | | | | | | | | | | 1 |  |  |  |  |  |
|  | R: Me as a researcher, (I: hmm) I think it’s one of the efficient things that we can do to protect or to, if you get an opportunity and the patient accepts, for you to collect samples, store them, I think for me it’s an efficient way to build our research in this part of the world. Reason being is that for every sample that we collect, there is a unit dollar that spent and there’s unit time on the patient’s side spent and I would say that’s the most efficient thing that we could do and the only thing that happens after that is that for the samples that have been stored have to be used (I: hmm) in a very responsible manner. That’s for me I think my submission I think it’s one of the efficient things that we could do (I: so) if we can two things; catalogue the patients very well, the patients that gave us the samples, I can describe the age, whatever if it’s the type of sample tissue, sample, blood whatever we collected, I associate it with an individual whose clearly ahh whose characteristics I described. It’s a place for us to actually come back and do future research, I think it’s an efficient thing if we can do it well | | | | | | | | | | | | | | | |  |
|  |  |  |  |  |  |  |  |  |  |  |  |  |  |  |  |  |  |
|  |  | | | | | | | | | | | | | | | |  |
| Reports\\Coding Summary By Code Report | | | | | | | | | | Page 26 of 40 | | | | | | | |
|  | | | | | | | | | | | | | | | | | |
|  | | | **Aggregate** |  | **Classification** |  | **Coverage** |  | **Number Of Coding References** | |  | **Reference Number** |  | **Coded By Initials** |  | **Modified On** |  |
|  | | | | | | | | | | | | | | | | | |
|  | | | | | | | | | | | | 2 |  |  |  |  |  |
|  | R: laughs, well, I think in my opinion (I: I will tell you why I’m asking this question), what’s my thought about it. I think the ownership of these samples is a contract between the researcher and the patient. It’s a contract relationship, it’s not a single individual ownership, it’s a contractual ownership (I: hmm). I think at the point you are collecting the samples you sign a contract, the patient signed and whoever was on the researcher’s side also signed. It’s a contract ownership, dual ownership if I’m to answer that, without saying more words. | | | | | | | | | | | | | | | |  |
|  |  | | | | | | | | | | | | | | | |  |
|  | | | | | | | | | | | | 3 |  |  |  |  |  |
|  | R: I have not found in that quadrennium, actually the one that I have been found in is the one which sort of if I’m able to that three lettered word, EXC is when like you give some student or a colleague, when you work with a colleague on certain samples and goes ahead and does studies without informing me that one runs me mad because usually I know it happens and sometimes it happens and it’s like oh no we were just, it happens a lot with the lab people not with the clinician people, because I think the lab people get these questions in the head that they don’t even have the time to ask the clinician whether they can use the sample for that purpose. No, we had this hypothesis we were talking, we saw it in the meeting and I was like we have these samples we could test it and I used three of them and I said well even if it’s three, I need to know. | | | | | | | | | | | | | | | |  |
|  |  |  |  |  |  |  |  |  |  |  |  |  |  |  |  |  |  |
|  |  | | | | | | | | | | | | | | | |  |
|  | | | | | | | | | | | | 4 |  |  |  |  |  |
|  | R: Ehh I blasted some, I talked to the whites and that one I have no respect because I think it’s one of the risks. I think it’s one of the things, this one we don’t talk about very well, one of them is the risk of shipping, you want to have control over it but the question is that how much control? Because what we find ourselves doing sometimes is when we collect our samples in B and A component. I don’t know if you know this component ahh you know this archiving style, (I: hmm) so one of the things, the challenge has always been is that we have not had bio banks here and it’s been very hard to convince anybody that you can keep that and then one bio bank which I know which was here, it was just not well maintained and samples just got destroyed. In fact they literally poured freezers out of tissues, I have never seen, I actually looked at this guy telling me that their samples went bad and I was like I can’t believe you. And so that sort of sets us bad precedence but I think at some point we may have to just create a platform where you say well we give you “A” we stay with “B” use A. we need to inform the same individual, we split alcoates and stuff half way for our own scientific advancement. Of course, they will say that you don’t have the money. (I: That’s why) you don’t have the expertise to run somethings. | | | | | | | | | | | | | | | |  |
|  |  |  |  |  |  |  |  |  |  |  |  |  |  |  |  |  |  |
|  |  | | | | | | | | | | | | | | | |  |
|  | | | | | | | | | | | | 5 |  |  |  |  |  |
|  | R: Yeah, it has to be a very well governed situation, the challenge is, I mean this all starts off with our current collaboration style. For most part we call it collaboration, but there’s a power play here with the funding, it turns out that a lot of our funders, are at primes of these grants and control most of the money. So, until we are primes on grants and can control the money and control the funding, you know it’s going to be hard to run the collaborations but my view has always been that yes, if we say collaboration then it’s an equal war at this point. So, if ever anything is going to happen to any of these samples, we must know. It means that if I have a student here who needs those samples, you should be able to ship them back for me. It has happened actually we have done it before; they have shipped samples back and we have been able to run some assays on them that has happened. So, the precedent is there although it took some convincing that actually the person, we were going to work with was up to speed but I think it was. What we have observed sometimes is that and the truth is if you create local capacity, it will be cheaper to do complicated assays than it could have been in the USA or wherever. So, what is happening is that if we have good expertise, we have good labs we have people that can do some things that they could do else where I think that, it will be cheaper in the long run and everyone wants to do something that’s more affordable. The other bits yeah, I think the precedent has been set the other team that I work with we have some setbacks that have brought us where we are. | | | | | | | | | | | | | | | |  |
|  |  |  |  |  |  |  |  |  |  |  |  |  |  |  |  |  |  |
|  |  | | | | | | | | | | | | | | | |  |
|  | | | | | | | | | | | | 6 |  |  |  |  |  |
|  | Secondly, also we need to have local storage to destroy or control shipping, we need to have state of the art storage facilities with truck records that can be defensible and used as options. It will leave some money here some people might not like it because they lose the control, they lose the gold but if you can have an NIH ahh I think there some NIH funded biobanks and that stand to be reputable. They then become the night dust for us to say these guys can handle it and this is what you tell the study, it will be cheaper. For us it’s cheaper say every pop up I’m going to save 3,000$ every quarter to ship and I’m keeping samples here, the question is how much will these ones cost (I: hmm) ? Relatively but if we don’t have it one could argue and say that you don’t have constant power, you don’t have archival systems, you can’t retrieve, computerized storage is not there, if you don’t have these forget it, you will have to ship. | | | | | | | | | | | | | | | |  |
|  |  |  |  |  |  |  |  |  |  |  |  |  |  |  |  |  |  |
| Reports\\Coding Summary By Code Report | | | | | | | | | | Page 27 of 40 | | | | | | | |
|  | | | | | | | | | | | | | | | | | |
|  | | | **Aggregate** |  | **Classification** |  | **Coverage** |  | **Number Of Coding References** | |  | **Reference Number** |  | **Coded By Initials** |  | **Modified On** |  |
|  | | | **Files\\male\\1DI R04 Male** | | | | | | | | | | | | | |  |
|  |  |  | Yes |  |  |  | 0.0254 |  | 1 | |  | | | | | |  |
|  | | |  |  |  |  |  |  |  | |  | | | | | | |
|  | | | | | | | | | | | | 1 |  |  |  |  |  |
|  | R: So, this is my thinking, you know I’m the patient today (I: yes), I sign the informed consent assuming I have understood everything clearly explained and I make an informed decision, I’m giving away these samples, in writing I have accepted although I have the right to withdraw. As long as I have consented okay and I’m not expressing my right to withdraw the samples. These samples belong to the sponsor who has paid for the study okay (I: yes). They belong to the sponsor unless that condition of voluntariness comes in and I say I’m withdrawing (I: yes) so they belong to the sponsor (I: as long as you have accepted) as long as you have consented and everything is clear. It belongs to the sponsor because I’m willingly giving this. It’s like the blood that we donate to hospitals for treating patients who are lacking blood, you bet the point? You don’t own that blood any more, once you donate it, it’s for the government that has put in money to collect the blood (I: yes) and it knows what to do with the blood. (I: similar thing yes it seems like a public good now) yes, yeah, it’s a public good okay. | | | | | | | | | | | | | | | |  |
|  |  |  |  |  |  |  |  |  |  |  |  |  |  |  |  |  |  |
|  |  | | | | | | | | | | | | | | | |  |
|  | | | **Files\\male\\1DI R12 Male** | | | | | | | | | | | | | |  |
|  |  |  | Yes |  | person |  | 0.0646 |  | 3 | |  | | | | | |  |
|  | | |  |  |  |  |  |  |  | |  | | | | | | |
|  | | | | | | | | | | | | 1 |  |  |  |  |  |
|  | Resp: I have alluded to it, I believe in it a lot because it helps to research on rare diseases and those which occur in epidemics. It gives a greater advantage but it needs to be done ethically. | | | | | | | | | | | | | | | |  |
|  |  | | | | | | | | | | | | | | | |  |
|  | | | | | | | | | | | | 2 |  |  |  |  |  |
|  | Resp: To start with, the biological samples belong to the patients or the institution where the samples are obtained, for example if a research institution, like Makerere University College of Health Sciences or Department of Psychiatry or any department conducting the research and there is a principal investigator at that place collecting the data. But the samples are going to be shipped to a bio bank, let’s say like somewhere in the USA or a central bio bank even if it is here in Uganda and we don’t have many. So the initial owner is the patient and then for the collected samples it’s the primary institution but not the other one where samples are sent. There should be a material transfer agreement to agree on whether the samples are returned or get destroyed after they have been sent. But it’s the patient and the institution. | | | | | | | | | | | | | | | |  |
|  |  | | | | | | | | | | | | | | | |  |
|  | | | | | | | | | | | | 3 |  |  |  |  |  |
|  | Resp: To start with, the biological samples belong to the patients or the institution where the samples are obtained, for example if a research institution, like Makerere University College of Health Sciences or Department of Psychiatry or any department conducting the research and there is a principal investigator at that place collecting the data. But the samples are going to be shipped to a bio bank, let’s say like somewhere in the USA or a central bio bank even if it is here in Uganda and we don’t have many. So the initial owner is the patient and then for the collected samples it’s the primary institution but not the other one where samples are sent. There should be a material transfer agreement to agree on whether the samples are returned or get destroyed after they have been sent. But it’s the patient and the institution. | | | | | | | | | | | | | | | |  |
|  |  | | | | | | | | | | | | | | | |  |
|  | | | **Files\\male\\IDI R02 Male** | | | | | | | | | | | | | |  |
|  |  |  | Yes |  |  |  | 0.1587 |  | 10 | |  | | | | | |  |
|  | | |  |  |  |  |  |  |  | |  | | | | | | |
|  | | | | | | | | | | | | 1 |  |  |  |  |  |
|  | Resp: It’s a very dicey issue.Generally speaking it is good because these samples may be used to make discoveries that will help mankind in the bigger scheme of things. But then there is just too many uncertainties about it. Maybe the technology will evolve and we just realize that we just collected the wrong samples. I don’t know. Technology would evolve and then it reaches appoint, you realize, I used the wrong methods to collect this and we no longer need these kind of samples. You will have wasted the participants’ time. But I think storage of information is good because this information is extensively collected and people take time to collect, So you really don’t want to be collecting this kind of information all the time. | | | | | | | | | | | | | | | |  |
|  |  | | | | | | | | | | | | | | | |  |
|  | | | | | | | | | | | | 2 |  |  |  |  |  |
|  | Resp: Over and over again. You know you frustrate people and cause fatigue. | | | | | | | | | | | | | | | |  |
|  |  | | | | | | | | | | | | | | | |  |
|  | | | | | | | | | | | | 3 |  |  |  |  |  |
|  | Resp: Both the participant and the researcher. | | | | | | | | | | | | | | | |  |
|  |  | | | | | | | | | | | | | | | |  |
| Reports\\Coding Summary By Code Report | | | | | | | | | | Page 28 of 40 | | | | | | | |
|  | | | | | | | | | | | | | | | | | |
|  | | | **Aggregate** |  | **Classification** |  | **Coverage** |  | **Number Of Coding References** | |  | **Reference Number** |  | **Coded By Initials** |  | **Modified On** |  |
|  | | | | | | | | | | | | | | | | | |
|  | | | | | | | | | | | | 4 |  | DES |  | 1/24/2020 5:19 PM |  |
|  | Resp: Both the participant and the researcher. | | | | | | | | | | | | | | | |  |
|  |  | | | | | | | | | | | | | | | |  |
|  | | | | | | | | | | | | 5 |  | DES |  | 1/24/2020 5:19 PM |  |
|  | Resp: The participant is the one who gives you the physical sample. Right! | | | | | | | | | | | | | | | |  |
|  |  | | | | | | | | | | | | | | | |  |
|  | | | | | | | | | | | | 6 |  | DES |  | 1/24/2020 5:19 PM |  |
|  | Resp: Yes they have given it to you, but the question is have they donated it or they have given it? Because those are two distinct concepts. | | | | | | | | | | | | | | | |  |
|  |  | | | | | | | | | | | | | | | |  |
|  | | | | | | | | | | | | 7 |  | DES |  | 1/24/2020 5:19 PM |  |
|  | Resp: If they have donated it, then the researcher owns the rights bacause they have given up everything on it. But is that what we capture in our consents? I don’t know. If they have given it to you, then they remain with some partial ownership. It may not be explicit but it might just be implied. So are these people donating or giving samples? | | | | | | | | | | | | | | | |  |
|  |  | | | | | | | | | | | | | | | |  |
|  | | | | | | | | | | | | 8 |  | DES |  | 1/24/2020 5:19 PM |  |
|  | Resp: If they have donated it, then the researcher owns the rights bacause they have given up everything on it. But is that what we capture in our consents? I don’t know. If they have given it to you, then they remain with some partial ownership. It may not be explicit but it might just be implied. So are these people donating or giving samples? | | | | | | | | | | | | | | | |  |
|  |  | | | | | | | | | | | | | | | |  |
|  | | | | | | | | | | | | 9 |  | DES |  | 1/24/2020 5:20 PM |  |
|  | Resp: We need to be clear, that you have donated your sample and you will not see it again, like the way donors donate funds and that’s it. Once it’s gone its gone. If the other guys eat it the only thing you can do is to stop giving them more money but you cannot take them to prison. | | | | | | | | | | | | | | | |  |
|  |  | | | | | | | | | | | | | | | |  |
|  | | | | | | | | | | | | 10 |  | DES |  | 1/24/2020 5:23 PM |  |
|  | Int: Can you follow up?  Resp: How and using what methods?  Int: There nothing in place?  Resp: There is nothing. Can you sue them?  Int: Where can you sue them?  Resp: To whom? Do you have the money to sue a scientist in Tokyo?  Int: At Harvard.  Resp: You don’t have the money to sue them.  Int: You don’t have the resources.  Resp: You don’t even know them. You might be dealing with the PI but the PI may have three layers of bosses above them. So do you sue the university, do you sue the person or do you sue the project?  Int: Do you have the means, like for example to sue Harvard you as a scientist in Uganda?  Resp: No you don’t, you can just blackmail them but you cannot do much. You can only make enough noise in the media to show that these people came and stole samples from here but that’s where it ends. People will move on with their lives.  Int: You won’t get any more funding from them.  Resp: They will blacklist you. You blacklist them, they black list you but that’s where it ends. You may never visit their country. That’s where it ends. There is nothing you can do. If you don’t know them, that’s it. You are doomed.  Int: Hmm, ok.  Resp: I mean that’s like the practical thing. It’s just like how the donors send their funds here and we misappropriate it. What can they do? Nothing.  Int: They don’t give us more funding.  Resp: Yeah that’s where it ends.  Int: But we have taken.  Resp: They can force you because they are more powerful than you, to prosecute the people who have eaten the money.  Int: Seemingly prosecute.  Resp: Yeah but we see that it does not usually yield good results. But that’s ideally what we would. For example we would force the United States government to sue a scientist somewhere at Stanford University but are they going to do it?  Int: You don’t have the powers.  Resp: You don’t have the powers but you also don’t have the system. You may have the powers but you don’t have the system. You may not have good lawyers. Some of these things require state backing. The state might be the one to initiative that process. It cannot even if the community went up in arms. It ends there. | | | | | | | | | | | | | | | |  |
|  |  |  |  |  |  |  |  |  |  |  |  |  |  |  |  |  |  |
|  |  |  |  |  |  |  |  |  |  |  |  |  |  |  |  |  |  |
|  |  |  |  |  |  |  |  |  |  |  |  |  |  |  |  |  |  |
|  |  |  |  |  |  |  |  |  |  |  |  |  |  |  |  |  |  |
|  |  | | | | | | | | | | | | | | | |  |
| Reports\\Coding Summary By Code Report | | | | | | | | | | Page 29 of 40 | | | | | | | |
| 2/18/2020 5:17 PM | | | | | | | | | | | | | | | | | |
|  | | | **Aggregate** |  | **Classification** |  | **Coverage** |  | **Number Of Coding References** | |  | **Reference Number** |  | **Coded By Initials** |  | **Modified On** |  |
|  | | | **Files\\male\\IDI R05 male** | | | | | | | | | | | | | |  |
|  |  |  | Yes |  |  |  | 0.0685 |  | 8 | |  | | | | | |  |
|  | | |  |  |  |  |  |  |  | |  | | | | | | |
|  | | | | | | | | | | | | 1 |  | DES |  | 1/24/2020 5:28 PM |  |
|  | Respondent 1: (Laughs). It’s the best. | | | | | | | | | | | | | | | |  |
|  |  | | | | | | | | | | | | | | | |  |
|  | | | | | | | | | | | | 2 |  | DES |  | 1/24/2020 5:28 PM |  |
|  | Respondent 1: It is a very good idea uh to store sample for future research. Otherwise if we do not have them stored then we shall not have the future informed. | | | | | | | | | | | | | | | |  |
|  |  | | | | | | | | | | | | | | | |  |
|  | | | | | | | | | | | | 3 |  | DES |  | 1/24/2020 5:28 PM |  |
|  | Respondent 1: Because we can always go back to history and inform the current situation. A case in point is the drug resistance cases that we have. If really there is a reference from the back, for example mutations, uh previous mutations and the current mutations. | | | | | | | | | | | | | | | |  |
|  |  | | | | | | | | | | | | | | | |  |
|  | | | | | | | | | | | | 4 |  | DES |  | 1/24/2020 5:29 PM |  |
|  | Respondent 1: It is something that can be informative. So I think it is a very good thing to have samples stored.  Respondent 2: Yeah to add on, it is something that can tremendously bring down the cost of doing research. Uh you have these samples. You have accompanying phenotypic data. So a master’s student who does not have enough money can access the data, can access the sample and answer questions of interest. | | | | | | | | | | | | | | | |  |
|  |  | | | | | | | | | | | | | | | |  |
|  | | | | | | | | | | | | 5 |  | DES |  | 1/24/2020 5:31 PM |  |
|  | Respondent 2: Instead of going back to enroll, recruit, all sorts of things. | | | | | | | | | | | | | | | |  |
|  |  | | | | | | | | | | | | | | | |  |
|  | | | | | | | | | | | | 6 |  | DES |  | 1/24/2020 5:34 PM |  |
|  | Respondent 1: But when we discuss the costs you find that someone probably who has shipped is investing less than someone who has done it here.  Interviewer: Umm.  Respondent 1: Uh the expensive venture is that I think we need to be deliberate on this. We who are already in the field.  Interviewer: Umm.  Respondent 1: That we try our level best to bring things home so that in that way whoever wants to export, you tell them, but we can do that here. | | | | | | | | | | | | | | | |  |
|  |  |  |  |  |  |  |  |  |  |  |  |  |  |  |  |  |  |
|  |  | | | | | | | | | | | | | | | |  |
|  | | | | | | | | | | | | 7 |  | DES |  | 1/24/2020 5:38 PM |  |
|  | Respondent 1: We have MTAs I would say (Laughs). But like I said when it has gone it is very hard to follow it up. You know you’ve signed and the person has also accepted that this is what we are going to use the samples for but truthfully the fact that we are not going to be there, may be the only way we can trace it is if it is published. | | | | | | | | | | | | | | | |  |
|  |  | | | | | | | | | | | | | | | |  |
|  | | | | | | | | | | | | 8 |  | DES |  | 1/24/2020 5:39 PM |  |
|  | Respondent 2: They say when you publish you acknowledge them. They say if you use for the other, they can even go ahead and cause the withdrawal of your paper and embarrass you at that level as a way of trying to regulate usage of these things. | | | | | | | | | | | | | | | |  |
| Reports\\Coding Summary By Code Report | | | | | | | | | | Page 30 of 40 | | | | | | | |
| 2/18/2020 5:17 PM | | | | | | | | | | | | | | | | | |
|  | | | **Aggregate** |  | **Classification** |  | **Coverage** |  | **Number Of Coding References** | |  | **Reference Number** |  | **Coded By Initials** |  | **Modified On** |  |
|  | | | **Files\\male\\IDI R06 male** | | | | | | | | | | | | | |  |
|  |  |  | Yes |  |  |  | 0.1325 |  | 5 | |  | | | | | |  |
|  | | |  |  |  |  |  |  |  | |  | | | | | | |
|  | | | | | | | | | | | | 1 |  | DES |  | 2/13/2020 1:51 PM |  |
|  | R: I mean a common place would be nice. So ahh a good example I mean I could give you is CPHL (I: hmm). I mean I know that there is a bio repository there, is in the college yeah (I: hmm) but in the event that you have a place like CPHL where viral loads are peered in the night with hundreds of thousands of viral loads per year. I think broadly speaking that would be a good place to use as a repository. The questions of course ahh did people give consent? I think what becomes important is what did people give consent for (I: hmm) in terms of what to do with their samples if you store them (I: hmm). But yeah banking samples I think is important because one we know that there certain assays you cannot get a sample today, you do the tests, get another sample tomorrow you do another test. I mean it doesn’t make sense then you have to batch samples together. I mean that’s one huge advantage of storing samples especially things like immunological studies (I: hmm) or even genetic testing. It’s obviously more cost effective to batch samples together (I: hmm). Ahh there are the usual tests that you don’t need to do storage for but at the same time it’s always ahh questions always come up tomorrow, future (I: hmm) about patient populations that you had used before but unfortunately if you hadn’t collected the samples then you know you not going to go back to the same (I: patients) again (I: laughs). Yeah so, I think it is a good practice to think through as a researcher as you design your studies not just thinking about your own particular question this time (I: hmm) but thinking about the future as well. What might you yourself want to look at in future or what might other researchers want to look at in the future? So yeah 100% (I: important) yes, we should store samples | | | | | | | | | | | | | | | |  |
|  |  |  |  |  |  |  |  |  |  |  |  |  |  |  |  |  |  |
|  |  | | | | | | | | | | | | | | | |  |
|  | | | | | | | | | | | | 2 |  | DES |  | 2/13/2020 1:51 PM |  |
|  | R: I mean a common place would be nice. So ahh a good example I mean I could give you is CPHL (I: hmm). I mean I know that there is a bio repository there, is in the college yeah (I: hmm) but in the event that you have a place like CPHL where viral loads are peered in the night with hundreds of thousands of viral loads per year. I think broadly speaking that would be a good place to use as a repository. The questions of course ahh did people give consent? I think what becomes important is what did people give consent for (I: hmm) in terms of what to do with their samples if you store them (I: hmm). But yeah banking samples I think is important because one we know that there certain assays you cannot get a sample today, you do the tests, get another sample tomorrow you do another test. I mean it doesn’t make sense then you have to batch samples together. I mean that’s one huge advantage of storing samples especially things like immunological studies (I: hmm) or even genetic testing. It’s obviously more cost effective to batch samples together (I: hmm). Ahh there are the usual tests that you don’t need to do storage for but at the same time it’s always ahh questions always come up tomorrow, future (I: hmm) about patient populations that you had used before but unfortunately if you hadn’t collected the samples then you know you not going to go back to the same (I: patients) again (I: laughs). Yeah so, I think it is a good practice to think through as a researcher as you design your studies not just thinking about your own particular question this time (I: hmm) but thinking about the future as well. What might you yourself want to look at in future or what might other researchers want to look at in the future? So yeah 100% (I: important) yes, we should store samples | | | | | | | | | | | | | | | |  |
|  |  |  |  |  |  |  |  |  |  |  |  |  |  |  |  |  |  |
|  |  | | | | | | | | | | | | | | | |  |
|  | | | | | | | | | | | | 3 |  |  |  |  |  |
|  | R: Good question, (I: laughs) I think that’s where an informed consent form needs to be ahh very clear (I: hmm) that you are agreeing to so as a researcher, I am telling this individual this participant the potential participant that you are agreeing. By signing this consent form (I: hmm) you are agreeing to hand over your samples to me as a researcher because ahh even when you look at the material transfer agreements. Material transfer agreements are not between an IRB and an institution (I: hmm), they are between two investigators, collaborators or whatever it is with oversight of the National Council of Science and Technology in Uganda’s case and so you who designs the study and spoke to the patient and obtained the informed consent yeah you own the samples. If I have funded your research, I do not necessarily own the samples but the investigator who you have given responsibility and funding and the resources, they are the ones who own the samples. It needs to be very clear that even though the researcher says that by signing this form you are giving me ahh I would say temporary custody of your samples (I: hmm). It needs to be very clear that you can come to me any time and say now I don’t want the samples stored. During the course of the study, (I: hmm) it also needs to be very clear in the consent that we are going to store the samples for 10 ahh I agree that the UNCST. UNCST kind of has this broad ahh you can store samples for 10 years, 20 years ahh but yeah, those things are not really followed because we have had samples here for ahh (both laugh) but yeah that’s always the question if you give a time line. Are you tying yourself unnecessarily to saying ahh that of course the honours is on you to keep paying for whoever is storing those samples (I: hmm)? I think that becomes the only challenge with storing samples (I: hmm) but samples can potentially be stored you know for samples that were obtained from the Congo that were stored in Europe since the forties so ah yeah, I don’t think there should be a limitation (I: hmm) as such a statutory limitation for how long samples should be stored for that’s always a debate (I: hmm). Its fine to withdraw samples but the question is for how long? Who owns them? Is there a change in ownership if I am the investigator for study sponsored by IDI (I: hmm) if I move on to another place, does IDI now become the custodian of these samples (I: hmm)? I think ahh they are all these questions | | | | | | | | | | | | | | | |  |
|  |  |  |  |  |  |  |  |  |  |  |  |  |  |  |  |  |  |
|  |  |  |  |  |  |  |  |  |  |  |  |  |  |  |  |  |  |
|  |  | | | | | | | | | | | | | | | |  |
|  | | | | | | | | | | | | 4 |  |  |  |  |  |
|  | I: So, what do you think about that? If you have to leave IDI and you’re the investigator in key?  R: Yeah, no I think then there should be an institutional custody of those samples because then you know if I am not under that institution where the research was conducted where they sponsored that research (I: hmm). If I go to some other place and decide ahh first of all I may decide I am going to move with my samples (I: hmm) with those patient samples and who is going to provide oversight to what I do with the samples? I think it’s all those things there has to be some institutional oversight and that’s why the for example the Executive Director of whichever institution will sign off the material transfer agreement. Even if it’s between two investigators still the institution provides oversight. | | | | | | | | | | | | | | | |  |
|  |  | | | | | | | | | | | | | | | |  |
| Reports\\Coding Summary By Code Report | | | | | | | | | | Page 31 of 40 | | | | | | | |
|  | | | | | | | | | | | | | | | | | |
|  | | | **Aggregate** |  | **Classification** |  | **Coverage** |  | **Number Of Coding References** | |  | **Reference Number** |  | **Coded By Initials** |  | **Modified On** |  |
|  | | | | | | | | | | | | | | | | | |
|  | | | | | | | | | | | | 5 |  |  |  |  |  |
|  | I: So, what do you think about that? If you have to leave IDI and you’re the investigator in key?  R: Yeah, no I think then there should be an institutional custody of those samples because then you know if I am not under that institution where the research was conducted where they sponsored that research (I: hmm). If I go to some other place and decide ahh first of all I may decide I am going to move with my samples (I: hmm) with those patient samples and who is going to provide oversight to what I do with the samples? I think it’s all those things there has to be some institutional oversight and that’s why the for example the Executive Director of whichever institution will sign off the material transfer agreement. Even if it’s between two investigators still the institution provides oversight. | | | | | | | | | | | | | | | |  |
|  |  | | | | | | | | | | | | | | | |  |
|  | | | **Files\\male\\IDI R07 male** | | | | | | | | | | | | | |  |
|  |  |  | Yes |  |  |  | 0.0347 |  | 4 | |  | | | | | |  |
|  | | |  |  |  |  |  |  |  | |  | | | | | | |
|  | | | | | | | | | | | | 1 |  |  |  |  |  |
|  | R: To me it is amazing, we should encourage it because it is a new science in the area of bio depository and science in the issues of funding it is expensive. If you have to do a clinical trial or enroll participants in every study, it is very expensive but if we have well stored samples, we may realize that the costs are reduced by 50-60%. | | | | | | | | | | | | | | | |  |
|  |  | | | | | | | | | | | | | | | |  |
|  | | | | | | | | | | | | 2 |  |  |  |  |  |
|  | I: But isn’t storage of these samples in well-established depository storages expensive?  R: It is expensive; however, it is becoming a national issue because NIH has convinced the Ugandan government to take it on and it seems to be responsive. So, it will eventually be so cheap for researchers to store samples in such facilities. (I: Okay than storing them in their research labs) | | | | | | | | | | | | | | | |  |
|  |  | | | | | | | | | | | | | | | |  |
|  | | | | | | | | | | | | 3 |  |  |  |  |  |
|  | R: The sample is owned by the participant, the data is owned by the institutions and the researcher jointly because if there is any intellectual gap, we are bound by those policies. | | | | | | | | | | | | | | | |  |
|  |  | | | | | | | | | | | | | | | |  |
|  | | | | | | | | | | | | 4 |  |  |  |  |  |
|  | R: The sample is owned by the participant, the data is owned by the institutions and the researcher jointly because if there is any intellectual gap, we are bound by those policies. | | | | | | | | | | | | | | | |  |
|  |  | | | | | | | | | | | | | | | |  |
|  | | | **Files\\male\\IDI R08 Male** | | | | | | | | | | | | | |  |
|  |  |  | Yes |  |  |  | 0.1120 |  | 6 | |  | | | | | |  |
|  | | |  |  |  |  |  |  |  | |  | | | | | | |
|  | | | | | | | | | | | | 1 |  |  |  |  |  |
|  | : Where do you think this should be stored? Because right now we have like a repository here then different research units have their own repositories. Where do you think it should be? Should it be in a centralized ahh you know bio -bank, should every research study have their own? Because at the end of the day you want to look at the cost effectiveness because some of these samples have to be kept for very long (R: yes) and you need a full-time source of power, you need you know. (R: at the facility itself) In your opinion you know how would you like this to be done? An ideal situation | | | | | | | | | | | | | | | |  |
|  |  | | | | | | | | | | | | | | | |  |
|  | | | | | | | | | | | | 2 |  |  |  |  |  |
|  | : I don’t think it is feasible for every research to store these samples long term. Ahh maybe Nationally, at these facilities if there national labs that can store these samples for long term use and I think there hasn’t been a lot of effort to develop such particularly in developing countries. So, most samples end up going to developed countries where they are stored in the institution but I think it would be important to store these within but within the country it may not be feasible to have them stored in every research facility. If there is a national facility where such are stored, a national facility where such a stored maybe a facility affiliated to any academic institution like the universities. They keep that for future research, students come up and so on they need to do research in the future go back and revisit certain things. So that would be good instead of us taking all our samples out. So that even in the future when you want to look at something you have no samples (I: yeah). | | | | | | | | | | | | | | | |  |
|  |  |  |  |  |  |  |  |  |  |  |  |  |  |  |  |  |  |
|  |  | | | | | | | | | | | | | | | |  |
|  | | | | | | | | | | | | 3 |  |  |  |  |  |
|  | : I don’t think it is feasible for every research to store these samples long term. Ahh maybe Nationally, at these facilities if there national labs that can store these samples for long term use and I think there hasn’t been a lot of effort to develop such particularly in developing countries. So, most samples end up going to developed countries where they are stored in the institution but I think it would be important to store these within but within the country it may not be feasible to have them stored in every research facility. If there is a national facility where such are stored, a national facility where such a stored maybe a facility affiliated to any academic institution like the universities. They keep that for future research, students come up and so on they need to do research in the future go back and revisit certain things. So that would be good instead of us taking all our samples out. So that even in the future when you want to look at something you have no samples (I: yeah). | | | | | | | | | | | | | | | |  |
|  |  |  |  |  |  |  |  |  |  |  |  |  |  |  |  |  |  |
|  |  | | | | | | | | | | | | | | | |  |
| Reports\\Coding Summary By Code Report | | | | | | | | | | Page 32 of 40 | | | | | | | |
|  | | | | | | | | | | | | | | | | | |
|  | | | **Aggregate** |  | **Classification** |  | **Coverage** |  | **Number Of Coding References** | |  | **Reference Number** |  | **Coded By Initials** |  | **Modified On** |  |
|  | | | | | | | | | | | | | | | | | |
|  | | | | | | | | | | | | 4 |  |  |  |  |  |
|  | R: Laughs, ahh yeah, it’s a tricky one (I: is it the researcher, is it the participant, is it the collaborator, is it the institution?) Laughs it’s a tricky one. The samples come from the participant so it’s the participant who offers them to be used ahh so there different types of studies others are supported by sponsors who may be having different motives. Others are basically doing it purely for scientific research others are doing it to develop products for commercial purposes and they have different interests and of course all these researches are important (I: hmm). You need research done in the commercial entities like drug companies to develop new drugs for future use but for them they look at profit (I: yeah, they look at profit). Other researches are supported by other institutions that purely support scientific research to get new knowledge not with the interest of profit. So, it depends on which perspective you look at it (I: hmm). For somebody who wants to develop something with commercial interests they want to own it. | | | | | | | | | | | | | | | |  |
|  |  |  |  |  |  |  |  |  |  |  |  |  |  |  |  |  |  |
|  |  | | | | | | | | | | | | | | | |  |
|  | | | | | | | | | | | | 5 |  |  |  |  |  |
|  | I: Do you know what the Ugandan guidelines say?  R: Laughs, but if you to ask for my opinion, with the Ugandan guidelines specifically I am not sure, I am not sure. (I: they say that the donor is the owner but the institution like IDRC is a custodian okay). So yeah, I will think ultimately the country from which it comes should own, they should own those samples and have a say on what is done on the samples instead of surrendering them. So others when they work together with them, they should just agree and recognise who owns these things. | | | | | | | | | | | | | | | |  |
|  |  | | | | | | | | | | | | | | | |  |
|  | | | | | | | | | | | | 6 |  |  |  |  |  |
|  | I: Do you know what the Ugandan guidelines say?  R: Laughs, but if you to ask for my opinion, with the Ugandan guidelines specifically I am not sure, I am not sure. (I: they say that the donor is the owner but the institution like IDRC is a custodian okay). So yeah, I will think ultimately the country from which it comes should own, they should own those samples and have a say on what is done on the samples instead of surrendering them. So others when they work together with them, they should just agree and recognise who owns these things. | | | | | | | | | | | | | | | |  |
|  |  | | | | | | | | | | | | | | | |  |
|  | | | **Files\\male\\IDI R09 Male** | | | | | | | | | | | | | |  |
|  |  |  | Yes |  |  |  | 0.1623 |  | 7 | |  | | | | | |  |
|  | | |  |  |  |  |  |  |  | |  | | | | | | |
|  | | | | | | | | | | | | 1 |  |  |  |  |  |
|  | R: it’s a very important question hmm, storing samples for future research is very very important why? Because I have been discussing with very many people, it presents an opportunity for us to understand. Like if I, if you go with the samples for example here in pathology which were collected from 1956 up to now. It’s a very huge source, you can go and study how things have been changing (I: the trends over the years) the trends over the years. Secondly it gives us capacity for our very vibrant young people here who would like to do research but they have no resources to go and collect that information. This is available they can also be, participate in this situation and of course rather than throwing away samples ahh those samples can have very good use in future. So, storing samples is very good you may you might not be able rather than trade. Here the technology might not be available today to do this analysis but if the sample is there and the technology is available it is easier for you to get information. So there many benefits of sample storage | | | | | | | | | | | | | | | |  |
|  |  |  |  |  |  |  |  |  |  |  |  |  |  |  |  |  |  |
|  |  | | | | | | | | | | | | | | | |  |
|  | | | | | | | | | | | | 2 |  |  |  |  |  |
|  | I: Okay now you find that you have said, samples are scattered all over. You find like pathology have their own, maybe Case western have their own, repository, MUJHU. In your opinion how should these samples be stored? Where you know should institutions have like a centralized bio-bank? You know all samples from an institution are kept there. You know what would be your opinion on this?  R: Well first of all the institution should get interested (laughs) in what they are storing because that is another thing. They are not usually … Secondly for us in bio- banking there is what we call quality assured, well annotated samples. These samples should be of quality but well annotated. Annotated means well documented that you know a lot about the samples. Then if we go there, so if it is quality assurance it means they are collected well, transported well, stored well and know the conditions. So, it’s not a matter of having a bio a storage here which actually might give you false information when your analysing. So, if you are to achieve that, its as an institution, I think it might be easier to have one bank. Because that controlled environment to achieve it is not cheap (I: hmm). Everybody might say I am going to do it and its difficult but however if they can give it in various places at least they should be linked. Now technology is very easy there is what we call virtual bio constraint and link you here and link you here. That is what I want (I: hmm). That should be the less minimum because that resource, an institution like a training institution should be available to trainees so that they can easily access it and use it to advance. And of course there also issues of bio- terrorism, there issues of .. There issues I have also not told you about which are actually interesting. Sometimes people who talk about bio repository they don’t know what the bio repository does very well. Because for us we look at the bio repository call it a bio bank. Like its like a bank actually like when we deposit money in a bank, the bank doesn’t sit with that money (I: yeah it uses it). It uses it, it goes and gives you a loan and then in that case it multiplies your money but any time you want it, it gives you. Even us in bio repository we help to do that like if I get live cell, I can go and multiply them and make more aliquots (I: hmm). I can make your cells immortal that they live for ever so I can just give you but I keep propagating. All that brings ethical issues and the institutions should be aware of what is going on that’s why we keep a keen thing. How many immortal cells are? How many have you multiplied? How many aliquots of each do you have? That an institution should ahhh I know the National council of science has tried, attempted to do that but you can see there still very rudimentary. They don’t have very good tools and also probably where you are collecting the information. They are not well kept (laughs) (I: hmm) so they cannot even submit this information but we should keep this improved (I: okay). | | | | | | | | | | | | | | | |  |
|  |  |  |  |  |  |  |  |  |  |  |  |  |  |  |  |  |  |
|  |  |  |  |  |  |  |  |  |  |  |  |  |  |  |  |  |  |
|  |  |  |  |  |  |  |  |  |  |  |  |  |  |  |  |  |  |
|  |  | | | | | | | | | | | | | | | |  |
| Reports\\Coding Summary By Code Report | | | | | | | | | | Page 33 of 40 | | | | | | | |
|  | | | | | | | | | | | | | | | | | |
|  | | | **Aggregate** |  | **Classification** |  | **Coverage** |  | **Number Of Coding References** | |  | **Reference Number** |  | **Coded By Initials** |  | **Modified On** |  |
|  | | | | | | | | | | | | | | | | | |
|  | | | | | | | | | | | | 3 |  |  |  |  |  |
|  | I: Okay now you find that you have said, samples are scattered all over. You find like pathology have their own, maybe Case western have their own, repository, MUJHU. In your opinion how should these samples be stored? Where you know should institutions have like a centralized bio-bank? You know all samples from an institution are kept there. You know what would be your opinion on this?  R: Well first of all the institution should get interested (laughs) in what they are storing because that is another thing. They are not usually … Secondly for us in bio- banking there is what we call quality assured, well annotated samples. These samples should be of quality but well annotated. Annotated means well documented that you know a lot about the samples. Then if we go there, so if it is quality assurance it means they are collected well, transported well, stored well and know the conditions. So, it’s not a matter of having a bio a storage here which actually might give you false information when your analysing. So, if you are to achieve that, its as an institution, I think it might be easier to have one bank. Because that controlled environment to achieve it is not cheap (I: hmm). Everybody might say I am going to do it and its difficult but however if they can give it in various places at least they should be linked. Now technology is very easy there is what we call virtual bio constraint and link you here and link you here. That is what I want (I: hmm). That should be the less minimum because that resource, an institution like a training institution should be available to trainees so that they can easily access it and use it to advance. And of course there also issues of bio- terrorism, there issues of .. There issues I have also not told you about which are actually interesting. Sometimes people who talk about bio repository they don’t know what the bio repository does very well. Because for us we look at the bio repository call it a bio bank. Like its like a bank actually like when we deposit money in a bank, the bank doesn’t sit with that money (I: yeah it uses it). It uses it, it goes and gives you a loan and then in that case it multiplies your money but any time you want it, it gives you. Even us in bio repository we help to do that like if I get live cell, I can go and multiply them and make more aliquots (I: hmm). I can make your cells immortal that they live for ever so I can just give you but I keep propagating. All that brings ethical issues and the institutions should be aware of what is going on that’s why we keep a keen thing. How many immortal cells are? How many have you multiplied? How many aliquots of each do you have? That an institution should ahhh I know the National council of science has tried, attempted to do that but you can see there still very rudimentary. They don’t have very good tools and also probably where you are collecting the information. They are not well kept (laughs) (I: hmm) so they cannot even submit this information but we should keep this improved (I: okay). | | | | | | | | | | | | | | | |  |
|  |  |  |  |  |  |  |  |  |  |  |  |  |  |  |  |  |  |
|  |  |  |  |  |  |  |  |  |  |  |  |  |  |  |  |  |  |
|  |  |  |  |  |  |  |  |  |  |  |  |  |  |  |  |  |  |
|  |  | | | | | | | | | | | | | | | |  |
|  | | | | | | | | | | | | 4 |  |  |  |  |  |
|  | R: It depends, it depends of course the patient, the owner obviously would be the obvious que… answer but the practicability of that person owning that sample at a certain level becomes difficult because if I have sequenced the data the somehow that data at a certain point becomes retrievable. Like if its in the public domain, you have published there you don’t own ahh and I still have the sample but the data about that sample is gone. So, the patient has some ownership, the researcher has some ownership, the institution has some ownership as in the bio bank has some ownership (I: hmm) there. So, I think it is multi levelled (I: okay) yes. | | | | | | | | | | | | | | | |  |
|  |  | | | | | | | | | | | | | | | |  |
|  | | | | | | | | | | | | 5 |  |  |  |  |  |
|  | R: It depends, it depends of course the patient, the owner obviously would be the obvious que… answer but the practicability of that person owning that sample at a certain level becomes difficult because if I have sequenced the data the somehow that data at a certain point becomes retrievable. Like if its in the public domain, you have published there you don’t own ahh and I still have the sample but the data about that sample is gone. So, the patient has some ownership, the researcher has some ownership, the institution has some ownership as in the bio bank has some ownership (I: hmm) there. So, I think it is multi levelled (I: okay) yes. | | | | | | | | | | | | | | | |  |
|  |  | | | | | | | | | | | | | | | |  |
|  | | | | | | | | | | | | 6 |  |  |  |  |  |
|  | R: It depends, it depends of course the patient, the owner obviously would be the obvious que… answer but the practicability of that person owning that sample at a certain level becomes difficult because if I have sequenced the data the somehow that data at a certain point becomes retrievable. Like if its in the public domain, you have published there you don’t own ahh and I still have the sample but the data about that sample is gone. So, the patient has some ownership, the researcher has some ownership, the institution has some ownership as in the bio bank has some ownership (I: hmm) there. So, I think it is multi levelled (I: okay) yes. | | | | | | | | | | | | | | | |  |
|  |  | | | | | | | | | | | | | | | |  |
|  | | | | | | | | | | | | 7 |  |  |  |  |  |
|  | I: Because as per the Ugandan guidelines the donor is the owner (I: yes) but the institution keeping for example right now its Max Agency is the custodian because ahh the problem comes when you know a researcher owns samples and you want to leave like Makerere to go elsewhere they want to go with the samples (R: hmm). Actually, there was a court case in the US and of course this researcher actually took some samples and they sued him and he had to take them back. So that one has been some bit of a problem. Of course, you need robust guidelines you know and policy documents to guide yeah (R: to guide that) go give guidance on this.  R: But on that point, supposing I get a patent and I am paid that issue of ownership becomes another problem (I: yes) (laughs) who owns it? Because if I agree you are the donor, you are the owner of the sample but sometimes the patent comes from analysis putting together this and comparing with this. I think this one is a drug target or we can create a vaccine then I patent it. Now you see?  I: Yes of course there so many ethical issues of course commercialization, intellectual property rights. You know how are these benefits shared with the local researchers and the communities? So just two more questions (R: yes) | | | | | | | | | | | | | | | |  |
|  |  |  |  |  |  |  |  |  |  |  |  |  |  |  |  |  |  |
| Reports\\Coding Summary By Code Report | | | | | | | | | | Page 34 of 40 | | | | | | | |
|  | | | | | | | | | | | | | | | | | |
|  | | | **Aggregate** |  | **Classification** |  | **Coverage** |  | **Number Of Coding References** | |  | **Reference Number** |  | **Coded By Initials** |  | **Modified On** |  |
|  | | | **Files\\male\\IDI R10 male** | | | | | | | | | | | | | |  |
|  |  |  | Yes |  |  |  | 0.1688 |  | 21 | |  | | | | | |  |
|  | | |  |  |  |  |  |  |  | |  | | | | | | |
|  | | | | | | | | | | | | 1 |  | DES |  |  |  |
|  | Interviewer: What do you think of storing samples for future research?  Respondent: I think it is a good idea. Good extremely brilliant idea. So whoever came up with this kind of idea should have won a Nobel Prize  Interviewer: Why?  Respondent: I would give you my reasons. Number one uh collecting samples uh is resource intensive. Number one. But at the same time uh it has uh interventions that uh invasive to the patient and these interventions cause pain, they sometimes cause trauma uh and we waste quite a lot of resources in terms of collecting samples. Uh you waste the patient’s time but at the same time you are wasting the researcher’s time and money and other things and I think that uh given the chance, if there is a way any study can be done without having to go to the community and actively collect the sample. If there is a way. Because there are studies you can do that way. Clinical trials for instance you must interface with the patient. But if there is a way and the samples are available I think it’s a good idea.  Interviewer: So in your opinion it’s a very good idea.  Respondent: Yes. Brilliant idea.  Interviewer: Where should they be stored? Because now you find that everybody, every department you know they have their own fridges and it’s now difficult to take stock of you know what is where and if I wanted use instead of going to the field may be if there is somebody you know. In your opinion.  Respondent: So before we go far I think I need to qualify my answer.  Interviewer: Umm.  Respondent: That said, there are practices, dos and don’ts of bio-banking.  Interviewer: Umm.  Respondent: How samples should be handled and uh if samples have not been well handled then at the end of the day it doesn’t serve the purpose. So even when I’m saying this is a brilliant idea the answer needs to be qualified in the sense that it has to be done the right way. And this is why I was talking about the issue, that there is a lot of work for IRBs to do, in the sense for instance of following up the tailored consent. Which samples have expired? If the samples have expired and the IRB is responsible enough they should apply for a waiver from the IRB to extend maybe another five years that these samples can be accessed by researchers. And that should be done but you will find that that is not done and largely because of uh the lack of uh stringency following up the expiry of this consent. The timeline for consent and so that wouldn’t be right. So but when you ask me about where the samples should be stored, I think that we need to rethink how we approach this. The other day I was with h a colleague of mine and he was asking me: Misaki do you have a lab? I was like no you need to understand this is Africa. I do not need to have my own lab. We have established central labs. Do you know what I am saying? | | | | | | | | | | | | | | | |  |
|  |  |  |  |  |  |  |  |  |  |  |  |  |  |  |  |  |  |
|  |  |  |  |  |  |  |  |  |  |  |  |  |  |  |  |  |  |
|  |  |  |  |  |  |  |  |  |  |  |  |  |  |  |  |  |  |
|  |  | | | | | | | | | | | | | | | |  |
|  | | | | | | | | | | | | 2 |  |  |  |  |  |
|  | Respondent: And so economies of scale dictate that we have a few central places where we all operate. Yes I have an independent team that I worked with, we have our own research agenda but it is expensive to run labs. And so we need to think about centralized storage for samples and uh this has been a challenge because on the global level there has not yet been a clear accreditation mechanism for bio-banks. Even in the U.S. there is I think what we call ISO accreditation for bio-banks but you know the ISO is more of a commercial rather than a scientific and ethical you know. So all these things, I think these are things that need to be defined. We were lucky when we were awarded the integrated bio-repository. By the way the integrated bio-repository is not the first bio-repository within the Ugandan context. | | | | | | | | | | | | | | | |  |
|  |  | | | | | | | | | | | | | | | |  |
|  | | | | | | | | | | | | 3 |  |  |  |  |  |
|  | Respondent: And so economies of scale dictate that we have a few central places where we all operate. Yes I have an independent team that I worked with, we have our own research agenda but it is expensive to run labs. And so we need to think about centralized storage for samples and uh this has been a challenge because on the global level there has not yet been a clear accreditation mechanism for bio-banks. Even in the U.S. there is I think what we call ISO accreditation for bio-banks but you know the ISO is more of a commercial rather than a scientific and ethical you know. So all these things, I think these are things that need to be defined. We were lucky when we were awarded the integrated bio-repository. By the way the integrated bio-repository is not the first bio-repository within the Ugandan context. | | | | | | | | | | | | | | | |  |
|  |  | | | | | | | | | | | | | | | |  |
|  | | | | | | | | | | | | 4 |  |  |  |  |  |
|  | Respondent: People have been storing samples all over. I think people from uh the Ministry of Agriculture are doing quite a lot of storage of sperms and other things in Entebbe but this was the first time we were interfacing Council on issue to do with bio-banking because the funding mechanisms require that we get IRB approval, we get national clearance to run the bio-bank. | | | | | | | | | | | | | | | |  |
|  |  | | | | | | | | | | | | | | | |  |
|  | | | | | | | | | | | | 5 |  |  |  |  |  |
|  | Respondent: And it’s amazing that uh he protocols we developed ended up becoming the protocols adopted by Council for Science and Technology for bio-banking. And a question to ask: should it be central? Yes and No. Yes and no because it depends on how fast do you want to use the sample. If you want to use the sample fast you should have your own storage and that storage should be run in such a way that uh may be the bigger bio-bank that is accredited comes in and does some level of training of what should be done. And then you have your storage locally and once those samples are done you need to have plan on what you are going to do with the samples maybe to push them in the longer storage. But the reason I say yes and no because largely uh the bio-bank operates for long term storage. | | | | | | | | | | | | | | | |  |
|  |  | | | | | | | | | | | | | | | |  |
| Reports\\Coding Summary By Code Report | | | | | | | | | | Page 35 of 40 | | | | | | | |
|  | | | | | | | | | | | | | | | | | |
|  | | | **Aggregate** |  | **Classification** |  | **Coverage** |  | **Number Of Coding References** | |  | **Reference Number** |  | **Coded By Initials** |  | **Modified On** |  |
|  | | | | | | | | | | | | | | | | | |
|  | | | | | | | | | | | | 6 |  |  |  |  |  |
|  | Respondent: You know so the central bio-bank should really be focused on long term storage. | | | | | | | | | | | | | | | |  |
|  |  | | | | | | | | | | | | | | | |  |
|  | | | | | | | | | | | | 7 |  |  |  |  |  |
|  | Respondent: You know so the central bio-bank should really be focused on long term storage. | | | | | | | | | | | | | | | |  |
|  |  | | | | | | | | | | | | | | | |  |
|  | | | | | | | | | | | | 8 |  |  |  |  |  |
|  | Interviewer: Thanks very much. Who owns the samples in your opinion? Who owns the samples and their associated data?  Respondent: Let’s dissect this because when you say the sample and associated data  Interviewer: Sample ownership.  Respondent: sample ownership is the patient and IRB. | | | | | | | | | | | | | | | |  |
|  |  | | | | | | | | | | | | | | | |  |
|  | | | | | | | | | | | | 9 |  |  |  |  |  |
|  | Interviewer: Thanks very much. Who owns the samples in your opinion? Who owns the samples and their associated data?  Respondent: Let’s dissect this because when you say the sample and associated data  Interviewer: Sample ownership.  Respondent: sample ownership is the patient and IRB. | | | | | | | | | | | | | | | |  |
|  |  | | | | | | | | | | | | | | | |  |
|  | | | | | | | | | | | | 10 |  |  |  |  |  |
|  | Respondent: Always remains. That is why I am saying the IRB should follow up in terms of, you are doing this on behalf of the patient. This patient when he offers he is counting on you because you are the one that approved this study. He is not counting on the researcher. | | | | | | | | | | | | | | | |  |
|  |  | | | | | | | | | | | | | | | |  |
|  | | | | | | | | | | | | 11 |  |  |  |  |  |
|  | Respondent: When you expand and say ok the bio-bank is a custodian. Simply a custodian. They don’t own these samples but in terms of ownership of the sample the sample belongs to the patient. But the patient offers this sample to the researcher. You know what I’m saying. Over the period of time and depending on the type of consent the patient still has control over that sample. So even when you re looking at this sample. Yes it it has been given to the researcher, if the researcher misuses that sample the patient has a right. The IRBs have a right to still come in. | | | | | | | | | | | | | | | |  |
|  |  | | | | | | | | | | | | | | | |  |
|  | | | | | | | | | | | | 12 |  |  |  |  |  |
|  | Respondent: When you expand and say ok the bio-bank is a custodian. Simply a custodian. They don’t own these samples but in terms of ownership of the sample the sample belongs to the patient. But the patient offers this sample to the researcher. You know what I’m saying. Over the period of time and depending on the type of consent the patient still has control over that sample. So even when you re looking at this sample. Yes it it has been given to the researcher, if the researcher misuses that sample the patient has a right. The IRBs have a right to still come in. | | | | | | | | | | | | | | | |  |
|  |  | | | | | | | | | | | | | | | |  |
|  | | | | | | | | | | | | 13 |  |  |  |  |  |
|  | Respondent: When you expand and say ok the bio-bank is a custodian. Simply a custodian. They don’t own these samples but in terms of ownership of the sample the sample belongs to the patient. But the patient offers this sample to the researcher. You know what I’m saying. Over the period of time and depending on the type of consent the patient still has control over that sample. So even when you re looking at this sample. Yes it it has been given to the researcher, if the researcher misuses that sample the patient has a right. The IRBs have a right to still come in. | | | | | | | | | | | | | | | |  |
|  |  | | | | | | | | | | | | | | | |  |
|  | | | | | | | | | | | | 14 |  |  |  |  |  |
|  | Interviewer: So there is this situation a researcher talks of my samples my samples  Respondent: Yes.  Interviewer: A researcher today with Makerere and wants you know to go to another university and wants to,  Respondent: To take their samples with them.  Interviewer: With them you know. | | | | | | | | | | | | | | | |  |
|  |  | | | | | | | | | | | | | | | |  |
|  | | | | | | | | | | | | 15 |  |  |  |  |  |
|  | Respondent: Laughs. Now that’s an interesting one. You see in reality yes the patient has offered you know has offered consent, given a level of ownership to these sampes to the researcher and for the interest of I think biological research it is important we respect that ownership the investigator has because many times it is the investigator that appreciates the value of this sample. This sample is important to this investigator because uh they came out with a particular hypothesis. They wanted to answer a certain question. May be the question has not been answered and so it is important to respect that. The issue of moving with samples left to right it is another thing because this is where you come in as the IRB. | | | | | | | | | | | | | | | |  |
|  |  | | | | | | | | | | | | | | | |  |
| Reports\\Coding Summary By Code Report | | | | | | | | | | Page 36 of 40 | | | | | | | |
|  | | | | | | | | | | | | | | | | | |
|  | | | **Aggregate** |  | **Classification** |  | **Coverage** |  | **Number Of Coding References** | |  | **Reference Number** |  | **Coded By Initials** |  | **Modified On** |  |
|  | | | | | | | | | | | | | | | | | |
|  | | | | | | | | | | | | 16 |  | DES |  |  |  |
|  | Respondent: Laughs. Now that’s an interesting one. You see in reality yes the patient has offered you know has offered consent, given a level of ownership to these sampes to the researcher and for the interest of I think biological research it is important we respect that ownership the investigator has because many times it is the investigator that appreciates the value of this sample. This sample is important to this investigator because uh they came out with a particular hypothesis. They wanted to answer a certain question. May be the question has not been answered and so it is important to respect that. The issue of moving with samples left to right it is another thing because this is where you come in as the IRB. | | | | | | | | | | | | | | | |  |
|  |  | | | | | | | | | | | | | | | |  |
|  | | | | | | | | | | | | 17 |  |  |  |  |  |
|  | Respondent: Laughs. Now that’s an interesting one. You see in reality yes the patient has offered you know has offered consent, given a level of ownership to these sampes to the researcher and for the interest of I think biological research it is important we respect that ownership the investigator has because many times it is the investigator that appreciates the value of this sample. This sample is important to this investigator because uh they came out with a particular hypothesis. They wanted to answer a certain question. May be the question has not been answered and so it is important to respect that. The issue of moving with samples left to right it is another thing because this is where you come in as the IRB. | | | | | | | | | | | | | | | |  |
|  |  | | | | | | | | | | | | | | | |  |
|  | | | | | | | | | | | | 18 |  |  |  |  |  |
|  | Respondent: And you know these are things that we probably need to define. There has been a lot of talk about when Luc Montagnier was awarded the Nobel Prize with Professor Barré-Sinoussi for discovering HIV, in his Nobel Prize talk he talks about the safari research. This is the research where you know we have partners from the North coming in and all they do just pick samples and go out with them. | | | | | | | | | | | | | | | |  |
|  |  | | | | | | | | | | | | | | | |  |
|  | | | | | | | | | | | |  |  |  |  |  |  |
|  | Respondent: Yes. And this is quite disturbing in the sense that uh it presents the challenge: who owns the samples you know. Who owns the samples? And in the past you want to argue that yes and there have been situations like the one were were discussing about the ebola outbreak where really communities are vulnerablein West Africa. They need help, they need people to come with expertise and then people that come in with the expertise come and take advantage and literally go off with the samples. So I think that uh dealing with these kinds of things is quite difficult and it comes back to building local capacity. | | | | | | | | | | | | | | | |  |
|  |  | | | | | | | | | | | | | | | |  |
|  | | | | | | | | | | | | 20 |  |  |  |  |  |
|  | Respondent: Yes. And this is quite disturbing in the sense that uh it presents the challenge: who owns the samples you know. Who owns the samples? And in the past you want to argue that yes and there have been situations like the one were were discussing about the ebola outbreak where really communities are vulnerablein West Africa. They need help, they need people to come with expertise and then people that come in with the expertise come and take advantage and literally go off with the samples. So I think that uh dealing with these kinds of things is quite difficult and it comes back to building local capacity. | | | | | | | | | | | | | | | |  |
|  |  | | | | | | | | | | | | | | | |  |
|  | | | | | | | | | | | | 21 |  |  |  |  |  |
|  | Respondent: Local capacity in biomedical research is uh no one’s mandate. It is the mandate local governments. The local people need to do this. | | | | | | | | | | | | | | | |  |
|  |  | | | | | | | | | | | | | | | |  |
|  | | | **Files\\male\\IDI R11 male** | | | | | | | | | | | | | |  |
|  |  |  | Yes |  | person |  | 0.0700 |  | 7 | |  | | | | | |  |
|  | | |  |  |  |  |  |  |  | |  | | | | | | |
|  | | | | | | | | | | | | 1 |  |  |  |  |  |
|  | Respondent: I think, if it is done with in the guide lines (the required ethical guidelines and standards) it is good. because you just never know when the next opportunity arrives. well, it saves a lot of time, because coming back looking for participants to participate in a study with a question of interest. but also, it makes the entire research process easy because the we have samples readily available so it saves time | | | | | | | | | | | | | | | |  |
|  |  | | | | | | | | | | | | | | | |  |
|  | | | | | | | | | | | | 2 |  |  |  |  |  |
|  | Interviewer: ok. also the storage facilities for samples are scattered all over, freezers are in different locations. so in your opinion, how best should we handle these to get maximum utility from these samples and ensuring that they are well utilized with clear and strict quality control and quality assurance while these samples are being stored  Respondent: well, I think that whoever intends to store research samples, they should be certified or qualified to do as such. of course Bio- banks are the places of storage for these samples and I think to me, having a functional Bio- bank, to meet certain quality assurance levels for you to be able to do that. different collaborations n will try to come with ways of storing their samples. some are local, some are international, some are stored elsewhere and some are stored in country. but I think, the most important thing is that whoever is doing the research or collaboration storing samples should be able to meet the standards for setting up and maintaining the structure for Bio- banks | | | | | | | | | | | | | | | |  |
|  |  |  |  |  |  |  |  |  |  |  |  |  |  |  |  |  |  |
|  |  | | | | | | | | | | | | | | | |  |
| Reports\\Coding Summary By Code Report | | | | | | | | | | Page 37 of 40 | | | | | | | |
|  | | | | | | | | | | | | | | | | | |
|  | | | **Aggregate** |  | **Classification** |  | **Coverage** |  | **Number Of Coding References** | |  | **Reference Number** |  | **Coded By Initials** |  | **Modified On** |  |
|  | | | | | | | | | | | | | | | | | |
|  | | | | | | | | | | | | 3 |  |  |  |  |  |
|  | Interviewer: ok, thank you. so your opinion, who owns these biological samples and associated data?  Respondent: that is a good one. if you the sole entity running, you have the sole rights to the data. but if you in a collaboration, I think the data rights should be equally shared | | | | | | | | | | | | | | | |  |
|  |  | | | | | | | | | | | | | | | |  |
|  | | | | | | | | | | | | 4 |  |  |  |  |  |
|  | Interviewer: ok, thank you. so your opinion, who owns these biological samples and associated data?  Respondent: that is a good one. if you the sole entity running, you have the sole rights to the data. but if you in a collaboration, I think the data rights should be equally shared | | | | | | | | | | | | | | | |  |
|  |  | | | | | | | | | | | | | | | |  |
|  | | | | | | | | | | | | 5 |  |  |  |  |  |
|  | Respondent: umm. the participants are the owners but an institution is a custodian and it is given certain rights and permission on what to do with the samples. so in terms of ownership, I think it cuts across. I am I right on this? | | | | | | | | | | | | | | | |  |
|  |  | | | | | | | | | | | | | | | |  |
|  | | | | | | | | | | | | 6 |  |  |  |  |  |
|  | Respondent: umm. the participants are the owners but an institution is a custodian and it is given certain rights and permission on what to do with the samples. so in terms of ownership, I think it cuts across. I am I right on this? | | | | | | | | | | | | | | | |  |
|  |  | | | | | | | | | | | | | | | |  |
|  | | | | | | | | | | | | 7 |  |  |  |  |  |
|  | Respondent: but where there is capacity, I think there should be an opportunity to have these samples retained and analyzed here. but of course there are so many factors playing in the back ground, especially the funding | | | | | | | | | | | | | | | |  |
|  |  | | | | | | | | | | | | | | | |  |
|  | | | **Files\\male\\IDI R13 male** | | | | | | | | | | | | | |  |
|  |  |  | Yes |  |  |  | 0.2312 |  | 13 | |  | | | | | |  |
|  | | |  |  |  |  |  |  |  | |  | | | | | | |
|  | | | | | | | | | | | | 1 |  |  |  |  |  |
|  | Respondent: I think one of the biggest challenges we have is that we do not have very good repository for storing samples for future use and yet those samples could be used in the future. However, that should have also come out in the consent form, the consent form should clearly state that you are taking the sample but you could use it for some specific activity in the future | | | | | | | | | | | | | | | |  |
|  |  | | | | | | | | | | | | | | | |  |
|  | | | | | | | | | | | | 2 |  |  |  |  |  |
|  | Respondent: the purpose of the sample in the future should be specified. but in the case that you want to use for some other things, I would recommend that you go back and obtain consent for using them because otherwise, people could start using them for all sorts of things because you will have violated the trust this person will have given you over a period of time to be able to do that. so my thoughts are that about those samples are that we should find repositories but we should get clear informed consent from these people | | | | | | | | | | | | | | | |  |
|  |  | | | | | | | | | | | | | | | |  |
|  | | | | | | | | | | | | 3 |  |  |  |  |  |
|  | Respondent: the purpose of the sample in the future should be specified. but in the case that you want to use for some other things, I would recommend that you go back and obtain consent for using them because otherwise, people could start using them for all sorts of things because you will have violated the trust this person will have given you over a period of time to be able to do that. so my thoughts are that about those samples are that we should find repositories but we should get clear informed consent from these people | | | | | | | | | | | | | | | |  |
|  |  | | | | | | | | | | | | | | | |  |
|  | | | | | | | | | | | | 4 |  |  |  |  |  |
|  | Respondent: and may be if after 20 or so years of storing and you need to use them for some other thing, you should go back | | | | | | | | | | | | | | | |  |
|  |  | | | | | | | | | | | | | | | |  |
|  | | | | | | | | | | | | 5 |  |  |  |  |  |
|  | Interviewer: I see, but of course now, re-consenting needs a lot of resources especially with people migrating and others passing away, so if you are to re consent, you really need to have a lot of logistics. ok, this is very insightful. in your opinion who own biological samples and the associated data?  Respondent: the biological samples?  Interviewer: umm, is it the donor, is it the researcher, the sponsor, the institution?  Respondent: I would think that the researcher and the donor | | | | | | | | | | | | | | | |  |
|  |  | | | | | | | | | | | | | | | |  |
| Reports\\Coding Summary By Code Report | | | | | | | | | | Page 38 of 40 | | | | | | | |
|  | | | | | | | | | | | | | | | | | |
|  | | | **Aggregate** |  | **Classification** |  | **Coverage** |  | **Number Of Coding References** | |  | **Reference Number** |  | **Coded By Initials** |  | **Modified On** |  |
|  | | | | | | | | | | | | | | | | | |
|  | | | | | | | | | | | | 6 |  |  |  |  |  |
|  | Interviewer: I see, but of course now, re-consenting needs a lot of resources especially with people migrating and others passing away, so if you are to re consent, you really need to have a lot of logistics. ok, this is very insightful. in your opinion who own biological samples and the associated data?  Respondent: the biological samples?  Interviewer: umm, is it the donor, is it the researcher, the sponsor, the institution?  Respondent: I would think that the researcher and the donor | | | | | | | | | | | | | | | |  |
|  |  | | | | | | | | | | | | | | | |  |
|  | | | | | | | | | | | | 7 |  |  |  |  |  |
|  | Respondent: now, the donor (person who has given money to run the study) has consented to you that take his samples, they remain his samples because that is why you consent but before that you do what you specified to do in the consent form and in some circumstances in some centers after you have finished with the consenting during the period in which you are allowed to do that study, those samples are either stored. but beyond that you do not have any IRB approval to monitor how you are going to deal with the different processes involved in sample collection but at the time when the IRB has given you to do the study at that particular time. so in a way it is not the donor thus the donor should not own it, if a donor starts coming in that is now being conflicted (the donor is conflicted). it should be that mutual understanding between the researcher and the participant | | | | | | | | | | | | | | | |  |
|  |  |  |  |  |  |  |  |  |  |  |  |  |  |  |  |  |  |
|  |  | | | | | | | | | | | | | | | |  |
|  | | | | | | | | | | | | 8 |  |  |  |  |  |
|  | Respondent: now, the donor (person who has given money to run the study) has consented to you that take his samples, they remain his samples because that is why you consent but before that you do what you specified to do in the consent form and in some circumstances in some centers after you have finished with the consenting during the period in which you are allowed to do that study, those samples are either stored. but beyond that you do not have any IRB approval to monitor how you are going to deal with the different processes involved in sample collection but at the time when the IRB has given you to do the study at that particular time. so in a way it is not the donor thus the donor should not own it, if a donor starts coming in that is now being conflicted (the donor is conflicted). it should be that mutual understanding between the researcher and the participant | | | | | | | | | | | | | | | |  |
|  |  |  |  |  |  |  |  |  |  |  |  |  |  |  |  |  |  |
|  |  | | | | | | | | | | | | | | | |  |
|  | | | | | | | | | | | | 9 |  |  |  |  |  |
|  | Respondent: now, the donor (person who has given money to run the study) has consented to you that take his samples, they remain his samples because that is why you consent but before that you do what you specified to do in the consent form and in some circumstances in some centers after you have finished with the consenting during the period in which you are allowed to do that study, those samples are either stored. but beyond that you do not have any IRB approval to monitor how you are going to deal with the different processes involved in sample collection but at the time when the IRB has given you to do the study at that particular time. so in a way it is not the donor thus the donor should not own it, if a donor starts coming in that is now being conflicted (the donor is conflicted). it should be that mutual understanding between the researcher and the participant | | | | | | | | | | | | | | | |  |
|  |  |  |  |  |  |  |  |  |  |  |  |  |  |  |  |  |  |
|  |  | | | | | | | | | | | | | | | |  |
|  | | | | | | | | | | | | 10 |  |  |  |  |  |
|  | Respondent: now, the donor (person who has given money to run the study) has consented to you that take his samples, they remain his samples because that is why you consent but before that you do what you specified to do in the consent form and in some circumstances in some centers after you have finished with the consenting during the period in which you are allowed to do that study, those samples are either stored. but beyond that you do not have any IRB approval to monitor how you are going to deal with the different processes involved in sample collection but at the time when the IRB has given you to do the study at that particular time. so in a way it is not the donor thus the donor should not own it, if a donor starts coming in that is now being conflicted (the donor is conflicted). it should be that mutual understanding between the researcher and the participant | | | | | | | | | | | | | | | |  |
|  |  |  |  |  |  |  |  |  |  |  |  |  |  |  |  |  |  |
|  |  | | | | | | | | | | | | | | | |  |
|  | | | | | | | | | | | | 11 |  |  |  |  |  |
|  | Interviewer: ok, do you know what is in the national guidelines?  Respondent: I am not sure but  Interviewer: in our national guidelines, the sample donor(participant) is the owner then the institution is just a custodian  Respondent: umm  Interviewer: that is why, when a donor(participant) says “I am tired of this study I want to withdraw, they have a right to do so because they still have rights”. so what do you think of the export of samples and sharing of samples with foreign collaborators?  Respondent: I am very concerned about these issues of taking samples outside the country  Interviewer: umm, you are not alone | | | | | | | | | | | | | | | |  |
|  |  |  |  |  |  |  |  |  |  |  |  |  |  |  |  |  |  |
| Reports\\Coding Summary By Code Report | | | | | | | | | | Page 39 of 40 | | | | | | | |
|  | | | | | | | | | | | | | | | | | |
|  | | | **Aggregate** |  | **Classification** |  | **Coverage** |  | **Number Of Coding References** | |  | **Reference Number** |  | **Coded By Initials** |  | **Modified On** |  |
|  | | | | | | | | | | | | | | | | | |
|  | | | | | | | | | | | | 12 |  |  |  |  |  |
|  | Respondent: I am very concerned about these issues of taking samples outside the country  Interviewer: umm, you are not alone  Respondent: it is a security problem as well; it is much more than just taking people’s samples. anybody can use it for any other thing and for me my county has been relaxed on this matter and peopl  Respondent: I think those are very cardinal and in this country, many people have been benefitting from this kind of thing and we need to take a closer look at it and make it something which is well protected and guarded. and for anything which goes out, a return request should be made once the process is done. and in fact in addition to that, I would propose that local researcher should visit where they are taking the samples and participate in the process of the analysis so that the moment the analysis is finished, he/ she can see how to have the samples back  Interviewer: sure, that also helps in technology and knowledge transfer (capacity building)  Respondent: yeah, that is right  Interviewer: so, now basing on your opinion about this, how should this sample export be regulated? because you now we have a problem with rege just come in and take our samples. what they do with it there, we do not have control over it and even when they have done what they really obtained the consent for, they never return them. and in addition to that, even if they are returned, where are they stored? so for me I think it is something that we need to carefully look at, we need to strengthen it and we need to make it hard to take samples out of this country | | | | | | | | | | | | | | | |  |
|  |  |  |  |  |  |  |  |  |  |  |  |  |  |  |  |  |  |
|  |  |  |  |  |  |  |  |  |  |  |  |  |  |  |  |  |  |
|  |  | | | | | | | | | | | | | | | |  |
|  | | | | | | | | | | | | 13 |  |  |  |  |  |
|  | Interviewer: ok, you know there is one called National Health Laboratory services at Butabika. they are trying to build a repository where by many of these samples can be kept and for the future, they equip the lab so that most of the required tests can be done here. so that is work in progress and I hope it successful | | | | | | | | | | | | | | | |  |

| Perceptions of members of RECs | | | | | | | | | | | | | | | | | |
| --- | --- | --- | --- | --- | --- | --- | --- | --- | --- | --- | --- | --- | --- | --- | --- | --- | --- |
| 4/27/2021 2:26 PM | | | | | | | | | | | | | | | | | |
|  | | | **Aggregate** |  | **Classification** |  | **Coverage** |  | **Number Of Coding References** | |  | **Reference Number** |  | **Coded By Initials** |  | **Modified On** |  |
| **Node** | | | | | | | | | | | | | | | | |  |
|  | **Nodes\\B. Ethics: benefit sharing, exploitation, commercialisation** | | | | | | | | | | | | | | | |  |
|  | | **Document** | | | | | | | | | | | | | | |  |
|  | | | **Files\\Community\\female\\IDI Transcript 014 female** | | | | | | | | | | | | | |  |
|  |  |  | Yes |  | Community |  | 0.0514 |  | 3 | |  | | | | | |  |
|  | | |  |  |  |  |  |  |  | |  | | | | | | |
|  | | | | | | | | | | | |  |  |  |  |  |  |
|  | R: You know you talked about ethical, (I: hmm), I have actually been bringing in the study then we study (I: hmm) and then we give in our input in this kind of study they are going to study on a gene so basically that’s where we are but as society in terms of appreciation of the genetics, it’s something that’s hard because someone feels that you want to take my genes to who (I: hmm) to who? | | | | | | | | | | | | | | | |  |
|  |  | | | | | | | | | | | | | | | |  |
|  | | | | | | | | | | | |  |  |  |  |  |  |
|  | So, we are like, these people want to take our things but for what (I: hmm, but for what) much as we are saying that it’s science, science so what in terms of real world (I: hmm, yes) what is real science? When you take of taking my genes to somewhere and what’s funny you always carry them as sample and I really don’t know as a community person what will happen apart from you telling me that it’s science (I: hmm) but what will happen in the real world may be after 40 or 50 years are you going to get different genes and make different people I’m really understanding that (I: hmm) so that’s what I’m like seeing (I: hmm). | | | | | | | | | | | | | | | |  |
|  |  | | | | | | | | | | | | | | | |  |
|  | | | | | | | | | | | |  |  |  |  |  |  |
|  | So I feel that my ethical team is competent enough (I: hmm) and it really helps to break it down so that I really understand, if I don’t understand I usually tell my them that I have not understood, I don’t agree because that’s me (I: hmm, yes) I’m a community representative, I’m not one I’m representing people so if I don’t understand, I don’t agree (I: hmm), so it’s their role to explain to me whatever and I understand it well otherwise I don’t agree but I feel they are competent enough and they explain they break it down that I really understand (I: hmm). | | | | | | | | | | | | | | | |  |
|  |  | | | | | | | | | | | | | | | |  |
|  | | | **Files\\Community\\male\\IDI Transcript 008 male** | | | | | | | | | | | | | |  |
|  |  |  | Yes |  |  |  | 0.0322 |  | 1 | |  | | | | | |  |
|  | | |  |  |  |  |  |  |  | |  | | | | | | |
|  | | | | | | | | | | | |  |  |  |  |  |  |
|  | For me I would think that the researcher may be really looking at a certain benefit or reducing a certain risk, and if one went out to do a research about genetics, maybe that is why but given that these days there is a lot of struggling, whose child is this, whose child is the other, and for us who have been to school we are close to the hospital, I receive phone calls (Int: umm), somebody is saying, “you know this one is doubting this child, what do you help us to do? Then I say, “you go to this laboratory. But may be in that area, it is simply to clear doubts among the people or to make people fairy sure at a social level because even about their origins, and their relationships and lineages uh that is concerning human beings. Yeah, that is what I would think otherwise to me it looks a complicated, a complex research to a lay man. | | | | | | | | | | | | | | | |  |
|  |  | | | | | | | | | | | | | | | |  |
|  | | | | | | | | | | | | | | | | | |
| Reports\\Coding Summary By Code Report | | | | | | | | | | Page 1 of 16 | | | | | | | |
|  | | | **Aggregate** |  | **Classification** |  | **Coverage** |  | **Number Of Coding References** | |  | **Reference Number** |  | **Coded By Initials** |  | **Modified On** |  |
|  | | | **Files\\Community\\male\\IDI Transcript 011 male** | | | | | | | | | | | | | |  |
|  |  |  | Yes |  |  |  | 0.0221 |  | 2 | |  | | | | | |  |
|  | | |  |  |  |  |  |  |  | |  | | | | | | |
|  | | | | | | | | | | | |  |  |  |  |  |  |
|  | It all involves being ethical, protecting participants, doing the research properly, producing the results and giving them to the community. | | | | | | | | | | | | | | | |  |
|  |  | | | | | | | | | | | | | | | |  |
|  | | | | | | | | | | | |  |  |  |  |  |  |
|  | what we call ethical considerations of research must be followed. And should they be faulted individuals should be held accountable. That is the best way. So, the safest way is to add a clause in our research, that should a researcher fault guidelines, particularly ethical guidelines including even science as well. Because if science guidelines say that you put 2 millimeters [of blood] and you put 4, you are faulting the guidelines. And also, you are doing a wrong science and you are being unethical.  So, my point is that community can be safeguarded in two ways: one, a clause be put on the researchers that flouting ethical guidelines, flouting science guidelines, you are punishable. So that anybody doing research is aware that if I was supposed to take 10 samples and you take 15 samples, you are faulting guidelines. If you are supposed to monitor every week, then for you you monitor once in a month, eventually some people die because of…………. So, faulting ethical and science guidelines people should be held accountable. | | | | | | | | | | | | | | | |  |
|  |  |  |  |  |  |  |  |  |  |  |  |  |  |  |  |  |  |
|  |  | | | | | | | | | | | | | | | |  |
|  | | | **Files\\Community\\male\\IDI Transcript 015 male** | | | | | | | | | | | | | |  |
|  |  |  | Yes |  |  |  | 0.0770 |  | 5 | |  | | | | | |  |
|  | | |  |  |  |  |  |  |  | |  | | | | | | |
|  | | | | | | | | | | | |  |  |  |  |  |  |
|  | The other part was? (I: ethics of this genomic research). Now that one is a bit detailed and it gets complicated. Because when we get to the ethics we look at ahh we want to see the process of consenting. we want to see if someone is getting involved in something they understand because ethically for you to engage someone in any kind of research, they must really know. | | | | | | | | | | | | | | | |  |
|  |  | | | | | | | | | | | | | | | |  |
|  | | | | | | | | | | | |  |  |  |  |  |  |
|  | So, for me ethically one of the things for someone in any kind of research, they must really know. One of the things that will take my attention is does this person know because there is a lot of English here. Every time I sit on the IRB, I keep reminding people that for me I am representing that old woman in Kyegegwa where I work that refugee who doesn’t know sometimes the language you are using. So ethically I would want to see the terms for example genetics, genomics. Like I would like to see this broken down so that this person participates. So, for me if all those things are taken care of them ethically i would know we are on the right track. | | | | | | | | | | | | | | | |  |
|  |  | | | | | | | | | | | | | | | |  |
|  | | | | | | | | | | | |  |  |  |  |  |  |
|  | I know when you take off these sample, for now the reason for taking off these samples could be something very small but as you keep the samples, and something comes giving them off could be something very small. You know science is evolving, an idea comes people can quickly go to those samples and then use them. And like you are saying you see how you create another Deborah and Deborah has no idea. For me this is somebody who has no idea, and this is someone who ahh for me this is a big issue and I don’t know how the gates can be made very tight. because I am also aware that the moment  a participant or myself i give any type of sample. I know I may sign certain things I am consenting to but I know the moment just like when I give  you information, you may promise me certain things but there is a level to which i cannot go. For me ethically that would be my big concern. what are we going to do to be sure that what they said because for me creating another one will not be a big problem if i know what is happening or if this participant who consented knows what you are going to do and the implication of what will happen there after. | | | | | | | | | | | | | | | |  |
|  |  |  |  |  |  |  |  |  |  |  |  |  |  |  |  |  |  |
|  |  | | | | | | | | | | | | | | | |  |
|  | | | | | | | | | | | |  |  |  |  |  |  |
|  | I know when you take off these sample, for now the reason for taking off these samples could be something very small but as you keep the samples, and something comes giving them off could be something very small. You know science is evolving, an idea comes people can quickly go to those samples and then use them. And like you are saying you see how you create another Deborah and Deborah has no idea. For me this is somebody who has no idea, and this is someone who ahh for me this is a big issue and I don’t know how the gates can be made very tight. because I am also aware that the moment  a participant or myself i give any type of sample. I know I may sign certain things I am consenting to but I know the moment just like when I give  you information, you may promise me certain things but there is a level to which i cannot go. For me ethically that would be my big concern. what are we going to do to be sure that what they said because for me creating another one will not be a big problem if i know what is happening or if this participant who consented knows what you are going to do and the implication of what will happen there after. | | | | | | | | | | | | | | | |  |
|  |  |  |  |  |  |  |  |  |  |  |  |  |  |  |  |  |  |
|  |  | | | | | | | | | | | | | | | |  |
| Reports\\Coding Summary By Code Report | | | | | | | | | | Page 2 of 16 | | | | | | | |
|  | | | **Aggregate** |  | **Classification** |  | **Coverage** |  | **Number Of Coding References** | |  | **Reference Number** |  | **Coded By Initials** |  | **Modified On** |  |
|  | | | | | | | | | | | | | | | | | |
|  | | | | | | | | | | | |  |  |  |  |  |  |
|  | I know when you take off these sample, for now the reason for taking off these samples could be something very small but as you keep the samples, and something comes giving them off could be something very small. You know science is evolving, an idea comes people can quickly go to those samples and then use them. And like you are saying you see how you create another Deborah and Deborah has no idea. For me this is somebody who has no idea, and this is someone who ahh for me this is a big issue and I don’t know how the gates can be made very tight. because I am also aware that the moment  a participant or myself i give any type of sample. I know I may sign certain things I am consenting to but I know the moment just like when I give  you information, you may promise me certain things but there is a level to which i cannot go. For me ethically that would be my big concern. what are we going to do to be sure that what they said because for me creating another one will not be a big problem if i know what is happening or if this participant who consented knows what you are going to do and the implication of what will happen there after. | | | | | | | | | | | | | | | |  |
|  |  |  |  |  |  |  |  |  |  |  |  |  |  |  |  |  |  |
|  |  | | | | | | | | | | | | | | | |  |
|  | | | **Files\\Researchers\\female\\IDI Transcript 004 female** | | | | | | | | | | | | | |  |
|  |  |  | Yes |  |  |  | 0.0734 |  | 3 | |  | | | | | |  |
|  | | |  |  |  |  |  |  |  | |  | | | | | | |
|  | | | | | | | | | | | |  |  |  |  |  |  |
|  | Resp: I think the main issues have been around dissemination. That is where I have had people with conflicting grounds on whether they should disclose or not disclose, or if whatever came out is not what they anticipated. And they don’t know even how to disclose to the community. And also the implications of your findings. | | | | | | | | | | | | | | | |  |
|  |  | | | | | | | | | | | | | | | |  |
|  | | | | | | | | | | | |  |  |  |  |  |  |
|  | Resp: There are many things it might help us to explain. There are things that could be going on in communities or within families but they are unexplainable and they might come out to light if they are genetic findings to do with them. We know where diseases are concerned, some are rendered protective by having some genetic makeup and some might be susceptible or they are at more risk of a certain disease because of a certain genetic makeup. So those who have advantages might feel superior while those who have the disadvantages may feel, not exactly let down but the implication also of propagating that kind of information may not be good. | | | | | | | | | | | | | | | |  |
|  |  | | | | | | | | | | | | | | | |  |
|  | | | | | | | | | | | |  |  |  |  |  |  |
|  | Resp: For the risks, it also depends. Some genetic findings might say; down the road when you are 80 years something might happen to you because of this finding. But right now you are 40 years. So people might even fall sick before they get to eighty. If I know that there is something negative that is coming up, I might worry even when it could not manifest in me but I have been told that maybe it runs in the family. It can have deleterious outcomes based on how you handle it because disclosing to me, and you are not giving any follow up to see what happens along/down the road might also be very risky. So if you tell me I have this, what next? You must have that answer.  Int: So you would rather let sleeping dogs lie instead of reawakening them when you don’t have food for them.  Resp: Packaging has to be extremely thought about. | | | | | | | | | | | | | | | |  |
|  |  |  |  |  |  |  |  |  |  |  |  |  |  |  |  |  |  |
|  |  | | | | | | | | | | | | | | | |  |
|  | | | **Files\\Researchers\\female\\IDI Transcript 007 female** | | | | | | | | | | | | | |  |
|  |  |  | Yes |  |  |  | 0.0238 |  | 2 | |  | | | | | |  |
|  | | |  |  |  |  |  |  |  | |  | | | | | | |
|  | | | | | | | | | | | |  |  |  |  |  |  |
|  | Respondent: the time I have served on the research and ethics committee, the main issue has been around storing and indefinitely using somebody’s genetic material and the requirement of the investigators for the research participants to sign giving authority to investigators to do blind ended research on their stored specimen and at the point of the consent it is not clear what else is going to be done in future. | | | | | | | | | | | | | | | |  |
|  |  | | | | | | | | | | | | | | | |  |
|  | | | | | | | | | | | |  |  |  |  |  |  |
|  | Respondent: quite frankly, no! The benefits at this point, from the study (ies) that I have reviewed have involved benefits a raising from the research that the investigator is doing now, but the genetics component of it usually (from the protocols that I have reviewed) the participant is not at this point benefitting  Interviewer: so there are no risk benefits?  Respondent: no | | | | | | | | | | | | | | | |  |
|  |  | | | | | | | | | | | | | | | |  |
|  | | | | | | | | | | | | | | | | | |
| Reports\\Coding Summary By Code Report | | | | | | | | | | Page 3 of 16 | | | | | | | |
|  | | | **Aggregate** |  | **Classification** |  | **Coverage** |  | **Number Of Coding References** | |  | **Reference Number** |  | **Coded By Initials** |  | **Modified On** |  |
|  | | | **Files\\Researchers\\female\\IDI Transcript 010 female** | | | | | | | | | | | | | |  |
|  |  |  | Yes |  |  |  | 0.0323 |  | 1 | |  | | | | | |  |
|  | | |  |  |  |  |  |  |  | |  | | | | | | |
|  | | | | | | | | | | | |  |  |  |  |  |  |
|  | Respondent: yes, what I know, if you are carrying out genetic research, the participant has to consent more than once, like for example the participant will consent for participating in the research, then also will consent for sample storage and of course if you want to re-use it, at least the participant must be aware and must consent for that | | | | | | | | | | | | | | | |  |
|  |  | | | | | | | | | | | | | | | |  |
|  | | | **Files\\Researchers\\female\\IDI Transcript 013 female** | | | | | | | | | | | | | |  |
|  |  |  | Yes |  |  |  | 0.0473 |  | 2 | |  | | | | | |  |
|  | | |  |  |  |  |  |  |  | |  | | | | | | |
|  | | | | | | | | | | | |  |  |  |  |  |  |
|  | Then on the ethical aspect, again that bit can apply ethically most likely at the time of the proposal development for conditions which are treatable. They are likely to ask that what are the benefits for the research participant? Because it is like when we do ethically we are doing the study. Of course, going through the ethical principles this participant must have voluntarily been allowed to participate in the study and since we talk of issues of ensuring that you minimize the harm and you try to enhance the welfare of the study participant. So, from the ethical principles, so when you are studying a condition where there is a possibility of treatment and especially cure and this participant being better ethically you will kind of be bound (I: hmm). And yet a number of researchers some researches may have limited budget not to have insurance issues to cater for that even if it is not an intervention study. so there those ethical issues which come on board. And then the other ethical issue which can come on board is so if this person is identified with this kind of gene, if you find it what does it mean? When you are giving him the results and then when it is genetical, it implies it is no longer a one-person story. It implies people are affected. So, what are the boundaries? What can the researcher cover? So those kinds of issues can come up and bring about ethical and legal challenges especially on the side of the researcher and more so if this person is informed about their rights. | | | | | | | | | | | | | | | |  |
|  |  |  |  |  |  |  |  |  |  |  |  |  |  |  |  |  |  |
|  |  | | | | | | | | | | | | | | | |  |
|  | | | | | | | | | | | |  |  |  |  |  |  |
|  | Then on the ethical aspect, again that bit can apply ethically most likely at the time of the proposal development for conditions which are treatable. They are likely to ask that what are the benefits for the research participant? Because it is like when we do ethically we are doing the study. Of course, going through the ethical principles this participant must have voluntarily been allowed to participate in the study and since we talk of issues of ensuring that you minimize the harm and you try to enhance the welfare of the study participant. So, from the ethical principles, so when you are studying a condition where there is a possibility of treatment and especially cure and this participant being better ethically you will kind of be bound (I: hmm). And yet a number of researchers some researches may have limited budget not to have insurance issues to cater for that even if it is not an intervention study. so there those ethical issues which come on board. And then the other ethical issue which can come on board is so if this person is identified with this kind of gene, if you find it what does it mean? When your giving him the results and then when it is genetical, it implies it is no longer a one-person story. It implies people are affected. So, what are the boundaries? What can the researcher cover? So those kinds of issues can come up and bring about ethical and legal challenges especially on the side of the researcher and more so if this person is informed about their rights. | | | | | | | | | | | | | | | |  |
|  |  |  |  |  |  |  |  |  |  |  |  |  |  |  |  |  |  |
|  |  | | | | | | | | | | | | | | | |  |
|  | | | **Files\\Researchers\\male\\IDI Trancript 006 male** | | | | | | | | | | | | | |  |
|  |  |  | Yes |  |  |  | 0.0309 |  | 1 | |  | | | | | |  |
|  | | |  |  |  |  |  |  |  | |  | | | | | | |
|  | | | | | | | | | | | |  |  |  |  |  |  |
|  | Respondent: i guess it is mainly that. I do not think there is any other main ethical issue but sometimes when we collect samples, for most of the genetic studies, they request to store samples and conduct further studies as needed and sometimes the ethical boards have no control on those samples and different laboratories can work on different samples and experiments like those about gene deletion can be done or performed and with such practices from the legal angle, you can introduce mutations that can affect different groups of people or populations and you can cause diseases. so it is complex | | | | | | | | | | | | | | | |  |
|  |  | | | | | | | | | | | | | | | |  |
|  | | | | | | | | | | | | | | | | | |
| Reports\\Coding Summary By Code Report | | | | | | | | | | Page 4 of 16 | | | | | | | |
|  | | | | | | | | | | | | | | | | | |
|  | | | **Aggregate** |  | **Classification** |  | **Coverage** |  | **Number Of Coding References** | |  | **Reference Number** |  | **Coded By Initials** |  | **Modified On** |  |
|  | | | **Files\\Researchers\\male\\IDI Transcript 001 male** | | | | | | | | | | | | | |  |
|  |  |  | Yes |  |  |  | 0.0742 |  | 6 | |  | | | | | |  |
|  | | |  |  |  |  |  |  |  | |  | | | | | | |
|  | | | | | | | | | | | |  |  |  |  |  |  |
|  | Respondent: I think one of the ethical issues is in regard to consenting because when you are talking about genetics, it means that you are no longer focusing on the individual per say, but several generations. because genetics really transcends from individuals- families, to communities then generations. So, when you are consenting some body, for the information that can be attributed from one person to the rest of the family and not part of the study, that is a serious ethical issue. Then the other thing is that genetic research could reveal things that are not at that time presentable to the individual. implying that the implication of genetic research may be way and out of the mind and eyes of individual that is participating in research. the third issue could be that the outcome of genetic research is something that is not clearly appreciated. if I provide my genetic information and something develops out of it, how I am supposed to accrue of the reality that comes from such research? | | | | | | | | | | | | | | | |  |
|  |  |  |  |  |  |  |  |  |  |  |  |  |  |  |  |  |  |
|  |  | | | | | | | | | | | | | | | |  |
|  | | | | | | | | | | | |  |  |  |  |  |  |
|  | Respondent: I think one of the ethical issues is in regard to consenting because when you are talking about genetics, it means that you are no longer focusing on the individual per say, but several generations. because genetics really transcends from individuals- families, to communities then generations. so, when you are consenting some body, for the information that can be attributed from one person to the rest of the family and not part of the study, that is a serious ethical issue. then the other thing is that genetic research could reveal things that are not at that time presentable to the individual. implying that the implication of genetic research may be way and out of the mind and eyes of individual that is participating in research. the third issue could be that the outcome of genetic research is something that is not clearly appreciated. if I provide my genetic information and something develops out of it, how I am supposed to accrue of the reality that comes from such research?  Interviewer: so basically, that is about sharing of benefits  Respondent: sharing of benefits, yeah | | | | | | | | | | | | | | | |  |
|  |  |  |  |  |  |  |  |  |  |  |  |  |  |  |  |  |  |
|  |  | | | | | | | | | | | | | | | |  |
|  | | | | | | | | | | | |  |  |  |  |  |  |
|  | Respondent: I think one of the ethical issues is in regard to consenting because when you are talking about genetics, it means that you are no longer focusing on the individual per say, but several generations. because genetics really transcends from individuals- families, to communities then generations. so, when you are consenting some body, for the information that can be attributed from one person to the rest of the family and not part of the study, that is a serious ethical issue. then the other thing is that genetic research could reveal things that are not at that time presentable to the individual. implying that the implication of genetic research may be way and out of the mind and eyes of individual that is participating in research. the third issue could be that the outcome of genetic research is something that is not clearly appreciated. if I provide my genetic information and something develops out of it, how I am supposed to accrue of the reality that comes from such research? | | | | | | | | | | | | | | | |  |
|  |  |  |  |  |  |  |  |  |  |  |  |  |  |  |  |  |  |
|  |  | | | | | | | | | | | | | | | |  |
|  | | | | | | | | | | | |  |  |  |  |  |  |
|  | Respondent: thirdly, the fact that genetic research is high tech research, capacity for an ordinary man to understand it is difficult. so however much you would want to have informed consent, there is a fact that there are things you can never appropriately explain to the participant  Interviewer: later alone the researcher himself may not understand(laughs) | | | | | | | | | | | | | | | |  |
|  |  | | | | | | | | | | | | | | | |  |
|  | | | | | | | | | | | |  |  |  |  |  |  |
|  | Respondent: thirdly, the fact that genetic research is high tech research, capacity for an ordinary man to understand it is difficult. so however much you would want to have informed consent, there is a fact that there are things you can never appropriately explain to the participant  Interviewer: later alone the researcher himself may not understand(laughs) | | | | | | | | | | | | | | | |  |
|  |  | | | | | | | | | | | | | | | |  |
|  | | | | | | | | | | | |  |  |  |  |  |  |
|  | Respondent: the risks are really the ethical issues, actually one of the risks that is so common is the fact that in situations when there is loss in confidentiality and someone’s genetics is exposed, to other people, then there may be chances of getting traumatized on the side of the participant. There are examples that have happened, the recent story is where a study was done in the Damascus where they were trying to compare peoples’ abilities to be entrepreneurs to genetics, and they realized that actually damascenes are poor entrepreneurs because of their genetic makeup which lead them to abandon their businesses because they are useless | | | | | | | | | | | | | | | |  |
|  |  | | | | | | | | | | | | | | | |  |
|  | | | **Files\\Researchers\\male\\IDI Transcript 002 male** | | | | | | | | | | | | | |  |
|  |  |  | Yes |  |  |  | 0.2261 |  | 30 | |  | | | | | |  |
|  | | |  |  |  |  |  |  |  | |  | | | | | | |
|  | | | | | | | | | | | |  |  |  |  |  |  |
|  | R: Some of the critical issues on ethics ahh impact on issues to do with informed consent, understanding of genetic concepts. Basically, what happens is that our population is ahh both the scientific population and the (I: general population) the community. | | | | | | | | | | | | | | | |  |
|  |  | | | | | | | | | | | | | | | |  |
| Reports\\Coding Summary By Code Report | | | | | | | | | | Page 5 of 16 | | | | | | | |
|  | | | | | | | | | | | | | | | | | |
|  | | | **Aggregate** |  | **Classification** |  | **Coverage** |  | **Number Of Coding References** | |  | **Reference Number** |  | **Coded By Initials** |  | **Modified On** |  |
|  | | | | | | | | | | | | | | | | | |
|  | | | | | | | | | | | |  |  |  |  |  |  |
|  | R: Some of the critical issues on ethics ahh impact on issues to do with informed consent, understanding of genetic concepts. Basically, what happens is that our population is ahh both the scientific population and the (I: general population) the community. | | | | | | | | | | | | | | | |  |
|  |  | | | | | | | | | | | | | | | |  |
|  | | | | | | | | | | | |  |  |  |  |  |  |
|  | R: Some of the critical issues on ethics ahh impact on issues to do with informed consent, understanding of genetic concepts. Basically, what happens is that our population is ahh both the scientific population and the (I: general population) the community. We call them genetic illiterates or genomic illiterates. We do not we have not yet acquired an adequate degree of basic understanding of genetics and genomics | | | | | | | | | | | | | | | |  |
|  |  | | | | | | | | | | | | | | | |  |
|  | | | | | | | | | | | |  |  |  |  |  |  |
|  | So, things are strange, things are for real, things are coded | | | | | | | | | | | | | | | |  |
|  |  | | | | | | | | | | | | | | | |  |
|  | | | | | | | | | | | |  |  |  |  |  |  |
|  | We don’t have local ahh our local understanding of heredity is different from the scientific understanding of heredity | | | | | | | | | | | | | | | |  |
|  |  | | | | | | | | | | | | | | | |  |
|  | | | | | | | | | | | |  |  |  |  |  |  |
|  | We don’t have local ahh our local understanding of heredity is different from the scientific understanding of heredity | | | | | | | | | | | | | | | |  |
|  |  | | | | | | | | | | | | | | | |  |
|  | | | | | | | | | | | |  |  |  |  |  |  |
|  | Ahh and so as a culture, as a people we don’t understand heredity in terms of the basic science aspects. | | | | | | | | | | | | | | | |  |
|  |  | | | | | | | | | | | | | | | |  |
|  | | | | | | | | | | | |  |  |  |  |  |  |
|  | So, the conceptualization of ahh lets says issues to do with ahh the fact that a person’s identity, a person’s height, a person’s colour, a person’s physical chemical characteristics are determined by genes. Its not the concept that we have about the future of people. | | | | | | | | | | | | | | | |  |
|  |  | | | | | | | | | | | | | | | |  |
|  | | | | | | | | | | | |  |  |  |  |  |  |
|  | So, to me it means therefore right from the beginning that informed consent is going to be a problem. | | | | | | | | | | | | | | | |  |
|  |  | | | | | | | | | | | | | | | |  |
|  | | | | | | | | | | | |  |  |  |  |  |  |
|  | Ahh people don’t have the basic knowledge including the scientists in the area about genomics then informed consent is going to be a problem, problematic. We don’t have a word for gene, we don’t have a word for genetics, don’t have a word for any of those. And of course, genetic ahh genomic science has moved so fast that different terms come in every other time. | | | | | | | | | | | | | | | |  |
|  |  | | | | | | | | | | | | | | | |  |
|  | | | | | | | | | | | |  |  |  |  |  |  |
|  | Ahh people don’t have the basic knowledge including the scientists in the area about genomics then informed consent is going to be a problem, problematic. We don’t have a word for gene, we don’t have a word for genetics, don’t have a word for any of those. And of course, genetic ahh genomic science has moved so fast that different terms come in every other time. | | | | | | | | | | | | | | | |  |
|  |  | | | | | | | | | | | | | | | |  |
|  | | | | | | | | | | | |  |  |  |  |  |  |
|  | Ahh people don’t have the basic knowledge including the scientists in the area about genomics then informed consent is going to be a problem, problematic. We don’t have a word for gene, we don’t have a word for genetics, don’t have a word for any of those. And of course, genetic ahh genomic science has moved so fast that different terms come in every other time. | | | | | | | | | | | | | | | |  |
|  |  | | | | | | | | | | | | | | | |  |
|  | | | | | | | | | | | |  |  |  |  |  |  |
|  | We can’t keep pace you know how are we going to translate words like alleles? How are we going to translate penetrance? Its almost impossible that and yet if for example you are talking about risk, probabilities (I: coughs) of inheritance. How do you bring this to a person who doesn’t understand? It’s a very big problem with informed consent (I: hmm). Ahh translations of informed consents from English to the local languages (I: hmm) are a very big problem yeah for some of us who review them. It looks a laughable project to say that actually your translating a consent that you know is talking about genetic terminologies in a local language and it even makes one laugh. When you try a back translation, you may find that the back translation may be talking about building houses because you have to bring in concepts like building blocks (I: yes), bricks, like joints. It totally looks like totally a different thing. | | | | | | | | | | | | | | | |  |
|  |  |  |  |  |  |  |  |  |  |  |  |  |  |  |  |  |  |
|  |  | | | | | | | | | | | | | | | |  |
| Reports\\Coding Summary By Code Report | | | | | | | | | | Page 6 of 16 | | | | | | | |
|  | | | | | | | | | | | | | | | | | |
|  | | | **Aggregate** |  | **Classification** |  | **Coverage** |  | **Number Of Coding References** | |  | **Reference Number** |  | **Coded By Initials** |  | **Modified On** |  |
|  | | | | | | | | | | | | | | | | | |
|  | | | | | | | | | | | |  |  |  |  |  |  |
|  | How do you bring this to a person who doesn’t understand? It’s a very big problem with informed consent (I: hmm). Ahh translations of informed consents from English to the local languages (I: hmm) are a very big problem yeah for some of us who review them. It looks a laughable project to say that actually you are translating a consent that you know is talking about genetic terminologies in a local language and it even makes one laugh. When you try a back translation, you may find that the back translation may be talking about building houses because you have to bring in concepts like building blocks (I: yes), bricks, like joints. It totally looks like totally a different thing. Ahh then the other big issue that comes in the consent process, we have the big issue of ahh you know the problem with genomics is that it is a science that sucks in an individual with his or her community | | | | | | | | | | | | | | | |  |
|  |  |  |  |  |  |  |  |  |  |  |  |  |  |  |  |  |  |
|  |  | | | | | | | | | | | | | | | |  |
|  | | | | | | | | | | | |  |  |  |  |  |  |
|  | How do you bring this to a person who doesn’t understand? It’s a very big problem with informed consent (I: hmm). Ahh translations of informed consents from English to the local languages (I: hmm) are a very big problem yeah for some of us who review them. It looks a laughable project to say that actually your translating a consent that you know is talking about genetic terminologies in a local language and it even makes one laugh. When you try a back translation, you may find that the back translation may be talking about building houses because you have to bring in concepts like building blocks (I: yes), bricks, like joints. It totally looks like totally a different thing. | | | | | | | | | | | | | | | |  |
|  |  | | | | | | | | | | | | | | | |  |
|  | | | | | | | | | | | |  |  |  |  |  |  |
|  | Most of let’s say the human rights ethos, ethics is based on the rights of an individual. The ethical guidelines that we have in the country are based on the individual. | | | | | | | | | | | | | | | |  |
|  |  | | | | | | | | | | | | | | | |  |
|  | | | | | | | | | | | |  |  |  |  |  |  |
|  | Ahh how do you determine risk for an individual being involved in a study that has a risk of community stigma (I: hmm)? Who is determining the risk? Is it the individual or is it the community which doesn’t even know it is being studied by proxy? (I: or is it the researcher?) or is it the researcher? So, it has become a very big ethical issue. | | | | | | | | | | | | | | | |  |
|  |  | | | | | | | | | | | | | | | |  |
|  | | | | | | | | | | | |  |  |  |  |  |  |
|  | Who is determining the risk? Is it the individual or is it the community which doesn’t even know it is being studied by proxy? (I: or is it the researcher?) or is it the researcher? So, it has become a very big ethical issue. | | | | | | | | | | | | | | | |  |
|  |  | | | | | | | | | | | | | | | |  |
|  | | | | | | | | | | | |  |  |  |  |  |  |
|  | The other big ethical issue I see here is on risk benefit analysis, okay normally risk benefit analysis is done by many players. The investigator, the research ethics, the regulators ahh the research participants and even the research community. But you see risk benefit analysis requires one to have an understanding of the risks, measurements, their frequencies and probabilities and understanding of the benefits in the same way and then carrying out a risk benefit analysis objectively or subjectively. Genomics and genetics by nature have a certain degree of uncertainty around them. | | | | | | | | | | | | | | | |  |
|  |  | | | | | | | | | | | | | | | |  |
|  | | | | | | | | | | | |  |  |  |  |  |  |
|  | Genomics and genetics by nature have a certain degree of uncertainty around them. So, for example if we talk about ahh genes have different expressivities, expressions. Ahh genes for pheochromocytoma and they manifest at 40 and this person is let’s say 10 years of age, how do you interpret that kind of remote risk? | | | | | | | | | | | | | | | |  |
|  |  | | | | | | | | | | | | | | | |  |
|  | | | | | | | | | | | |  |  |  |  |  |  |
|  | So, for example if we talk about ahh genes have different expressivities, expressions. Ahh genes for pheochromocytoma and they manifest at 40 and this person is let’s say 10 years of age, how do you interpret that kind of remote risk? If genes have variable expressivities penetrance, how do you explain the risk to a person? So, these are big issues and people don’t want to be told you know there is a likely hood of this but we are not sure. It becomes a very big problem in informed consent | | | | | | | | | | | | | | | |  |
|  |  | | | | | | | | | | | | | | | |  |
|  |  | | | | | | | | | | | | | | | |  |
|  | | | | | | | | | | | |  |  |  |  |  |  |
|  | People want to be told definite terms and of course sometimes even the researchers don’t know (I: hmm) and hence the equipoise generating the research | | | | | | | | | | | | | | | |  |
|  |  | | | | | | | | | | | | | | | |  |
| Reports\\Coding Summary By Code Report | | | | | | | | | | Page 7 of 16 | | | | | | | |
|  | | | | | | | | | | | | | | | | | |
|  | | | **Aggregate** |  | **Classification** |  | **Coverage** |  | **Number Of Coding References** | |  | **Reference Number** |  | **Coded By Initials** |  | **Modified On** |  |
|  | | | | | | | | | | | | | | | | | |
|  | | | | | | | | | | | |  |  |  |  |  |  |
|  | Then ahh one last ethical issue I will bring about is about benefit sharing. That has become a very big problem there is a lot of benefits to accrue from genetics research. Issues to do with intellectual property rights particularly patents. We know that successful products of genomic research have far reaching effects. They have far reaching effects, they have far reaching economic potential and many times they are being studied on vulnerable persons who are so poor who are in poor social circumstances and they are being done by conglomerates, by pharmaceutical companies. They come from rich nations and they do rip big out of this. | | | | | | | | | | | | | | | |  |
|  |  | | | | | | | | | | | | | | | |  |
|  | | | | | | | | | | | |  |  |  |  |  |  |
|  | Then ahh one last ethical issue I will bring about is about benefit sharing. That has become a very big problem there is a lot of benefits to accrue from genetics research. Issues to do with intellectual property rights particularly patents. We know that successful products of genomic research have far reaching effects. They have far reaching effects, they have far reaching economic potential and many times they are being studied on vulnerable persons who are so poor who are in poor social circumstances and they are being done by conglomerates, by pharmaceutical companies. They come from rich nations and they do rip big out of this. | | | | | | | | | | | | | | | |  |
|  |  | | | | | | | | | | | | | | | |  |
|  | | | | | | | | | | | |  |  |  |  |  |  |
|  | So, the question is ahh we don’t have anything cast in stone. So, the theme of exploitation comes up, aren’t we exploiting these ahh vulnerable persons? Why are we involving them in this research? Why are we leaving them? And the institutions of course, institutions of where the researches are being done and the countries are vulnerable themselves (I: hmm). So, the theme of vulnerability, exploitation ahh post trial issues and access. To me they are very big ethical issues that must be discussed. | | | | | | | | | | | | | | | |  |
|  |  | | | | | | | | | | | | | | | |  |
|  |  | | | | | | | | | | | | | | | |  |
|  | | | | | | | | | | | |  |  |  |  |  |  |
|  | I: Yeah for as long as they are not identified, they don’t have identifiers bit that was what it was in the US but I think it changed with the revision of the common rule. (R: yeah) yeah now they have changed it because they realised that it was being exploited by researchers (R: yeah).  R: So, there should be limitations after the research is over and also as we have said before in this country, we are using the broad consent model. That is already a limitation and ahh questions have been about limitations on duration of storage, whether it should be perpetual. And I think the best practice here should be to design it at the informed consent aspect forever and that kind of stuff. Laughs, then there has been an incident on it (I: hmm). Ahh we had an investigator who got consent for storage of milk ahh he did a study on (I: milk) breast milk looking for microbiota (I: microbiota yeah). So, after doing this study on microbiota, ahh the investigator did not get consent (door opens) to pool the blood. So, after the analysis had been done another aspect of the study was to pulled the milk together and export it to Vienna (I: hmm). So, they mixed the milk together and then they came to get an MTA to take the pulled breast milk for export and that’s when we discovered we didn’t have regulatory guidance on pulling of specimens. Now when we inquired with the community representatives, they said ohh that’s a “tabu” (literally meaning problem) in this area. You don’t mix the breast milk from different people in one pool and maybe if the participants had known that their breast milk is going to be mixed, they wouldn’t have agreed (I: hmm). So, you see those cultural concerns, something may look simple to the scientists. So, for example somebody working in here would put a limitation that I am collecting breast milk but, in this culture, you should not pull the breast milk of people. They will feel like your mixing their lives together or something like that (I: and disrespecting them) and disrespecting them. In the same way, we know that in our communities here, people share blood as a sign of bonding and friendship, so if you told them that your mixing their blood, they could feel offended. So, there some of those limitations. What do you do with the specimens? Things like pulling them, storing them, the duration of storage and then future use being subjected to research ethics committee. Then another limitation that should be clear in the protocol is about the financial use. | | | | | | | | | | | | | | | |  |
|  |  |  |  |  |  |  |  |  |  |  |  |  |  |  |  |  |  |
|  |  |  |  |  |  |  |  |  |  |  |  |  |  |  |  |  |  |
|  |  | | | | | | | | | | | | | | | |  |
|  | | | | | | | | | | | |  |  |  |  |  |  |
|  | I: Hmm commercialization rights  R: Yeah, I think donors will feel offended if they voluntarily gave in specimens to advance science only to discover that the specimens were being sold for commercial purposes. I think there should be a limitation on financial use, commercialization of these ahh (I: specimen) samples. | | | | | | | | | | | | | | | |  |
|  |  | | | | | | | | | | | | | | | |  |
|  | | | | | | | | | | | | | | | | | |
| Reports\\Coding Summary By Code Report | | | | | | | | | | Page 8 of 16 | | | | | | | |
|  | | | | | | | | | | | | | | | | | |
|  | | | **Aggregate** |  | **Classification** |  | **Coverage** |  | **Number Of Coding References** | |  | **Reference Number** |  | **Coded By Initials** |  | **Modified On** |  |
|  | | | **Files\\Researchers\\male\\IDI Transcript 003 male** | | | | | | | | | | | | | |  |
|  |  |  | Yes |  |  |  | 0.0244 |  | 2 | |  | | | | | |  |
|  | | |  |  |  |  |  |  |  | |  | | | | | | |
|  | | | | | | | | | | | |  |  |  |  |  |  |
|  | R: People can be taken advantage of research which is done, I always see a lot of research takes place in developing countries (I: hmm) and I can see just like it was in the case of HIV/ AIDS (I: hmm). When we first started doing the interventions studies in HIV/AIDS a lot of research was taking place in the developing world but when it came to benefits (I: hmm) most of the benefits were not reachable to the individuals in the developing countries. So I can almost imagine in such a scenario in this kind of world, where a lot of genetic research is taking place in developing countries but when the remedies, when the cures of such research come out (I: hmm) they are almost not accessible to the people (I: hmm) in developing countries (I: coughs). So those are some of the risks (I: hmm, okay) and the challenges that are brought about by genetics research. | | | | | | | | | | | | | | | |  |
|  |  |  |  |  |  |  |  |  |  |  |  |  |  |  |  |  |  |
|  |  | | | | | | | | | | | | | | | |  |
|  | | | **Files\\Researchers\\male\\IDI Transcript 005 male** | | | | | | | | | | | | | |  |
|  |  |  | Yes |  |  |  | 0.0295 |  | 3 | |  | | | | | |  |
|  | | |  |  |  |  |  |  |  | |  | | | | | | |
|  | | | | | | | | | | | |  |  |  |  |  |  |
|  | Respondent: well, there are two instances, two major issues that I have seen. One is that the genomics studies whereas the samples taken for a particular aspect of study, there is always a possibility that something else can be done, which has not been consented for by the patient because the nature of the sample is that it can be used as wide as possible to experiment/for investigation as much as possible. It makes it a little bit of a challenge. | | | | | | | | | | | | | | | |  |
|  |  | | | | | | | | | | | | | | | |  |
|  | | | | | | | | | | | |  |  |  |  |  |  |
|  | The other aspect is the understanding. People do not understand this genomics we are talking about. So it always needs a lot of explanation to the patient and even when you explain to them, it is still new to them. So most of the time they will just agree or just disagree without understanding. So for me, those are the two aspects, the understanding by the participant and then the possibility of using whatever sample you have for infinite investigation without seeking further permission. | | | | | | | | | | | | | | | |  |
|  |  | | | | | | | | | | | | | | | |  |
|  | and then the possibility of using whatever sample you have for infinite investigation without seeking further permission. | | | | | | | | | | | | | | | |  |
|  |  | | | | | | | | | | | | | | | |  |
|  | | | **Files\\Researchers\\male\\IDI Transcript 009 male** | | | | | | | | | | | | | |  |
|  |  |  | Yes |  |  |  | 0.0086 |  | 1 | |  | | | | | |  |
|  | | |  |  |  |  |  |  |  | |  | | | | | | |
|  | Respondent: yes, there are a number of issues associated with genetic studies. genetic studies that link the patient information to where they are coming from tend to show the prevalence of the condition and that can buy us any other person about where the participants are located | | | | | | | | | | | | | | | |  |
|  |  | | | | | | | | | | | | | | | |  |
|  | | | | | | | | | | | | | | | | | |
| Reports\\Coding Summary By Code Report | | | | | | | | | | Page 9 of 16 | | | | | | | |
|  | | | **Aggregate** |  | **Classification** |  | **Coverage** |  | **Number Of Coding References** | |  | **Reference Number** |  | **Coded By Initials** |  | **Modified On** |  |
|  | | | **Files\\Researchers\\male\\IDI Transcript 012 male** | | | | | | | | | | | | | |  |
|  |  |  | Yes |  |  |  | 0.0430 |  | 1 | |  | | | | | |  |
|  | | |  |  |  |  |  |  |  | |  | | | | | | |
|  | R: so, sometimes you are reviewing a study which is going to look at some specific aspects related to some conditions and it could be individual, or it could be societal. Some of the results of the results of the study may affect the whole society not only the individual. And one has to careful if you are going to review such studies. About the end point, the impact into the individual research participant in the first place and then also what about the other aspects of the society? For example, somebody may be doing a study that eventually involves some tribes with certain particular issues and then you come up with a conclusion that the whole of that tribe maybe they die early. They die at 30s or 40s so they say we cannot marry from that tribe because if you marry from there your children will be dying at a certain age. Therefore, you bring up a whole big issue and then also the individual participant, you could be studying something for example in a pregnant woman that may have an impact on the unborn child later on. So, they have to be taken care of. So, those are some of the challenges which you meet when you are going to study, and it could be more general than very specific. But the general aspect after the publication of the result may have a bigger impact. | | | | | | | | | | | | | | | |  |
|  |  |  |  |  |  |  |  |  |  |  |  |  |  |  |  |  |  |
|  |  | | | | | | | | | | | | | | | |  |
|  | | | **Files\\Researchers\\male\\IDI Transcript 016 male** | | | | | | | | | | | | | |  |
|  |  |  | Yes |  |  |  | 0.1217 |  | 4 | |  | | | | | |  |
|  | | |  |  |  |  |  |  |  | |  | | | | | | |
|  | R: One of the bigger ahh I will mention maybe one or two (I: okay) I think the problem with genetics research is that you have your samples and can be kept much much longer than the time the person who consented for you to use those samples can leave. Other wise you can go to the bank or lab and pick samples and work. So, the discomfort there is that it is possible if there was an unethical researcher and lands on the samples they can do research without consent. Then the other issue which is the main issue is that in most cases, researchers ask respondents to consent for a single use instead of maybe asking for multiple use by different studies. Because you can ask and say do you mind if I use for this research and others that may be of this nature? Somebody says yes, or somebody can say no but if you have a researcher who is not well trained and not very ethical, they may say I have landed on these samples I can now use them for my post graduates. So, I think for positively thinking, I would recommend as a standard for the bigger benefit of those harvested genes that they should always put in a clause to ask for more use than single use of a study so that there is maximum benefit of the information or the sample that you already have. | | | | | | | | | | | | | | | |  |
|  |  |  |  |  |  |  |  |  |  |  |  |  |  |  |  |  |  |
|  |  | | | | | | | | | | | | | | | |  |
|  |  | | | | | | | | | | | | | | | |  |
|  |  | | | | | | | | | | | | | | | |  |
|  | R: I am not saying there is misuse of samples or data (I: hmm) I am only saying there is a probability. There is an open probability, so I think there is almost no possibility that patients can get to know. How do they know? They can’t know. Once they have gone you can’t know that’s why it has to be a trust and you don’t breach. The only way someone can know is if you landed on the label of your sample number and it is published in a book or something because you have the identifier then you say sample number XYZ is actually tagged to me, so this is my sample. Of course, that is not how data is published. The other possibility maybe the research coordinator can choose to say let me see the ethics of the entire process, the storage process. You store the sample and then there is a log which has name attached to the specific sample. If for sample reason there is laxity or carelessness, they are supposed to keep those things under lock and key. Then its possible that someone can know then they can say find out for me whether this person has sickle cell because I want to marry her (both laugh). She is involved in a study then you say aha then you steal a key and the coordinator checks and says ahh do you already have a child with this person? No (I: laughs) okay you are lucky that she is sickle cell. Then you go and tell the whole village I mean all your friends that that lady has sickle cell don’t get close to her. If you get close to her don’t get closer to her because you are promoting sickle cell. Yeah | | | | | | | | | | | | | | | |  |
|  |  |  |  |  |  |  |  |  |  |  |  |  |  |  |  |  |  |
|  |  | | | | | | | | | | | | | | | |  |
|  | | | | | | | | | | | | | | | | | |
| Reports\\Coding Summary By Code Report | | | | | | | | | | Page 10 of 16 | | | | | | | |
|  | | | **Aggregate** |  | **Classification** |  | **Coverage** |  | **Number Of Coding References** | |  | **Reference Number** |  | **Coded By Initials** |  | **Modified On** |  |
|  | | | | | | | | | | | | | | | | | |
|  | | | | | | | | | | | | 4 |  | DES |  | 4/16/2021 3:36 PM |  |
|  | R: In my view there aren’t big risks other than those ethical. If you participate instead there be some benefits instead. I don’t think their bigger risks other than the social risks like now you lose a partner because somebody has known more about you and your formation and you can predict what you are for the future because of what they have investigated. That would be a problem and you may have social shame maybe if your information is not well kept. Or if the people like counsellors get to access your information or if they are not well guarded on your information. It may be unpleasant for people to know about your formation. There aren’t a lot of risks, but you could of course get violence ah domestic violence if you lied that you don’t have XY and for some reason the information lands and you say eh. How could you not tell me about this and you wanted us to first tie a knot? You are a traitor so that Is the type of society, so it is more social but also physical and that would be as a result of ethics not being kept. Maybe the procedures are uncomfortable depending upon the site for the samples so that is another. Any invasive method caries a risk. So, a risk can be as easy as discomfort and also can be as complicated if the out come of the investigation doesn’t go well. | | | | | | | | | | | | | | | |  |
|  |  |  |  |  |  |  |  |  |  |  |  |  |  |  |  |  |  |
|  |  | | | | | | | | | | | | | | | |  |
|  | | | **Files\\Researchers\\male\\IDI Transcript 017 male** | | | | | | | | | | | | | |  |
|  |  |  | Yes |  |  |  | 0.0595 |  | 2 | |  | | | | | |  |
|  | | |  |  |  |  |  |  |  | |  | | | | | | |
|  | R: I think the ethical concerns in this area are we are dealing with genetic material from a participant and this genetic material can be linked to a cluster of individuals, families and communities. And also, it is unknown, so a lot can be derived from it that neither the participants nor the scientists might anticipate. Yeah so, I think that is an ethical issue that needs to be considered and of course we have heard of people who have patented genetic material DNA like the masai mara. Someone patented it without the consent of the owners of the DNA. So there legal concerns definitely and these place genetic research a little different from the other forms of research. | | | | | | | | | | | | | | | |  |
|  |  | | | | | | | | | | | | | | | |  |
|  | R: Yes, export and sharing of samples with foreign collaborators is a challenge because once the sample is exported, you lose control over the sample. But here again it depends on the integrity of the researcher because if the researcher has integrity, chances are that they are going to be collaborating with people who have integrity. But as a REC or as a regulator, we should never take this for granted. We should be able to look at the material transfer agreement and look at the clauses that burr the third parties from misusing these samples. And also, be able to know that in these material agreements, the people who share clearly in it are clearly aware and have taken any legal concerns and rather how they would be processed if they are rather told. | | | | | | | | | | | | | | | |  |
|  |  | | | | | | | | | | | | | | | |  |
|  | | | **Files\\Researchers\\male\\IDI Transcript 018 male** | | | | | | | | | | | | | |  |
|  |  |  | Yes |  |  |  | 0.0699 |  | 2 | |  | | | | | |  |
|  | R: I think now the biggest challenge is now in the studies, many investigators are putting objectives for genomics, sometimes do it for the sake of harvesting blood and store without a clear objective that they want to address now or in the near future especially studies that come from the big networks like EDCTP. A study might be on something else and then just put in an objective to store blood to store for the future unknown genetics research and it’s becoming more common and for me I find it a challenge because sometimes you don’t want to stop the study because they have added some objectives, sometimes you try to argue with them about the validity of doing that for every study even when the main objective is not around that and it’s increasingly becoming more common at least for the big networks. | | | | | | | | | | | | | | | |  |
|  |  | | | | | | | | | | | | | | | |  |
|  | R: For me I think that it would not be a bad idea if there was some degree of mutual consent cross fertilization, so if you want samples to study a certain condition and those samples, I have those samples in my repository there should be mechanism for us to share those samples and also share the scientific fruits that come out of those samples but if I need to study something and you have those samples if I’m an American and you have those samples, there should be room to access those samples for you to do the studies that I want to do here. So, in principle it shouldn’t be a problem but how to make it work so that it benefits the investigators on both sides of the Atlantic it’s what has been difficult and it’s usually because of how has the money and therefore who owns the data. | | | | | | | | | | | | | | | |  |
|  |  | | | | | | | | | | | | | | | |  |
|  | | | | | | | | | | | | | | | | | |
| Reports\\Coding Summary By Code Report | | | | | | | | | | Page 11 of 16 | | | | | | | |
|  | | | **Aggregate** |  | **Classification** |  | **Coverage** |  | **Number Of Coding References** | |  | **Reference Number** |  | **Coded By Initials** |  | **Modified On** |  |
|  | **Nodes\\B. Knowledge\B2. Ethical legal and social implications\Ethical\Benefit sharing** | | | | | | | | | | | | | | | |  |
|  | | **Document** | | | | | | | | | | | | | | |  |
|  | | | **Files\\Researchers\\male\\IDI Transcript 001 male** | | | | | | | | | | | | | |  |
|  |  |  | Yes |  |  |  | 0.0387 |  | 1 | |  | | | | | |  |
|  | | |  |  |  |  |  |  |  | |  | | | | | | |
|  | Respondent: I think one of the ethical issues is in regard to consenting because when you are talking about genetics, it means that you are no longer focusing on the individual per say, but several generations. because genetics really transcends from individuals- families, to communities then generations. So, when you are consenting some body, for the information that can be attributed from one person to the rest of the family and not part of the study, that is a serious ethical issue. Then the other thing is that genetic research could reveal things that are not at that time presentable to the individual implying that the implication of genetic research may be way and out of the mind and eyes of individual that is participating in research. The third issue could be that the outcome of genetic research is something that is not clearly appreciated. If I provide my genetic information and something develops out of it, how I am supposed to accrue of the reality that comes from such research?  Interviewer: so basically, that is about sharing of benefits  Respondent: sharing of benefits, yeah | | | | | | | | | | | | | | | |  |
|  |  |  |  |  |  |  |  |  |  |  |  |  |  |  |  |  |  |
|  |  | | | | | | | | | | | | | | | |  |
|  | | | **Files\\Researchers\\male\\IDI Transcript 002 male** | | | | | | | | | | | | | |  |
|  |  |  | Yes |  |  |  | 0.0174 |  | 1 | |  | | | | | |  |
|  | | |  |  |  |  |  |  |  | |  | | | | | | |
|  | Then ahh one last ethical issue I will bring about is about benefit sharing. That has become a very big problem there is a lot of benefits to accrue from genetics research. Issues to do with intellectual property rights particularly patents. We know that successful products of genomic research have far reaching effects. They have far reaching effects, they have far reaching economic potential and many times they are being studied on vulnerable persons who are so poor who are in poor social circumstances and they are being done by conglomerates, by pharmaceutical companies. They come from rich nations and they do rip big out of this. | | | | | | | | | | | | | | | |  |
|  |  | | | | | | | | | | | | | | | |  |
|  | | | **Files\\Researchers\\male\\IDI Transcript 016 male** | | | | | | | | | | | | | |  |
|  |  |  | Yes |  |  |  | 0.0375 |  | 1 | |  | | | | | |  |
|  | | |  |  |  |  |  |  |  | |  | | | | | | |
|  | | | | | | | | | | | |  |  |  |  |  |  |
|  | R: One of the bigger ahh I will mention maybe one or two (I: okay) I think the problem with genetics research is that you have your samples and can be kept much much longer than the time the person who consented for you to use those samples can leave. Other wise you can go to the bank or lab and pick samples and work. So, the discomfort there is that it is possible if there was an unethical researcher and lands on the samples they can do research without consent. Then the other issue which is the main issue is that in most cases, researchers ask respondents to consent for a single use instead of maybe asking for multiple use by different studies. Because you can ask and say do you mind if I use for this research and others that may be of this nature? Somebody says yes, or somebody can say no but if you have a researcher who is not well trained and not very ethical, they may say I have landed on these samples I can now use them for my post graduates. So, I think for positively thinking, I would recommend as a standard for the bigger benefit of those harvested genes that they should always put in a clause to ask for more use than single use of a study so that there is maximum benefit of the information or the sample that you already have. | | | | | | | | | | | | | | | |  |
|  |  |  |  |  |  |  |  |  |  |  |  |  |  |  |  |  |  |
|  |  | | | | | | | | | | | | | | | |  |
|  | | | | | | | | | | | | | | | | | |
|  | | | | | | | | | | | | | | | | | |
| Reports\\Coding Summary By Code Report | | | | | | | | | | Page 12 of 16 | | | | | | | |
|  | | | **Aggregate** |  | **Classification** |  | **Coverage** |  | **Number Of Coding References** | |  | **Reference Number** |  | **Coded By Initials** |  | **Modified On** |  |
|  | **Nodes\\B. Knowledge\B2. Ethical legal and social implications\Ethical\Commercialization** | | | | | | | | | | | | | | | |  |
|  | | **Document** | | | | | | | | | | | | | | |  |
|  | | | **Files\\Researchers\\male\\IDI Transcript 002 male** | | | | | | | | | | | | | |  |
|  |  |  | Yes |  |  |  | 0.0087 |  | 1 | |  | | | | | |  |
|  | | |  |  |  |  |  |  |  | |  | | | | | | |
|  | I: Hmm commercialization rights  R: Yeah, I think donors will feel offended if they voluntarily gave in specimens to advance science only to discover that the specimens were being sold for commercial purposes. I think there should be a limitation on financial use, commercialization of these ahh (I: specimen) samples. | | | | | | | | | | | | | | | |  |
|  |  | | | | | | | | | | | | | | | |  |
|  | | | **Files\\Researchers\\male\\IDI Transcript 003 male** | | | | | | | | | | | | | |  |
|  |  |  | Yes |  |  |  | 0.0244 |  | 1 | |  | | | | | |  |
|  | | |  |  |  |  |  |  |  | |  | | | | | | |
|  | R: People can be taken advantage of research which is done, I always see a lot of research takes place in developing countries (I: hmm) and I can see just like it was in the case of HIV/ AIDS (I: hmm). When we first started doing the interventions studies in HIV/AIDS a lot of research was taking place in the developing world but when it came to benefits (I: hmm) most of the benefits were not reachable to the individuals in the developing countries. So I can almost imagine in such a scenario in this kind of world, where a lot of genetic research is taking place in developing countries but when the remedies, when the cures of such research come out (I: hmm) they are almost not accessible to the people (I: hmm) in developing countries (I: coughs). So those are some of the risks (I: hmm, okay) and the challenges that are brought about by genetics research. | | | | | | | | | | | | | | | |  |
|  |  |  |  |  |  |  |  |  |  |  |  |  |  |  |  |  |  |
|  |  | | | | | | | | | | | | | | | |  |
|  | | | **Files\\Researchers\\male\\IDI Transcript 018 male** | | | | | | | | | | | | | |  |
|  |  |  | Yes |  |  |  | 0.0345 |  | 1 | |  | | | | | |  |
|  | | |  |  |  |  |  |  |  | |  | | | | | | |
|  | R: For me I think that it would not be a bad idea if there was some degree of mutual consent or cross fertilization, so if you want samples to study a certain condition and those samples, I have those samples in my repository there should be mechanism for us to share those samples and also share the scientific fruits that come out of those samples but if I need to study something and you have those samples if I’m an American and you have those samples, there should be room to access those samples for you to do the studies that I want to do here. So, in principle it shouldn’t be a problem but how to make it work so that it benefits the investigators on both sides of the Atlantic it’s what has been difficult and it’s usually because of how has the money and therefore who owns the data. | | | | | | | | | | | | | | | |  |
|  |  | | | | | | | | | | | | | | | |  |
|  | | | | | | | | | | | | | | | | | |
|  | | | | | | | | | | | | | | | | | |
|  | | | | | | | | | | | | | | | | | |
| Reports\\Coding Summary By Code Report | | | | | | | | | | Page 13 of 16 | | | | | | | |
|  | | | **Aggregate** |  | **Classification** |  | **Coverage** |  | **Number Of Coding References** | |  | **Reference Number** |  | **Coded By Initials** |  | **Modified On** |  |
|  | **Nodes\\B. Knowledge\B2. Ethical legal and social implications\Ethical\Deviation** | | | | | | | | | | | | | | | |  |
|  | | **Document** | | | | | | | | | | | | | | |  |
|  | | | **Files\\Community\\male\\IDI Transcript 011 male** | | | | | | | | | | | | | |  |
|  |  |  | Yes |  |  |  | 0.0195 |  | 1 | |  | | | | | |  |
|  | | |  |  |  |  |  |  |  | |  | | | | | | |
|  | | | | | | | | | | | |  |  |  |  |  |  |
|  | what we call ethical considerations of research must be followed. And should they be faulted individuals should be held accountable. That is the best way. So, the safest way is to add a clause in our research, that should a researcher fault guidelines, particularly ethical guidelines including even science as well. Because if science guidelines say that you put 2 millimeters [of blood] and you put 4, you are faulting the guidelines. And also, you are doing a wrong science and you are being unethical.  So, my point is that community can be safeguarded in two ways: one, a clause be put on the researchers that faulting ethical guidelines, faulting science guidelines, you are punishable. So that anybody doing research is aware that if I was supposed to take 10 samples and you take 15 samples, you are faulting guidelines. If you are supposed to monitor every week, then for you you monitor once in a month, eventually some people die because of…………. So, faulting ethical and science guidelines people should be held accountable. | | | | | | | | | | | | | | | |  |
|  |  |  |  |  |  |  |  |  |  |  |  |  |  |  |  |  |  |
|  |  | | | | | | | | | | | | | | | |  |
|  | **Nodes\\B. Knowledge\B2. Ethical legal and social implications\Ethical\Exploitation** | | | | | | | | | | | | | | | |  |
|  | | **Document** | | | | | | | | | | | | | | |  |
|  | | | **Files\\Researchers\\male\\IDI Transcript 002 male** | | | | | | | | | | | | | |  |
|  |  |  | Yes |  |  |  | 0.0822 |  | 2 | |  | | | | | |  |
|  | | |  |  |  |  |  |  |  | |  | | | | | | |
|  | | | | | | | | | | | |  |  |  |  |  |  |
|  | So, the question is ahh we don’t have anything cast in stone. So, the theme of exploitation comes up, aren’t we exploiting these ahh vulnerable persons? Why are we involving them in this research? Why are we leaving them? And the institutions of course, institutions of where the researches are being done and the countries are vulnerable themselves (I: hmm). So, the theme of vulnerability, exploitation ahh post trial issues and access. To me they are very big ethical issues that must be discussed. | | | | | | | | | | | | | | | |  |
|  |  | | | | | | | | | | | | | | | |  |
|  | | | | | | | | | | | |  |  |  |  |  |  |
|  | I: Yeah for as long as they are not identified, they don’t have identifiers bit that was what it was in the US but I think it changed with the revision of the common rule. (R: yeah) yeah now they have changed it because they realised that it was being exploited by researchers (R: yeah).  R: So, there should be limitations after the research is over and also as we have said before in this country, we are using the broad consent model. That is already a limitation and ahh questions have been about limitations on duration of storage, whether it should be perpetual. And I think the best practice here should be to design it at the informed consent aspect forever and that kind of stuff. Laughs, then there has been an incident on it (I: hmm). Ahh we had an investigator who got consent for storage of milk ahh he did a study on (I: milk) breast milk looking for microbiota (I: microbiota yeah). So, after doing this study on microbiota, ahh the investigator did not get consent (door opens) to pull the blood. So, after the analysis had been done another aspect of the study was to pulled the milk together and export it to Vienna (I: hmm). So, they mixed the milk together and then they came to get an MTA to take the pulled breast milk for export and that’s when we discovered we didn’t have regulatory guidance on pooling of specimens. Now when we inquired with the community representatives, they said ohh that’s a “tabu” (literally meaning problem) in this area. You don’t mix the breast milk from different people in one pool and maybe if the participants had known that their breast milk is going to be mixed, they wouldn’t have agreed (I: hmm). So, you see those cultural concerns, something may look simple to the scientists. So, for example somebody working in here would put a limitation that I am collecting breast milk but, in this culture, you should not pull the breast milk of people. They will feel like your mixing their lives together or something like that (I: and disrespecting them) and disrespecting them. In the same way, we know that in our communities here, people share blood as a sign of bonding and friendship, so if you told them that your mixing their blood, they could feel offended. So, there some of those limitations. What do you do with the specimens? Things like pulling them, storing them, the duration of storage and then future use being subjected to research ethics committee. Then another limitation that should be clear in the protocol is about the financial use. | | | | | | | | | | | | | | | |  |
|  |  |  |  |  |  |  |  |  |  |  |  |  |  |  |  |  |  |
|  |  |  |  |  |  |  |  |  |  |  |  |  |  |  |  |  |  |
|  |  | | | | | | | | | | | | | | | |  |
| Reports\\Coding Summary By Code Report | | | | | | | | | | Page 14 of 16 | | | | | | | |
|  | | | | | | | | | | | | | | | | | |
|  | | | **Aggregate** |  | **Classification** |  | **Coverage** |  | **Number Of Coding References** | |  | **Reference Number** |  | **Coded By Initials** |  | **Modified On** |  |
|  | | | **Files\\Researchers\\male\\IDI Transcript 003 male** | | | | | | | | | | | | | |  |
|  |  |  | Yes |  |  |  | 0.0244 |  | 1 | |  | | | | | |  |
|  | | |  |  |  |  |  |  |  | |  | | | | | | |
|  | | | | | | | | | | | |  |  |  |  |  |  |
|  | R: People can be taken advantage of research which is done, I always see a lot of research takes place in developing countries (I: hmm) and I can see just like it was in the case of HIV/ AIDS (I: hmm). When we first started doing the interventions studies in HIV/AIDS a lot of research was taking place in the developing world but when it came to benefits (I: hmm) most of the benefits were not reachable to the individuals in the developing countries. So I can almost imagine in such a scenario in this kind of world, where a lot of genetic research is taking place in developing countries but when the remedies, when the cures of such research come out (I: hmm) they are almost not accessible to the people (I: hmm) in developing countries (I: coughs). So those are some of the risks (I: hmm, okay) and the challenges that are brought about by genetics research. | | | | | | | | | | | | | | | |  |
|  |  |  |  |  |  |  |  |  |  |  |  |  |  |  |  |  |  |
|  |  | | | | | | | | | | | | | | | |  |
|  | **Nodes\\B. Knowledge\B2. Ethical legal and social implications\Ethical\genetic material** | | | | | | | | | | | | | | | |  |
|  | | **Document** | | | | | | | | | | | | | | |  |
|  | | | **Files\\Researchers\\male\\IDI Transcript 017 male** | | | | | | | | | | | | | |  |
|  |  |  | Yes |  |  |  | 0.0281 |  | 1 | |  | | | | | |  |
|  | | |  |  |  |  |  |  |  | |  | | | | | | |
|  | | | | | | | | | | | |  |  |  |  |  |  |
|  | R: I think the ethical concerns in this area are we are dealing with genetic material from a participant and this genetic material can be linked to a cluster of individuals, families and communities. And also, it is unknown, so a lot can be derived from it that neither the participants nor the scientists might anticipate. Yeah so, I think that is an ethical issue that needs to be considered and of course we have heard of people who have patented genetic material DNA like the masai mara. Someone patented it without the consent of the owners of the DNA. So there legal concerns definitely and these place genetic research a little different from the other forms of research. | | | | | | | | | | | | | | | |  |
|  |  | | | | | | | | | | | | | | | |  |
|  | **Nodes\\B. Knowledge\B2. Ethical legal and social implications\Ethical\Storage indefinitely** | | | | | | | | | | | | | | | |  |
|  | | **Document** | | | | | | | | | | | | | | |  |
|  | | | **Files\\Community\\male\\IDI Transcript 015 male** | | | | | | | | | | | | | |  |
|  |  |  | Yes |  |  |  | 0.0406 |  | 1 | |  | | | | | |  |
|  | | |  |  |  |  |  |  |  | |  | | | | | | |
|  | | | | | | | | | | | |  |  |  |  |  |  |
|  | I know when you take off these sample, for now the reason for taking off these samples could be something very small but as you keep the samples, and something comes giving them off could be something very small. You know science is evolving, an idea comes people can quickly go to those samples and then use them. And like you are saying you see how you create another Deborah and Deborah has no idea. For me this is somebody who has no idea, and this is someone who ahh for me this is a big issue and I don’t know how the gates can be made very tight. because I am also aware that the moment  a participant or myself i give any type of sample. I know I may sign certain things I am consenting to but I know the moment just like when I give  you information, you may promise me certain things but there is a level to which i cannot go. For me ethically that would be my big concern. what are we going to do to be sure that what they said because for me creating another one will not be a big problem if i know what is happening or if this participant who consented knows what you are going to do and the implication of what will happen there after. | | | | | | | | | | | | | | | |  |
|  |  |  |  |  |  |  |  |  |  |  |  |  |  |  |  |  |  |
|  |  | | | | | | | | | | | | | | | |  |
| Reports\\Coding Summary By Code Report | | | | | | | | | | Page 15 of 16 | | | | | | | |
|  | | | | | | | | | | | | | | | | | |
|  | | | **Aggregate** |  | **Classification** |  | **Coverage** |  | **Number Of Coding References** | |  | **Reference Number** |  | **Coded By Initials** |  | **Modified On** |  |
|  | | | **Files\\Researchers\\female\\IDI Transcript 007 female** | | | | | | | | | | | | | |  |
|  |  |  | Yes |  |  |  | 0.0124 |  | 1 | |  | | | | | |  |
|  | | |  |  |  |  |  |  |  | |  | | | | | | |
|  | | | | | | | | | | | |  |  |  |  |  |  |
|  | Respondent: the time I have served on the research and ethics committee, the main issue has been around storing and indefinitely using somebody’s genetic material and the requirement of the investigators for the research participants to sign giving authority to investigators to do blind ended research on their stored specimen and at the point of the consent it is not clear what else is going to be done in future. | | | | | | | | | | | | | | | |  |
|  |  | | | | | | | | | | | | | | | |  |
|  | | | **Files\\Researchers\\male\\IDI Trancript 006 male** | | | | | | | | | | | | | |  |
|  |  |  | Yes |  |  |  | 0.0309 |  | 1 | |  | | | | | |  |
|  | | |  |  |  |  |  |  |  | |  | | | | | | |
|  | | | | | | | | | | | |  |  |  |  |  |  |
|  | Respondent: i guess it is mainly that. I do not think there is any other main ethical issue but sometimes when we collect samples, for most of the genetic studies, they request to store samples and conduct further studies as needed and sometimes the ethical boards have no control on those samples and different laboratories can work on different samples and experiments like those about gene deletion can be done or performed and with such practices from the legal angle, you can introduce mutations that can affect different groups of people or populations and you can cause diseases. so it is complex | | | | | | | | | | | | | | | |  |
|  |  | | | | | | | | | | | | | | | |  |
|  | | | **Files\\Researchers\\male\\IDI Transcript 016 male** | | | | | | | | | | | | | |  |
|  |  |  | Yes |  |  |  | 0.0375 |  | 1 | |  | | | | | |  |
|  | | |  |  |  |  |  |  |  | |  | | | | | | |
|  | | | | | | | | | | | |  |  |  |  |  |  |
|  | R: One of the bigger ahh I will mention maybe one or two (I: okay) I think the problem with genetics research is that you have your samples and can be kept much much longer than the time the person who consented for you to use those samples can leave. Other wise you can go to the bank or lab and pick samples and work. So, the discomfort there is that it is possible if there was an unethical researcher and lands on the samples they can do research without consent. Then the other issue which is the main issue is that in most cases, researchers ask respondents to consent for a single use instead of maybe asking for multiple use by different studies. Because you can ask and say do you mind if I use for this research and others that may be of this nature? Somebody says yes, or somebody can say no but if you have a researcher who is not well trained and not very ethical, they may say I have landed on these samples I can now use them for my post graduates. So, I think for positively thinking, I would recommend as a standard for the bigger benefit of those harvested genes that they should always put in a clause to ask for more use than single use of a study so that there is maximum benefit of the information or the sample that you already have. | | | | | | | | | | | | | | | |  |
|  |  |  |  |  |  |  |  |  |  |  |  |  |  |  |  |  |  |
|  |  | | | | | | | | | | | | | | | |  |
|  | | | **Files\\Researchers\\male\\IDI Transcript 018 male** | | | | | | | | | | | | | |  |
|  |  |  | Yes |  |  |  | 0.0354 |  | 1 | |  | | | | | |  |
|  | | |  |  |  |  |  |  |  | |  | | | | | | |
|  | | | | | | | | | | | |  |  |  |  |  |  |
|  | R: I think now the biggest challenge is now in the studies, many investigators are putting objectives for genomics, sometimes do it for the sake of harvesting blood and store without a clear objective that they want to address now or in the near future especially studies that come from the big networks like EDCTP. A study might be on something else and then just put in an objective to store blood to store for the future unknown genetics research and it’s becoming more common and for me I find it a challenge because sometimes you don’t want to stop the study because they have added some objectives, sometimes you try to argue with them about the validity of doing that for every study even when the main objective is not around that and it’s increasingly becoming more common at least for the big networks. | | | | | | | | | | | | | | | |  |
|  |  | | | | | | | | | | | | | | | |  |
|  | | | | | | | | | | | | | | | | | |
| Reports\\Coding Summary By Code Report | | | | | | | | | | Page 16 of 16 | | | | | | | |
