# Supplementary material for: Benefit sharing in genomic and biobanking research in Uganda: Perceptions of researchers and research ethics committee members
Source: Front Genet. 2022 Nov 17;13:1037401. doi: 10.3389/fgene.2022.1037401 (PMC9714451; doi:10.3389/fgene.2022.1037401)
Supplement: Supplementary file 2 [file Table2.DOCX]

Interview guide form Researchers and REC chairs

**Socio-demographic questions**

1. Age________
2. Gender________
3. Occupation
4. Role on research ethics committee
5. How long have you been employed by your institution/REC? ___________
6. Highest education completed:
7. Master’s degree
8. Doctoral degree (M.D., PhD or equivalent
9. Other (describe)__________________________________
10. Primary discipline/field of your highest education? (e.g. Medicine specify speciality, psychology, epidemiology biostatistics etc.)
11. Primary area of research_________________________________________________
12. Please describe your training or education in research ethics
13. **Understanding of genomic related information by participants**
14. What is your experience of conveying genomic related information to participants?
15. Probe for ease of translation and language
16. Challenges with technical concepts
17. Do you know of any innovative ways that have been developed to help participants better understand genomic? Please explain
18. How can participants be helped to better understand genomic concepts?
19. **Informed Consent in genomic research and biobanking**
20. Describe the different types of consent
21. What type of consent is most suitable for genomic research and bio-banking? Give reasons for your answer.
22. What information should be included in an informed consent form for genomic research and biobanking?
23. Are there any situations where participants should be able to change their minds about the use of their samples or data in medical research? If so, in which situations would this be OK and what do you think would be the consequences?
24. Withdraw of consent
25. Probe for withdraw of samples
26. What risks can result from a breach in privacy and confidentiality?
27. What steps should be taken to safeguard the privacy and confidentiality of participants and their stored samples and associated data?
28. Describe some of the challenges that you have experienced in the informed consent process for genomic research?
29. Suggest ways of mitigating these challenges.
30. **Biological sample storage, and ownership**
31. What do you think of storing samples for future research? Probe about where the HBM can be stored?
32. In your opinion who owns biological samples and associated data? Why? Please explain.
33. What do you think about the export and sharing of samples with foreign collaborators
34. Probe for MTAs and their significance
35. Describe how the export of samples can be regulated
36. What is the best way to discuss the consequences of sample/data sharing to research participants?
37. In your opinion how should the result of genomic testing be handled?
    1. Probe for feedback of genomic results, including incidental/secondary results to research participants.
38. **Sharing of the benefits of genomic research and biobanking**
39. What are the benefits of genetics/genomic research and biobanking?
40. What are the challenges to benefit sharing in genomic research and biobanking?
41. How should the benefits of research be shared? (probe for researchers/research institutions, research participants and research communities)
42. How can fair sharing of the benefits of genomic and biobanking research be enhanced?
